# Dereplication of Natural Product Antifungals via Liquid Chromatography–Tandem Mass Spectrometry and Chemical Genomics

**DOI:** 10.3390/molecules30010077

**Published:** 2024-12-28

**Authors:** Nathaniel J. Brittin, David J. Aceti, Doug R. Braun, Josephine M. Anderson, Spencer S. Ericksen, Scott R. Rajski, Cameron R. Currie, David R. Andes, Tim S. Bugni

**Affiliations:** 1 Pharmaceutical Sciences Division, University of Wisconsin-Madison, Madison, WI 53705, USA; nbrittin@wisc.edu (N.J.B.); drbraun1@wisc.edu (D.R.B.); janderson49@wisc.edu (J.M.A.); scott.rajski@wisc.edu (S.R.R.); 2 Lachman Institute for Pharmaceutical Development, University of Wisconsin-Madison, Madison, WI 53705, USA; 3 Small Molecule Screening Facility, UW Carbone Cancer Center, Madison, WI 53792, USA; djaceti@wisc.edu (D.J.A.); ssericksen@wisc.edu (S.S.E.); 4 Department of Biochemistry and Biomedical Sciences, M.G. DeGroote Institute for Infectious Disease Research, David Braley Centre for Antibiotic Discovery, McMaster University, Hamilton, ON L8S 4L8, Canada; curric7@mcmaster.ca; 5 Department of Bacteriology, University of Wisconsin-Madison, Madison, WI 53706, USA; 6 Department of Medical Microbiology and Immunology, School of Medicine and Public Health, University of Wisconsin, Madison, WI 53706, USA; dra@medicine.wisc.edu; 7 Department of Medicine, University of Wisconsin School of Medicine and Public Health, Madison, WI 53705, USA; 8 William S. Middleton Memorial VA Hospital, Madison, WI 53705, USA

**Keywords:** antifungal, natural products, mechanism of action, dereplication, chemical genomics, metabolomics, screening, drug discovery, targeted bioprocess, drug resistance

## Abstract

Recently expanded reports of multidrug-resistant fungal infections underscore the need to develop new and more efficient methods for antifungal drug discovery. A ubiquitous problem in natural product drug discovery campaigns is the rediscovery of known compounds or their relatives; accordingly, we have integrated Liquid Chromatography–Tandem Mass Spectrometry (LC-MS/MS) for structural dereplication and Yeast Chemical Genomics for bioprocess evaluation into a screening platform to identify such compounds early in the screening process. We identified 450 fractions inhibiting *Candida albicans* and the resistant strains of *C. auris* and *C. glabrata* among more than 40,000 natural product fractions. LC-MS/MS and chemical genomics were then used to identify those with known chemistry and mechanisms of action. The parallel deployment of these orthogonal methods improved the detection of unwanted compound classes over the methods applied individually.

## 1. Introduction

Invasive fungal infections have become an increasing global health concern, with escalating rates of infection by multi-drug resistant (MDR) strains and a limited number of antifungals for treatment [1,2,3,4]. This particularly impacts sensitive populations such as immunocompromised patients and organ transplant recipients, with mortality rates ranging from 30 to 95% and hospital stays extending from 46 to 140 days [2,5]. Treatments are limited to three classes of antifungal used for invasive fungal infections: polyenes, azoles, and echinocandins. Thus, the development of double and triple resistance by dangerous pathogens such as *Candida albicans*, *C. auris*, *C. glabrata*, and *Aspergillus fumigatus* is highly concerning [6,7,8]. Novel antifungals with new mechanisms are clearly needed.

**Figure 1:**
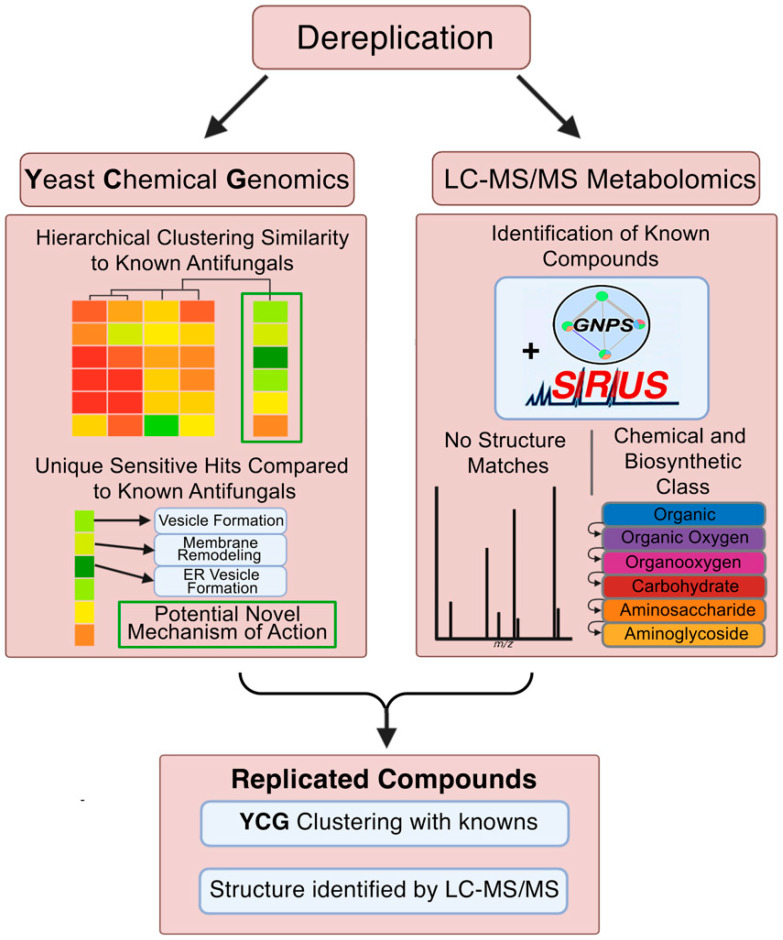
Identification and dereplication of antifungal natural product fractions using YCG and LC-MS/MS. Bacterial isolates from under-explored microbiomes were cultured. Cells and spent growth media were extracted, extensively fractionated, and assayed for inhibition of *Candida albicans* strain K1. Positive fractions were further screened against MDR strains *C. auris* B11211 and *C. glabrata* 4720. YCG and LC-MS/MS were then used in parallel to identify fractions with known or otherwise undesirable MoA profiles and chemical structures.

Addressing this urgent need, we present a novel discovery pipeline that integrates high-resolution Liquid Chromatography–Tandem Mass Spectrometry (LC-MS/MS) with Yeast Chemical Genomics (YCG) [9,10,11]. This innovative platform seamlessly combines the structural dereplication capabilities of LC-MS/MS [12,13] with the functional insights provided by YCG, offering an efficient approach to identify and characterize antifungal compounds. Central to our strategy is the prioritization of bacterial strains sourced from under-explored niches—marine invertebrates, insects, and human microbiomes—which are renowned for their rich diversity of unique metabolites [11,14]. Unlike conventional methods that often rely on single-dimension screens, our integrated approach simultaneously assesses both the compound structure and mechanism of action (MoA), enhancing discovery accuracy. The prioritized bacterial strains were processed to generate extracts, which underwent a two-step fractionation process to yield high-purity natural product fractions suitable for high-throughput screening [15]. Fractions were then screened at four concentrations against *Candida* albicans K1, followed by tests against MDR strains *C. glabrata* and *C. auris*, and counter-screened for hemolytic toxicity against red blood cells. Out of over 40,000 fractions screened, 450 samples displayed activity against MDR fungal pathogens. By leveraging the dual functionality of LC-MS/MS and YCG, our pipeline not only minimizes the rediscovery of known compounds but also focuses efforts on promising candidates by integrating structural data with MoA insights (Figure 1).

## 2. Results and Discussion

LC-MS/MS spectral data from extract fractions were analyzed using GNPS [16] and SIRIUS 5 (ver. 5.8.0) [17,18,19,20]; GNPS compares experimental data to ~600,000 molecule-annotated spectra whereas SIRIUS 5 employs database-independent structure predictions, thus expanding comparative abilities to >110,000,000 unique structures present in databases like PubChem and ChemSpider. In parallel, YCG links the action of antifungal compounds to the pathways of fungal biology and can provide mechanism of action (MoA) insights [21,22,23]. In YCG, pools of DNA-barcoded *Saccharomyces cerevisiae* single-gene knockout strains are grown in the presence of extract fractions with antifungal activity. Strain populations are then quantified by barcode DNA sequencing to generate lists of hypersensitive and resistant strains, which provides a chemical genomic profile. These characteristic profiles are then compared with those from known antifungals and other fractions for dereplication. To leverage YCG for our screening library, we optimized the system for use with 50 µL cultures in 384-well plates in a semi-automated fashion. Since each well in our fractionated library contained only ~100 µg of material, we opted to employ the optimized 384 well format over the more traditional 96 well format.

**Figure 2:**
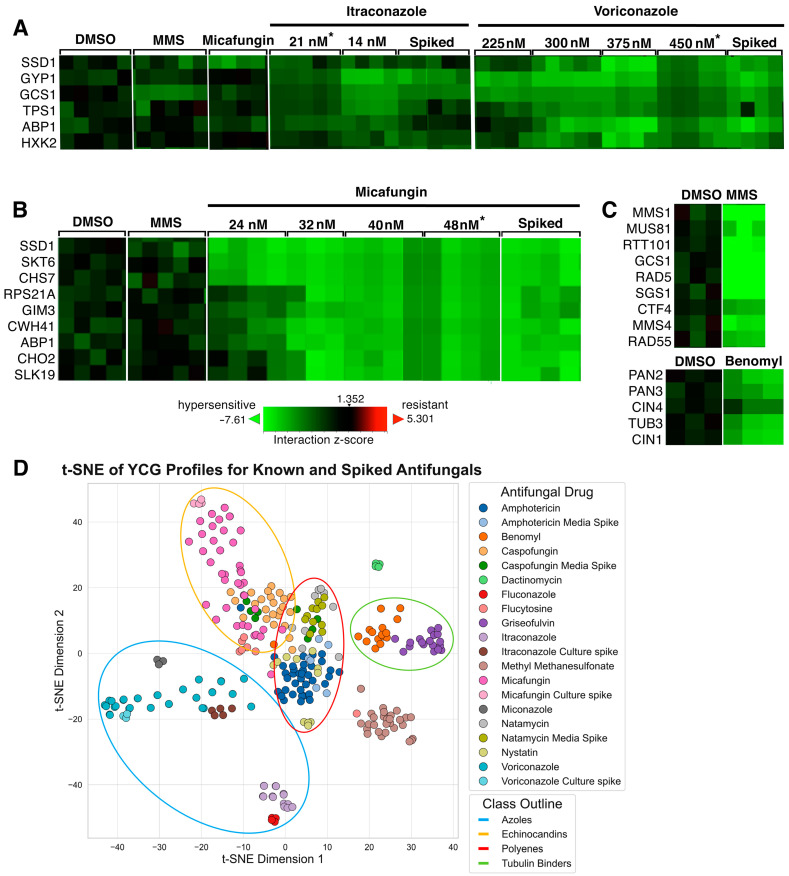
Detection by YCG of known antifungals in spiked cultures. Heatmaps indicate a spectrum of knockout strains (y-axes), from those with greatly diminished abundance following growth in the presence of antifungal compared to the DMSO control (bright green) to those that were unaffected (black); each column on the x-axes indicates a replicate of the indicated compound; 4–5 replicates were performed per condition, and occasionally, a replicate failed to produce data, resulting in fewer columns. Pearson correlation for the complete dataset consisting of four replicates of 287 strains is 0.69 (SD = 0.05) supporting YCG reproducibility. The strains shown are those most obviously correlated to antifungal activity out of 310 possible targets (see Supplementary Material Dataset S5). (**A**) Itraconazole-, voriconazole-, and (**B**) micafungin-spiked fraction heatmaps closely resembled those of each pure compound. Shown for comparison are heatmaps for negative control DMSO and positive control MMS (methyl methanesulfonate), benomyl, and micafungin, for the same genes. (**C**) The positive controls MMS and benomyl showed characteristic signals elsewhere in the heatmap. (**D**) The t-distributed Stochastic Neighbor Embedding (t-SNE) analysis of full YCG datasets (313 strains) for the pure and spiked antifungals included in this experiment (amphotericin B, natamycin, caspofungin, micafungin, itraconazole, and voriconazole) as well as a range of additional antifungals; the benzamidazole benomyl; the actinomycin dactinomycin; the pyrimidine analog flucytosine; the methanosulfonate ester MMS; the azole miconazole; and the polyene nystatin. * YCG signal intensities that diminish with increasing antifungal concentration are likely attributable to changes in cell populations associated with the total library growth inhibition exceeding 50%.

To evaluate LC-MS/MS and YCG for the reliable identification of known compounds in this system, we spiked bacterial cultures that were devoid of antifungal activity with a variety of clinically used antifungals, namely the polyenes amphotericin B and natamycin, the echinocandins caspofungin and micafungin, and the azoles itraconazole and voriconazole. Extraction and fractionation were followed by testing for antifungal activity against *C. albicans* to identify active wells, which were then analyzed by LC-MS/MS and queried via GNPS and SIRIUS 5 for the positive identification of the antifungals (Supplementary Material Datasets S1.1–S1.7). In parallel, active wells were tested by YCG using the “Diagnostic” library of 310 DNA-barcoded yeast knockouts [23]. After qPCR amplification, amplicons from all the wells of a 384-well plate were combined and sequenced. Sequence data were analyzed using BEAN-counter (ver. 2.6.1) [24]. The unique YCG output for each compound, a vector related to the growth of all the knockout strains, was grouped with others by similarity using hierarchical clustering; groupings were visualized using TreeView 3.0 (ver. 3, beta 1) [25] (Figure S1A). Spikes of itraconazole, voriconazole, and micafungin showed YCG profiles similar to pure compounds (Figure 2 and Figure S1). Short lists of the most hypersensitive (or occasionally, most resistant) strains, which we term “YCG Profiles”, were found to be replicably diagnostic of compounds or compound classes (Figure 2A–C, y-axes). YCG profiles sometimes reflected MoA, for example that of micafungin, which targets the cell wall, included the knockouts of the cell wall assembly and maintenance genes SSD1, SKT5, CHS7, and CWH41 (Figure 2B); that of positive control MMS (methyl methanesulfonate), a DNA-damaging agent, included the knockouts of the DNA repair and maintenance genes MMS1, MUS81, RTT101, RAD5, SGS1, CTF4, MMS4, and RAD55 (Figure 2C); that of positive control benomyl, which targets tubulin, included the knockouts of the tubulin and tubulin-folding genes TUB3, CIN1, and CIN4 (Figure 2C). Conversely, itraconazole and voriconazole did not induce hypersensitivity in any gene knockout directly related to their target, ergosterol synthesis (Figure 2A); we attribute this lack of connectivity between raw chemical genomic data and MoA, which is not infrequent in our experience to the complexity and interconnectivity of biological pathways.

Unlike the azoles and micafungin, the spiked caspofungin profile only partially matched that of the pure compound, and the spiked amphotericin B and natamycin did not cluster with their respective pure compounds or show similar heatmap profiles (Figures S2 and S3); in fact, new heatmap signatures appeared for spiked caspofungin and amphotericin B. We speculated that caspofungin, amphotericin B, and natamycin were modified by the bacterial culture, or that they stimulated the production of new compounds by the bacterial culture, or both. To test this hypothesis, each of the three compounds was spiked into uninoculated fractionated media; such spiked samples were devoid of bacterial exposure. Importantly, the spiked compound YCG profiles clustered with the respective pure compounds (Figure S3), thus validating the notion of microbial involvement in generating the unexpected new heatmap signatures. Dimensional reduction using t-SNE enabled the visualization of bioactivity clusters within the BEAN counter data [24]. The visualization of these distinct clusters of drug classes underscored YCG’s utility in differentiating among the MoAs characteristic of various antifungal classes. Notably, antifungals spiked in either the culture or media grouped closely with their corresponding pure compounds (Figure 2D), effectively clustering within their respective drug families.

**Figure 3:**
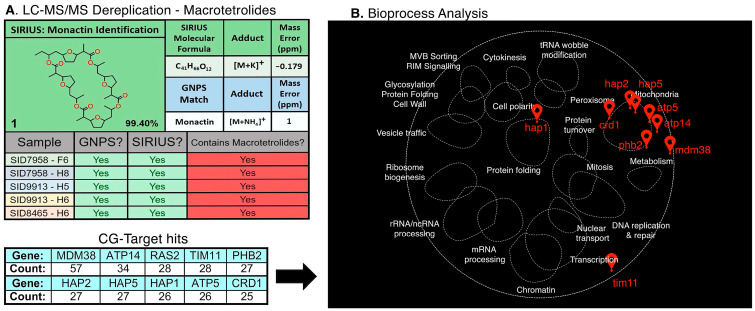
Identification of macrotetrolides by YCG and LC-MS/MS. (**A**) SIRIUS 5- and GNPS-driven analysis of the LC-MS/MS of the seven clustered fractions identified some combination of the macrotetrolides monactin, dinactin, trinactin, and nonactin in each. (**B**) CG-Target analysis of all 7 fractions and compilation of genes in the top 30 “Driver Scores” resulted in a grand total hit list of genes with largely mitochondrial/ion gradient functions, as illustrated in TheCellMap analysis.

Next, we assessed the YCG profiles for known compounds that were confidently identified by LC-MS/MS to ensure that the YCG profiles were consistent with compound class. As an example of the complementary nature of LC-MS/MS and YCG, we employed the macrotetrolide family of compounds. Macrotetrolides (monactin, dinactin, trinactin, tetranactin, or nonactin) were identified by LC-MS/MS in fractions H5 and H7 of an extract from the insect microbiome-derived strain SID7958 (Figure 3). By HCA analyses of the YCG profiles, five other fractions were found to have similar profiles (Figure S4A). All seven YCG profiles showed a similar signature with the following knockouts being diagnostic: SMY1, SUR1, SEC72, BST1, GIM3, GUP1, and SCS2 (Figure S4B,C). Importantly, targeted LC-MS/MS analyses enabled the identification of macrotetrolides in each of the five fractions where YCG indicated macrotetrolides (Figure 3A, Supplementary Material Datasets S2.1–S2.5).

Even though all seven fractions displayed similar YCG profiles, there was no convergence on one specific singular function or pathway. Therefore, we used the CG-Target software (ver. 0.6.1) to associate the YCG profiles to yeast bioprocesses by leveraging known genetic interactions across the whole yeast genome. CG-Target [26] uses a genome-wide interaction network (established via pairwise gene knockouts) to link chemical genomics data to likely impacted bioprocesses. The input of YCG data for the seven macrotetrolide fractions found a concentrated overlap of genes linked to mitochondrial function (Figure 3B), including the MDM38 mitochondrial K^+^/H^+^ exchange protein, the ATP14/TIM11/ATP5 subunits of mitochondrial F1F0 ATP synthase, and the HAP1/2/5 activators of respiratory gene expression. This network was visualized using TheCellMap [27], matching the profiles to mitochondrial bioprocesses (Figure 3B) in agreement with the known MoA of macrotetrolides, which are known to disrupt ion transport across the mitochondrial membrane.

Next, we evaluated the LC-MS/MS-YCG platform in characterizing polyenes. Polyenes such as amphotericin B, nystatin, and natamycin act primarily through their interaction with ergosterol and the subsequent formation of pores in fungal cell membranes [28,29,30]. Polyenes, in general, are associated with high toxicity and were consequently targeted for exclusion in our search for novel antifungals. The LC-MS/MS identification of polyenes is challenging as polyenes are prone to in-source fragmentation, vastly complicating the spectral analysis. Moreover, polyenes have exceptional potency that, in our experience, leads to bioactivity hits even when the concentrations of compounds are near or beyond LC-MS/MS detection limits. Additionally, MS/MS-based methods are convoluted by noise at low intensities and signal loss from in-source fragmentation. Although polyenes display distinctive UV-Vis spectra (Figure S5), this method is not sufficiently sensitive to identify them given their low concentrations in the majority of our working samples. Thus, considering these challenges and limitations, we evaluated the YCG datasets to help identify polyenes in hit wells based on their potent activity rather than physical detection methods.

**Figure 4:**
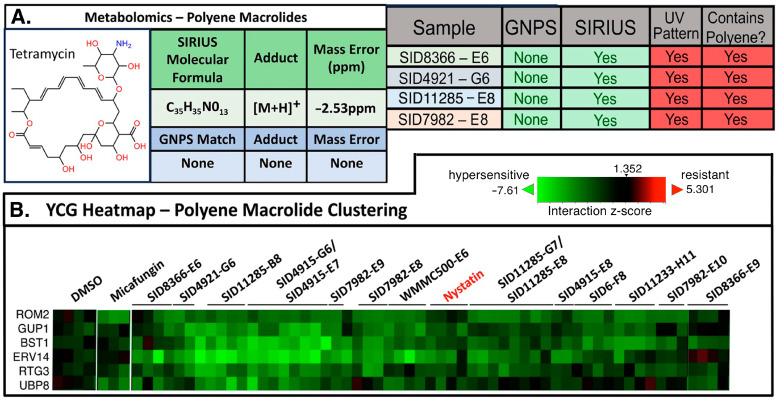
Identification of polyenes by LC-MS/MS, YCG, and UV spectroscopy. (**A**) Identification of the polyene antifungals by LC-MS/MS metabolomics processed using GNPS and SIRIUS 5, and by UV-Vis spectroscopy (Supplementary Material Figure S5, Dataset S3 for UV-Vis data). (**B**) Heatmap of the fractions clustering with the polyene nystatin in a YCG experiment. See Supplementary Material Dataset S5 for comprehensive heatmap data.

Using the distinct UV spectral pattern characteristic of polyenes, we confirmed the presence of polyene antifungals, and the LC-MS/MS metabolomics tool SIRIUS 5 was employed to predict the structure of the antifungal agent; in this fashion, polyenes were found in seven wells. On the basis of the HCA of the YCG profiles, those seven wells and eight others grouped with the polyene nystatin (Figure 4 and Supplementary Material Datasets S3 and S5.3). While the clustering of the YCG data helped identify other wells containing polyenes, the hypersensitive YCG profiles for polyenes were only moderately diagnostic. In general, polyenes show profiles consistent with an impact on vesicle-mediated trafficking; for example, the most sensitive mutant is Δ-ERV14, which is involved with vesicle formation. We have two hypotheses supporting the observed YCG profile for polyenes: 1. cell stress due to polyenes requires increased vesicle-mediated trafficking to help stabilize the cell wall; or 2. ergosterol plays a role in vesicle formation and amphotericin could be interacting with ergosterol in vesicles. Regardless, the ERV14 knockout was found to be consistently hypersensitive. Although the YCG signature is not as prominently defined as was the case for micafungin (Figure 2B), the combination of LC-MS/MS and YCG proved valuable for de-prioritizing wells containing polyenes.

## 3. Conclusions

In sum, this study demonstrated the effectiveness of combining LC-MS/MS and YCG in a high-throughput screening platform for antifungal drug discovery. Dereplication, as demonstrated in this work, results in the elimination from consideration of known or otherwise undesirable compounds and compound classes, saving time and resources that can go to novel compounds. In our platform, compounds devoid of structural or MoA data matches to known (and undesirable) antifungals are generally passed to medium-scale production for eventual in vivo assays and structure determination efforts. In short, such compounds/samples are given higher priority than might otherwise have been the case if not for the described dereplication initiative. Overarchingly, this approach will dramatically minimize the amount of time spent on deciphering known compounds, a common issue in natural products research. Although beyond the scope of this publication, some of the compounds/samples identified herein are currently the focus of more refined studies and will be reported in due course.

## 4. Materials and Methods

The materials, instruments, and methods used in this study are included in the Supplementary Materials.

## Data Availability

Data are contained within the article or Supplementary Materials.

## Supplementary Materials

The following information can be downloaded at: https://www.mdpi.com/article/10.3390/molecules30010077/s1. All experimentals (wet chemistry/biology and computation) [31,32,33]; Figure S1: HCA and expanded heatmap analysis of itraconazole, voriconizole, and micafungin and spiked fractions; Figure S2: YCG heatmap analysis of pure and culture-spiked caspofungin, amphotericin B, and natamycin; Figure S3: YCG heatmap and HCA analysis of pure and media-spiked caspofungin, amphotericin B, and natamycin; Figure S4: Identification of macrotetrolides by YCG; Figure S5: Detection of polyene macrolide antifungals by UV/Vis spectroscopy in pure compound stocks and complex bacterial extracts; Figure S6: Detection of polyenes by YCG and LC-MS/MS metabolomics using the SIRIUS 5 software suite; Dataset S1: Datasets for known antifungals (LC-HR-MS/MS, Sirius); Dataset S2: Dataset for macrotetrolide dereplication in complex bacterial extract fractions; Dataset S3: Dataset for polyenes identification in antifungal active extract fractions; Dataset S4: Dataset containing dereplication data (mirror plots) for samples from strains SID7958, SID8465 & SID9913; Dataset S5: Dataset containing comprehensive heatmap data supporting Figure 2, Figure 4 and Figures S1–S4.
