# Supplementary material for: Dereplication of Natural Product Antifungals via Liquid Chromatography–Tandem Mass Spectrometry and Chemical Genomics
Source: Molecules. 2024 Dec 28;30(1):77. doi: 10.3390/molecules30010077 (PMC11721837; doi:10.3390/molecules30010077)

Supplementary Materials:

Dereplication of Natural Product Antifungals via Liquid Chromatography–Tandem Mass Spectrometry and Chemical Genomics

Nathaniel J. Brittin[1,2,†], David J. Aceti[3,†], Doug R. Braun[1], Josephine M. Anderson[1], Spencer S. Ericksen[3], Scott R. Rajski[1], Cameron R. Currie[4,5], David R. Andes[6,7,8], and Tim S. Bugni[1,2,3]*

1. Pharmaceutical Sciences Division, University of Wisconsin−Madison, Madison, Wisconsin, United States
2. Lachman Institute for Pharmaceutical Development, University of Wisconsin–Madison, Madison, Wisconsin, United States
3. Small Molecule Screening Facility, UW Carbone Cancer Center, Madison, Wisconsin, United States
4. Department of Biochemistry and Biomedical Sciences, M.G. DeGroote Institute for Infectious Disease Research, David Braley Centre for Antibiotic Discovery, McMaster University, Hamilton, Ontario, Canada
5. Department of Bacteriology, University of Wisconsin-Madison, Madison, Wisconsin, USA
6. Department of Medical Microbiology and Immunology, School of Medicine and Public Health, University of Wisconsin, Madison, Wisconsin, United States
7. Department of Medicine, University of Wisconsin School of Medicine and Public Health, Madison, Wisconsin, United States
8. William S. Middleton Memorial VA Hospital, Madison, Wisconsin, United States

*Corresponding author, tim.bugni@wisc.edu

†These authors contributed equally

Table of Contents

Item Classification	Contents/Description	Page
Experimental Methods	Microbiome strain isolation and in vitro activity testing<br>Fermentations for Library Generation<br>Library Generation<br>High Throughput Screening<br>LC-MS/MS data collection<br>Metaboscape Software Application<br>LC-MS/MS Dereplication<br>Yeast Chemical Genomics data collection<br>CG-Target processing<br>TheCellMap processing<br>Spiked Bacterial Library Plates for YCG screening<br>t-SNE Analysis of BEAN-Counter Output	**S4**<br>**S4**<br>**S4**<br>**S5**<br>**S5**<br>**S6**<br>**S6**<br>**S6**<br>**S7**<br>**S8**<br>**S8**<br>**S8**
**Figure S1**	HCA and expanded heatmap analysis of itraconazole, voriconizole, and micafungin and spiked fractions.	**S9**
**Figure S2**	YCG heatmap analysis of pure and culture-spiked caspofungin, amphotericin B, and natamycin.	**S10**
**Figure S3**	YCG heatmap and HCA analysis of pure and media-spiked caspofungin, amphotericin B, and natamycin.	**S11**
**Figure S4**	Identification of macrotetrolides by YCG	**S12**
**Figure S5**	Detection of polyene macrolide antifungals by UV/Vis spectroscopy in pure compound stocks and complex bacterial extracts	**S13**
**Figure S6**	Detection of polyenes by YCG and LC-MS/MS metabolomics using the SIRIUS 5 software suite.	**S14 – S22**
**Datasets for known antifungals (LC-HR-MS/MS, Sirius)**	**Dataset S1.1** – Amphotericin B<br>**Dataset S1.2** – Griseofulvin<br>**Dataset S1.3** – Itraconazole<br>**Dataset S1.4** – Natamycin<br>**Dataset S1.5** – Micafungin<br>**Dataset S1.6** – Caspofungin<br>**Dataset S1.7** – Voriconazole	**S23 – S24**<br>**S24 – S25**<br>**S25 – S26**<br>**S26– S27**<br>**S27**<br>**S28 – S29**<br>**S29 – S31**
**Dataset for macrotetrolide dereplication in complex bacterial extract fractions.**	**Dataset S2.1** – Dereplication results for SID7958-F6<br>**Dataset S2.2** – Dereplication results for SID7958-H8<br>**Dataset S2.3** – Dereplication results for SID9913-F5<br>**Dataset S2.4** – Dereplication results for SID9913-H6<br>**Dataset S2.5** – Dereplication results for SID8465-H6	**S32 – S34**<br>**S34 – S37**<br>**S37 – S41**<br>**S41 – S44**<br>**S45 – S47**
**Dataset for polyenes identification in antifungal active extract fractions**	**Dataset S3** – Sample/fraction summary with retention times<br>LC-UV-MSMS spectra collected for fractions as follows:<br>SID4915 – E4<br>SID4921 – E6<br>SID4921 – E7<br>SID4921 – G7<br>SID7982 – G7<br>SID8366 – E9<br>SID8366 – G6<br>SID8366 – G7<br>SID11285 – E8<br>SID11285 – G7	**S48**<br><br>**S49**<br>**S50 – S52**<br>**S53 – S55**<br>**S56 – S59**<br>**S60**<br>**S61 – S62**<br>**S63 – S64**<br>**S65 – S68**<br>**S69 – S70**<br>**S71**

**Dataset containing dereplication data (mirror plots) for samples from strains SID7958, SID8465 & SID9913**	**Dataset S4.** – Dereplication based on actual MSMS datasets and data matching (via mirror plots and cosine similarity determinations) to GNPS databank files.<br>**Dataset S4.1** – Mirror plots for SID7958 – F6<br>**Dataset S4.2** – Mirror plots for SID7958 – H8<br>**Dataset S4.3** – Mirror plots for SID8465 – H6<br>**Dataset S4.4** – Mirror plots for SID9913 – H5<br>**Dataset S4.5** – Mirror plots for SID9913 – H6	**S72 – 76**<br>**S72**<br>**S73**<br>**S74**<br>**S75**<br>**S76**
**Dataset containing comprehensive heatmap data supporting Figures 2, 4, S1–S4.**	**Dataset S5.** Comprehensive heatmap details.<br>Color coding system and sample legend (correlating strain identifier to UW SMSF research code used to generate raw data)<br>**Dataset S5.1** – Full heatmap for experiments detailing YCG data for azole antifungals (**Figure 2A** in manuscript)<br>**Dataset S5.2 –** Full heatmap for experiments detailing YCG data for established antifungal (positive controls) micafungin and MMS (manuscript **Figure 2B**).<br>**Dataset S5.3** – Full heatmap for experiments detailing YCG data for suspected polyene containing samples alongside polyene standard nystatin and positive control micafungin (manuscript **Figure 4**).	**S77**<br>**S78**<br>**S79**<br>**S80**

Methods:

Microbiome strain isolation and in vitro activity testing:

Microbiome bacterial strains were collected, prioritized, fermented for production of compounds of interest, extracted, and fraction libraries generated as described from marine invertebrates [15], insects [11] and humans [31]. High throughput screening for growth inhibition of *Candida albicans* K1 was as described and active wells were tested against multiply drug-resistant *Candida auris* B11211 and *Candida glabrata* 4720 as well as for hemolysis and human cell line cytotoxicity [15].

Fermentation for Library Generation:

For each prioritized strain, 10 mL seed cultures (25 × 150 mm tubes) in medium DSC (5 g soluble starch, 10 g glucose, 5 g peptone, 5 g yeast extract per liter made with 50% artificial seawater) were inoculated and shaken (200 RPM, 28 °C) for seven days. Seed cultures (2.5 mL) were used to inoculate three 100 mL of media in 500 mL baffled flasks using two distinct media (2 X 100 mL ASW-A and 100 mL RAM2) containing Diaion HP20 (7% by weight). ASW-A was made using 20 g soluble starch, 10 g glucose, 5 g peptone, 5 g yeast extract, 5 g $CaCO_3$ per liter of artificial seawater; RAM2 was made using 4 g corn meal, 10 g glucose, 15 g maltose, 7.5 g pharmamedia, 5 g yeast per liter of 50% artificial seawater. After fermentation for 7 days, the cells and HP20 were filtered using Miracloth, and the cells and HP20 were extracted with acetone (100 mL for 30 min).

Library Generation:

The crude extract was dried and then dissolved using the following solvent mixture: 1 mL dimethyl sulfoxide (DMSO), 1 mL methanol, and 10 mL H_2O. Subsequently, the mixture was fractionated on an Isolute ENV+ (500 g cartridge) using a modified Gilson GX-271 liquid handler with 100% H_2O (10 mL), 25% CH_3OH/H_2O [fraction 1], 50% CH_3OH/H_2O [fraction 2], 75% CH_3OH/H_2O [fraction 3], 100% CH_3OH [fraction 4] (8 mL of each solvent). The 100% water fraction went directly to waste while the remaining four fractions were collected and subsequently dried in a speedvac. Each fraction was dissolved in DMSO and subjected to HPLC using a Gilson HPLC integrated with a Gilson 215 fitted with a 96-well plate deck capable of holding ten plates. For HPLC, a Phenomenex Monolithic C18 (3 mm ID X 100 mm) was used. The following HPLC gradients were used:

Fraction 1 (F1)

t = 0 → 2 min, 90% H_2O/10% CH_3CN
t = 14.5 → 14.51 min, 50% H_2O/50% CH_3CN
t = 19 → 21.5 min, 100% CH_3CN
t = 22 → 27 min, 90% H_2O/10% CH_3CN

Fraction 2 (F2) and Fraction 3 (F3)

t = 0 → 2 min, 90% H_2O/10% CH_3CN
t = 19 → 21.5 min, 100% CH_3CN
t = 22 → 27 min, 90% H_2O/10% CH_3CN

Fraction 4 (F4)

t = 0 → 2 min, 90% H_2O/10% CH_3CN
t = 5 → 5.01 min, 70% H_2O/30% CH_3CN
t = 19 → 32 min, 100% CH_3CN
t = 32.5 → 37.5 min 90% H_2O/10% CH_3CN

For each fraction above (F1–F4), 20 fractions were collected in 96-deepwell plates such that for each extract, metabolites were arrayed in 80 wells. Each plate was quantified using ELSD using previously published methods [15, 32]. The plates were dried in a speedvac. DMSO (20 µL) was added to each well to dissolve the material. The contents were then transferred to Labcyte Echo plates prior to high-throughput screening.

High Throughput Screening:

Next, in vitro high-throughput screening was applied to these HPLC purified fractions using a four-point dose response in 384 well plates with a Labcyte Echo 550 acoustic droplet delivery system (Agilent Technologies Inc., Santa Clara, CA, USA) against *Candida auris*. Assay plates for antimicrobial testing are made ahead of time, using the Echo 550 acoustic liquid handler. 500, 250, 100 and 50 nL of natural product fraction were transferred to each quadrant of a clear 384 well plate. The following control was used for *C. albicans* (Amphotericin B 0.5 mg/mL). To prepare the test organism, a single colony of *C. albicans* was picked from a solid agar plate into 5 mL of a liquid culture and was grown for 18 hours shaking at 37 °C. This O/N culture was diluted to 0.5 McFarland units, and this stock was further diluted 1:300 for use in HTS assays. Fifty µL per well of the diluted culture was added to each well of the 384 well assay plate using the ThermoFisher Multidrop instrument (Thermo Fisher Scientific Inc., Waltham, MA, USA). Microorganisms were incubated with the compound overnight at 37°C. Microorganism growth was measured by collecting an end point absorbance reading at OD_{600} using a BMG CLARIOstar plate reader (BMG Labtech Inc., Cary, NC, USA).

***LC-MS/MS*:**

Liquid chromatography tandem mass spectrometry (LC-MS/MS) data were acquired using a Bruker maXis II Ultra-High-Resolution LC-QTOF mass spectrometer (Bruker Scientific LLC., Billerica, MA, USA) coupled to a Waters Acquity H-Class UPLC system (Waters, Milford, MA, USA) and operated by the Bruker Hystar 3.2 software. Chromatographic gradients were performed with a mixture of methanol and water (containing 0.1% formic acid) on an RP C-18 column (Phenomenex Kinetex 2.6 µm, 2.1 mm × 100 mm; Phenomenex, Torrance, CA, USA) at 0.3 mL/min. The method was as follows: 0-1 min (10%-10% MeOH in H_2O), 1–12 min (10%–97% MeOH in H_2O), and 12–15.5 min (97% MeOH in H_2O). A mass range of m/z 50–1550 was measured in positive ESI mode for all spectra. The mass spectrometer was operated with the following parameters: capillary voltage of 4.5 kV, nebulizer pressure of 1.2 bar, dry gas flow of 4.0 L/min, dry gas temperature of 205 °C, and scan rate of 2 Hz. Tune mix (ESI-L low concentration; Agilent, Santa Clara, CA, USA) was introduced through a divert valve at the end of each chromatographic run for automated internal calibration. MS/MS spectra were acquired at scan speeds of 2 Hz for signals above 1 x 104 counts and 6 Hz for signals above 1 x 10^6 counts. MS/MS spectra were collected using a stepping collision energy (CE) where CE increased linearly during MS/MS spectra collection. From time 0 to 32, the collision RF was 600, transfer time was 80, and CE was 70eV. From time 33-66, the collision RF was 600, transfer time was 72, and CE was 100eV. From time 67-100, the collision RF was 600, transfer time was 65, and CE was 130eV. The precursor list was set to exclude precursor ions for 0.2 min after two spectra with the same precursor ion were acquired. Additionally, if the intensity of an excluded precursor ion rose fivefold from the initial spectra, it was recollected.

Metaboscape:
MetaboScape, a software developed by Bruker Compass (ver. 5.0.0, Build 683), was utilized to process raw LC-MS/MS data obtained from the Bruker maXis II LC-QTOF instrument (Bruker Scientific LLC., Billerica, MA, USA). To extract comprehensive information, T-ReX 3D (LC-QTOF) algorithm was employed with specific parameters. Initially, all samples underwent filtering with a value of 1 for both "Minimum # Features for Extraction" and "Presence of features in minimum # of analyses" settings. This filtering approach ensured the extraction of all features, including sample-specific features. The resulting filtered features were then subjected to further processing using customized T-ReX 3D parameters.

The customized T-ReX 3D processing parameters included an intensity threshold of 5000 counts, a minimum peak length of 7 spectra, feature signal based on intensity, enabled recursive feature extraction with a minimum peak length of 6 spectra for recursive processing, retention time range of 0.5 min to 16 min, and mass range of 50 m/z to 1550 m/z. Moreover, the software enabled the import and averaging of MS/MS data, and collision energy grouping was activated. Ion deconvolution was performed using the T-ReX Default Metabolomics Positive Ions settings. Additionally, mass recalibration was conducted using a customized T-ReX Positive Recalibration approach. The recalibration involved setting the retention time (RT) window to 17.5-20 min and utilizing the "Tuning Mix ES-TOF (ESI) Pos" as the list of calibrant signals. Upon successful bucketing of all LC-MS/MS data, data were exported using the “Export to GNPS” option.

LC-MS/MS Dereplication
From Metaboscape, the data is exported using the “Export to GNPS” option which includes the files needed for GNPS and SIRIUS 5 as mascot generic format (MGF) files. For GNPS the file is processed using their METABOLOMICS-SNETS-V2 (v. release_28). On the GNPS servers, the data was filtered by removing all MS/MS fragment ions within +/- 17 Da of the precursor m/z. MS/MS spectra were window filtered by choosing only the top 6 fragment ions in the +/- 50Da window throughout the spectrum. The precursor ion mass tolerance was set to 0.005 Da and a MS/MS fragment ion tolerance of 0.005 Da. A network was then created where edges were filtered to have a cosine score above 0.7 and more than 6 matched peaks. Further, edges between two nodes were kept in the network if and only if each of the nodes appeared in each other's respective top 10 most similar nodes. Finally, the maximum size of a molecular family was set to 100, and the lowest scoring edges were removed from molecular families until the molecular family size was below this threshold. The spectra in the network were then searched against GNPS' spectral libraries. The library spectra were filtered in the same manner as the input data. All matches kept between network spectra and library spectra were required to have a score above 0.7 and at least 6 matched peaks. For SIRIUS, the data was processed using SIRIUS 5 (ver. 5.8.0).

***Yeast Chemical Genomics (YCG)*:**
The 310-strain *S. cerevisiae* haploid non-essential gene “Diagnostic” knockout library was generously provided by Charles Boone and prepared by Jeff Piotrowski [23]. For each antifungal compound to be tested, varying concentrations were tested with the aim of finding some that resulted in 20–50% growth inhibition versus DMSO control after static growth for 17–24 h at 30°C. Importantly, optimum heatmap signatures generally correlate to growth inhibition levels < 50% [23]. Inhibition levels exceeding 50% can, depending on assorted factors, lead to muted

signal intensities [23]. Three concentrations including and bracketing the one determined above were used in the experiment; each condition was carried out in 4–5 replicates. In some cases, replicate experiments failed to produce data thus affording heatmap lane variances observed in Figures 2 and 4. DMSO-dissolved compounds were dispensed into 384- or 96-well flat-bottom plates using an Echo 650 Liquid Handler (Beckman-Coulter, Indianapolis, IN, USA). Library pool stocks containing approximately 250 cells/strain/µl were thawed, diluted 100-fold into YPD Broth (Thermo Fisher Scientific, Waltham, MA, USA), and 50 or 200 µl of diluted library was dispensed for 384-well or 96- well plates, respectively. OD_{600} was measured at 17–24 hours growth, and growth was continued to a final 48 hours before harvesting. Total genomic DNA was purified using a PureLink Pro 96 Genomic DNA Purification kit (Life Technologies, Carlsbad, CA, USA), AcroPrep 384-Well Filter Plates (VWR, Radnor, PA, USA), and Zymolase (Thermo Fisher Scientific, Hampton, NH, USA). Barcodes were amplified using indexed forward primers and a universal reverse primer as described [33]. Ten µl of each 25 µl reaction were pooled, loaded onto a 2% agarose gel, and the 267 bp product band was purified using a QIAquick Gel Extraction Kit (Qiagen, Germantown, MD, USA). A sample was submitted to the University of Wisconsin-Madison Biotechnology Center for sequencing on an Illumina (San Diego, CA, USA) MiSeq. Data was processed using BEAN-counter (ver. 2.6.1)[24], TreeView3 (ver. 3.0, beta-1)[25], CG-Target (ver. 0.6.1)[26], and TheCellMap.

An alternative barcode amplification/sequencing workflow with improved flexibility and enhanced cost effectiveness was developed and used in supporting experiments. Barcodes in the Diagnostic yeast library were amplified with one pair of non-indexed primers containing the same annealing sequences (underlined) as those used in the original method but with tails containing priming sites for a second round of PCR (bold) (Forward: 5'-**TCGTCGGCAGCGTC**AGATGTGTATAAG AGACAG<u>GATGTCCACGAGGTCTCT</u>-3', Reverse: 5'-**GTCTCGTGGGCTCGG**AGATGTGTA TAAGAGACAG<u>GCACGTCAAGACTGTCAAGG</u>-3'). In 15 µl reactions, 4.8 µl genomic DNA template was amplified (2 min 95°C, 30x[30s 95°C/30s 56°C/45s 68°C], 10 min 68° C), resulting in 238 bp products. A second round of PCR used 2 µl of this product as template in 20 µl reactions with pairs of indexed primers (Forward: 5'-AATGATACGGCGACC ACCGAGATCTACACNNNNNNNNNNTCGTCGGCAGCGTC-3', Reverse 5'-CAAGCAGAAG ACGGCATACGAGATNNNNNNNNNNGTCTCGTGGGCTCGG-3') that added two unique 10 bp index tags and Illumina P5 and P7 stems (5 min 95°C, 12x[60s 95°C/30s 57.5°C/45s 68°C], 10 min 68°C). A portion (5-10 µl/well) of the resulting 311 bp products were pooled and gel-purified as before. The dual unique index tags permitted inexpensive sequencing on a Illumina NovaSeq 6000 shared lane, and initial amplification with one pair of dedicated primers allows universal applicability of index primer pairs.

CG-Target:

CG-Target (ver. 0.6.1) was used essentially as described [26] resulting in, for each compound identity/concentration condition, multiple possible functional assignments as GO (Gene Ontology) terms; these are ranked by p-value, False Discovery Rate, z-score, Driver (gene or genes driving that assignment) Score, and Driver Name. The best condition for each compound, ideally resulting in 20-50% inhibition of the culture and highly negative CG scores indicating hypersensitive strains, was chosen for further analysis. The data associated with that condition was sorted by the first score in the Driver Score column and, for the thirty best (highest scoring)

rows, the most commonly occurring genes were extracted from the Driver Name column for a grand total count list, and the most common Gene Ontology terms were noted.

TheCellMap.org:

TheCellMap (version [at: https://thecellmap.org/?q=bni1] [27] was used by entering, into the main search field, lists of genes from genetic interaction networks that CG-TARGET found to resemble the hypersensitive/resistant profiles resulting from exposure to antifungal compounds. Concentrations of “hits’ in particular functional areas were taken as evidence for compound mechanism of action.

Spiked Bacterial Library Plates for YCG Screening:

Spiked library plates were prepared by culturing the bacterium WMMC1424 with the same methods as described in the “*Microbiome strain isolation and in vitro activity testing”*. WMMC1424 is a *Micromonospora* sp. SG15 with a 99.5% (1361/1368) 16S alignment score and WMMC1424 extract was previously shown to have no antifungal activity and no antifungal compounds were found using LC-MS/MS based metabolomics. To spike the extracts, 5 mg of each antifungal was added to the culture (each 1L in volume) 4 hours before extraction and left to shake to adhere to the extraction resin. The antifungals spiked into each 1L culture were natamycin, amphotericin B, voriconazole, itraconazole, caspofungin, and micafungin. Once the active wells were identified, they were analyzed by YCG and LC-MS/MS based metabolomics to confirm the presence of each spiked antifungal**.**

t-SNE Analysis of BEAN-Counter Output:

To perform t-SNE analysis, we first prepared a matrix of the YCG Profiles data, with rows representing experimental conditions or compound treatments (e.g., pure compounds, spiked samples, etc..) and columns representing responses across selected features (e.g., growth differentials in specific knockout strains). Data were normalized to standardize feature scales using the Robust Scaler within the scikit-learn preprocessing package. We applied t-distributed Stochastic Neighbor Embedding (t-SNE) using key parameters: perplexity (set to 10), iterations (750), and early exaggeration (18). The t-SNE was implemented using the scikit-learn library in Python, with parameter adjustments based on pilot runs to optimize visualization. The results were plotted with different colors to distinguish compound types (e.g., pure vs. spiked samples). The drugs of each antifungal class are outlined according to class to demonstrate the observable difference in the observed YCG profiles.

Figure S1. HCA and expanded heatmap analysis of itraconazole, voriconazole, and micafungin and spiked fractions. (**A**) Chemical genomics HCA of six pure antifungal compounds and the same compounds spiked into a bacterial culture. Only optimal data (compound concentrations giving 20-50% inhibition of yeast library cultures and favorable CG scores) were included. (**B**, **C**) Fig. 2 heatmaps expanded to include additional hits including resistant strains. Maximally hypersensitive and resistant regions of heatmaps and the corresponding strains are shown.

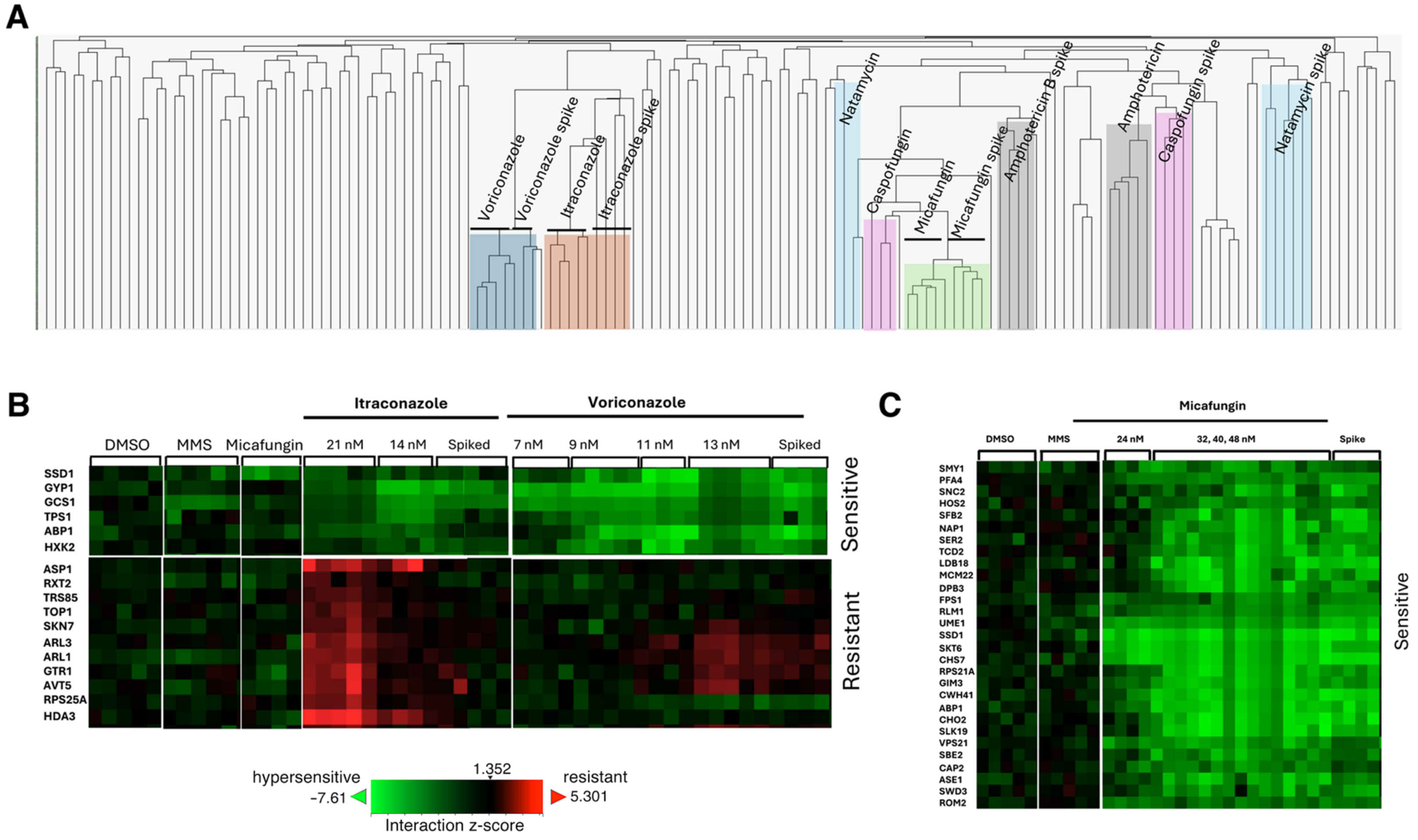

Figure S2. YCG heatmap analysis of pure and culture-spiked caspofungin, amphotericin B, and natamycin. After processing YCG data in BEAN-counter to determine whether pure-compound/spike pairs clustered within a larger dataset, each pair was individually processed in BEAN-counter along with controls to generate condensed heat maps showing dose-response. Maximally hypersensitive regions of heatmaps and the corresponding strains are shown.

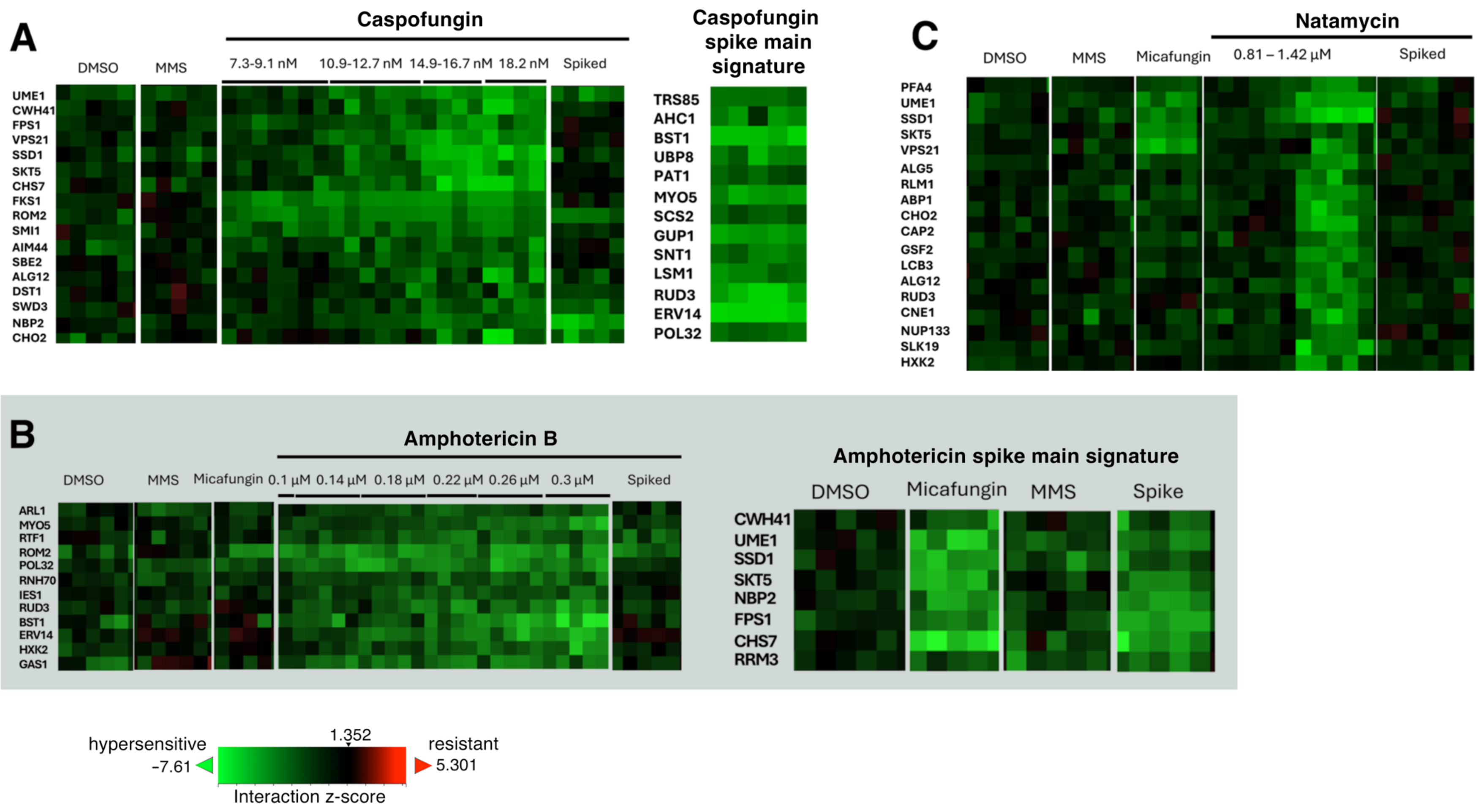

Figure S3. YCG heatmap and HCA analysis of pure and media-spiked caspofungin, amphotericin B, and natamycin. (**A**) Chemical genomics HCA of three antifungal compounds and the same compounds spiked into fractionated ASW-A medium. (**B**–**D**) After processing YCG data in BEAN-counter to determine whether pure-compound/spike pairs clustered within a larger dataset, each pair was individually processed in BEAN-counter along with controls to generate condensed heat maps (**B**: amphotericin, **C**: caspofungin, **D**: natamycin), Maximally hypersensitive regions of heatmaps and the corresponding strains are shown.

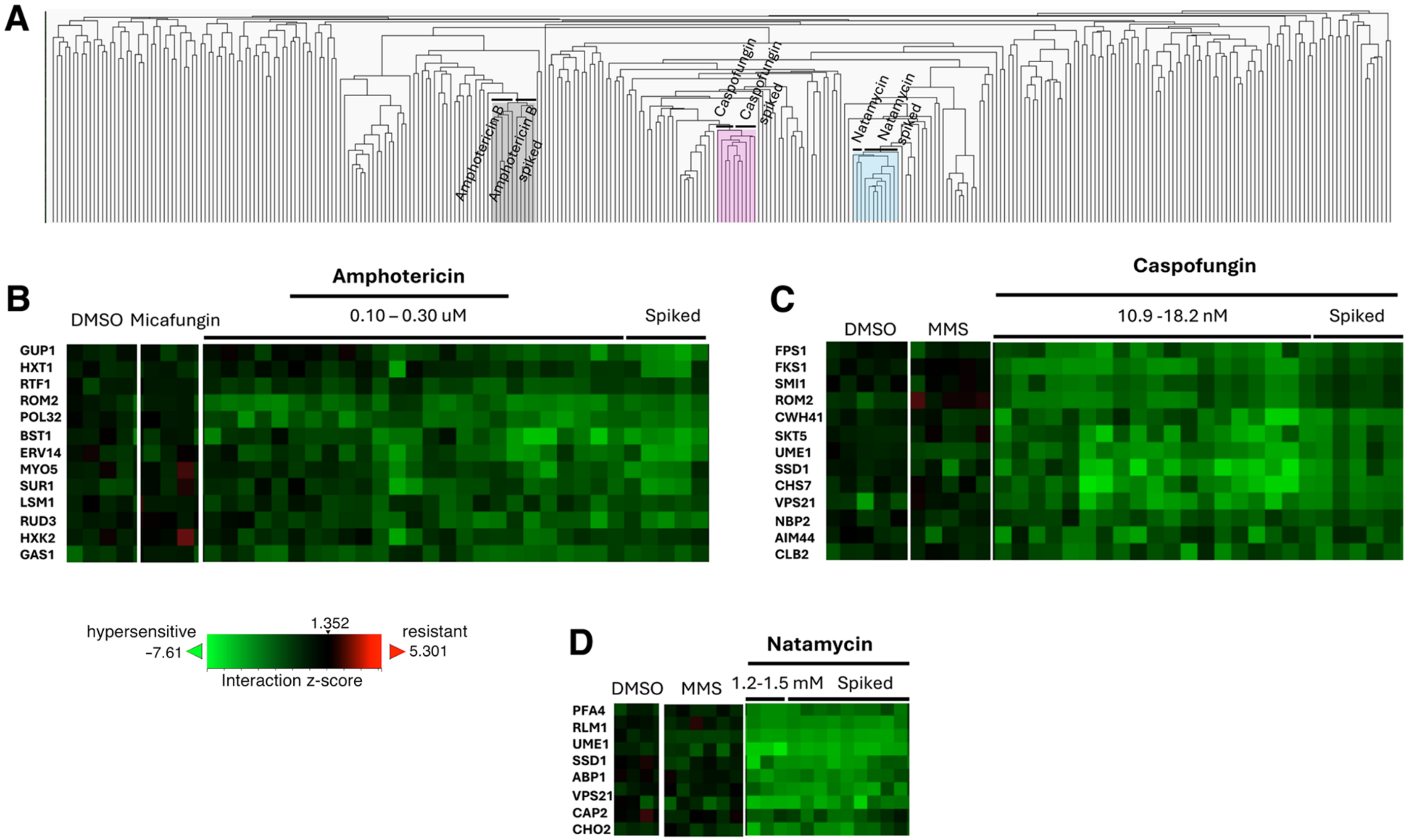

Figure S4. Identification of macrotetrolides by YCG. (**A**) HCA of YCG data for fractions with antifungal activity. Data for microbiome-derived fractions, negative control (carrier) DMSO, and the known antifungals flucytosine, benomyl, itraconazole, micronazole, voriconazole, fluconazole, amphotericin B, natamycin, caspofungin, micafungin, terbinafine, and MMS (methyl methanesulfonate) were analyzed and clustered using BEAN-counter and visualized in TreeView3. Fractions SID7958-H5 and H7, previously established by LC-MS/MS to contain macrotetrolides, clustered in the highlighted branch along with five other fractions. (**B**) The heatmap signature of the cluster is shown and compared with those of DMSO, MMS, and micafungin. (**C**) The seven fractions and each fraction's 10 most hypersensitive yeast knockout strains as identified via YCG.

A

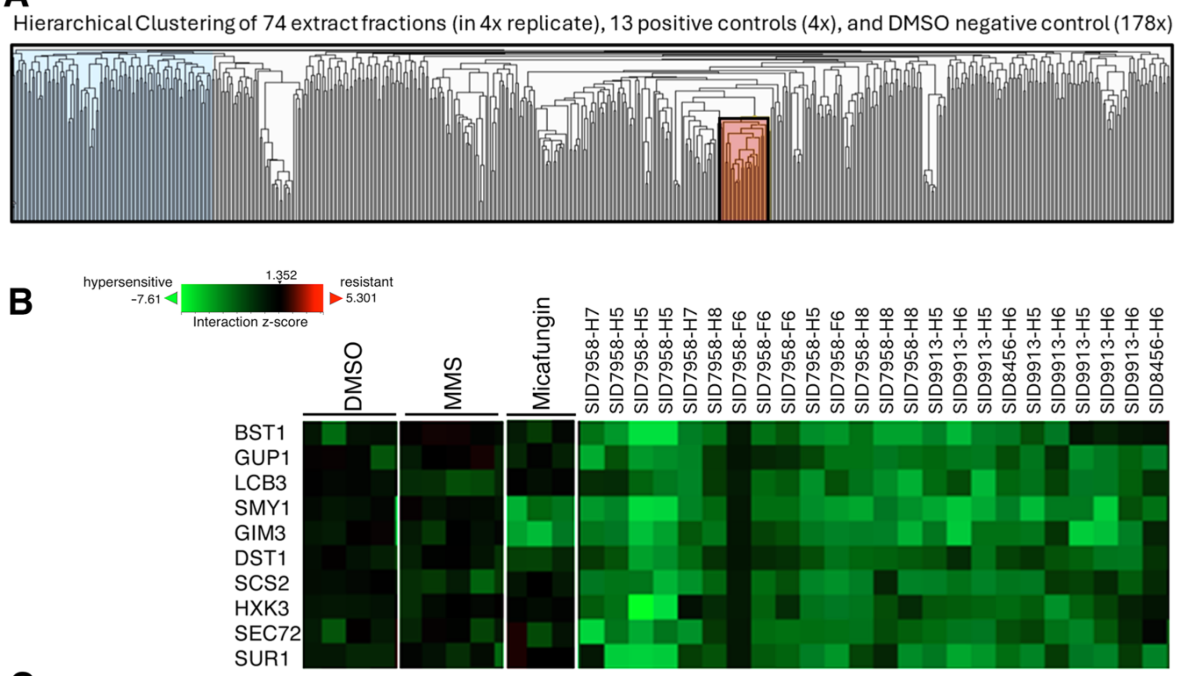

C

Extract Fraction-Well	YCG Strain Fitness									
SID7958-F6	SMY1	BST1	SUR1	SCS2	NUP100	SER1	SAE2	SEC72	HAC1	RIF1
SID7958-H5	SUR1	BST1	HXK2	SMY1	GIM3	SCS2	SEC72	CNE1	STP1	CHO2
SID7958-H7	SEC72	GUP1	SMY1	SCS2	ERV14	RRM3	BST1	CTF3	SAS4	CLB2
SID7958-H8	SMY1	GIM3	BST1	LCB3	HHF2	ERV14	ROM2	SCS2	GUP1	CHO2
SID8465-H6	SMY1	SUR1	CHO2	GUP1	CWC21	RPL9B	GNP1	ECM1	SCS2	ERV14
SID9913-H5	SMY1	GIM3	SUR1	LCB3	GUP1	ECM30	SCS2	CHO2	SEC72	BST1
SID9913-H6	GIM3	SMY1	ROM2	SEC72	GUP1	SUR1	MSB1	HXK2	RNH70	LCB3

Figure S5. UV/Vis spectroscopy of pure compound stocks and complex bacterial extracts. (**A**) Nystatin control, (**B**) Amphotericin B control, antifungal active bacterial extract fractions for [**C**] SID7982-E8, (**D**) SID4921-G6, (**E**) SID8366-E6, and (**F**) SID4921-E8. These data inform perceptions of Dataset 3 sample spectra and polyene likeness.

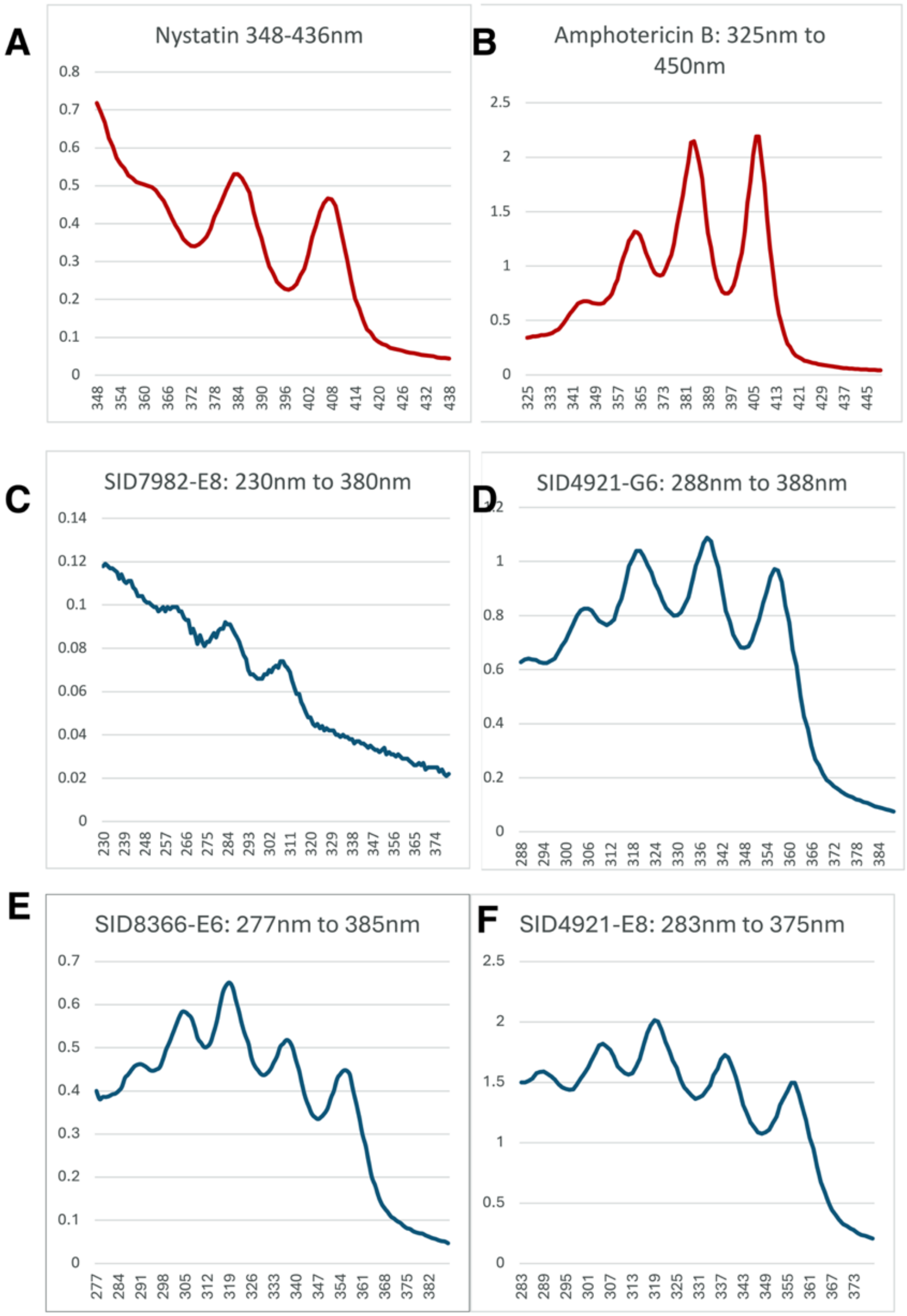

Figure S6. Detection of polyenes by YCG and LC-MS/MS metabolomics using the SIRIUS 5 software suite**.** (**A**) Identification of polyenes in natural product fractions by YCG and HCA. The same BEAN-counter/TreeView3 analysis used to cluster and identify fractions containing macrotetrolides in Figure S5 was used here to identify likely polyenes. Known polyene drugs and polyene identifications in experimental fractions by orthogonal techniques (UV/Vis spectroscopy or LC-MS/MS) or both are shown by colored type or highlighting (see Key). (**B**) Polyene Antifungal Dereplication in Complex Bacterial Extract Fractions (next page).

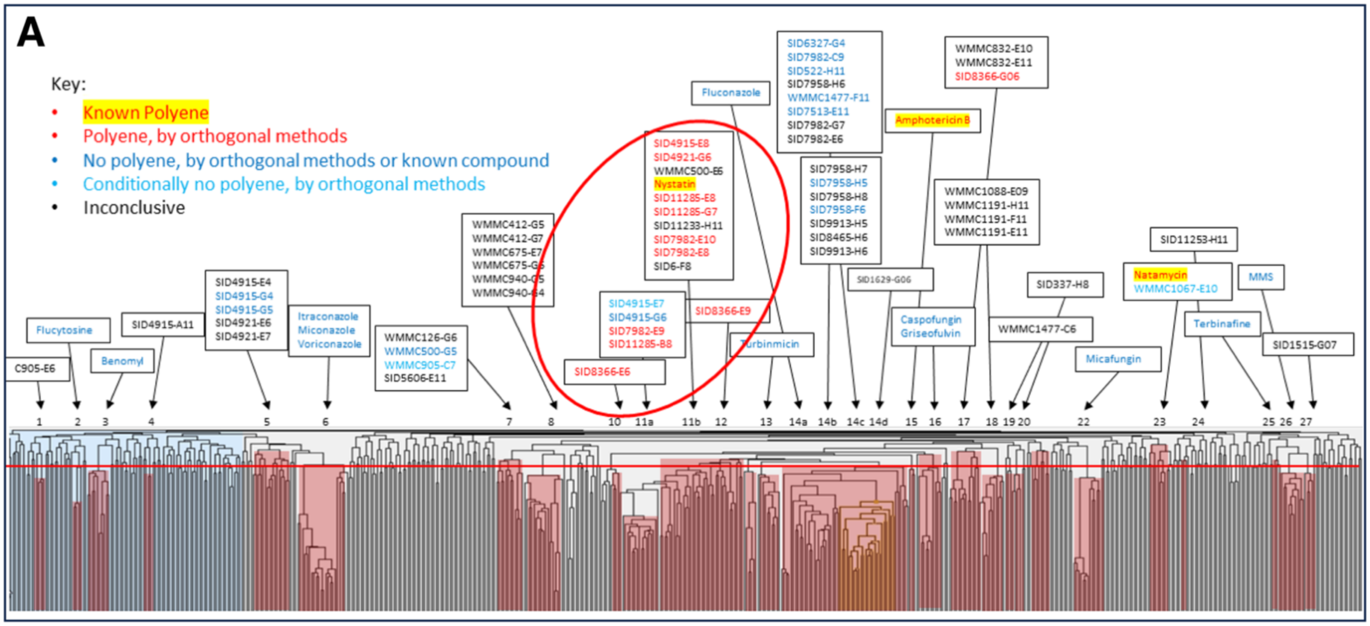

B. Polyene Antifungal Dereplication in Complex Bacterial Extract Fractions.

SID8366-E6: These are the results of dereplication using GNPS and SIRIUS 5 finding polyene macrolide compounds within the metabolomics analysis of the SID8366-E6 complex bacterial extract. Additionally presented is the MS2 spectra of the observed compound within the LC-MS/MS.

GNPS Results: No results for polyene macrolides.

SIRIUS Results:

Formula	Adduct and Mass	SMILES	CSI:FingerID Score	CANOPUS Class	PubChem Match ID
$C_{33}H_{54}O_{11}$	[M-H_{20}+H]+, 609.3621 Da	CCCCCC(C1C(CC(CC(CC(CC(C(C(C(=CC=CC=CC=CC=CC(C(OC1=O)C)O)C)O)O)O)O)O)O)O)O	-99.779	Macrolides and analogues; Macrolide lactones	9917196
$C_{33}H_{54}O_{11}$	[M+Na]+, 649.3543 Da	CCCC(C1C(CC(CC(CC(CC(CC(C(C(=CC=CC=CC=CC=CC(C(OC1=O)C)O)C)O)O)O)O)O)O)O)O	-147.678	Macrolides and analogues; Macrolide lactones	139589268

HR-LC-MS(MS) Spectra:

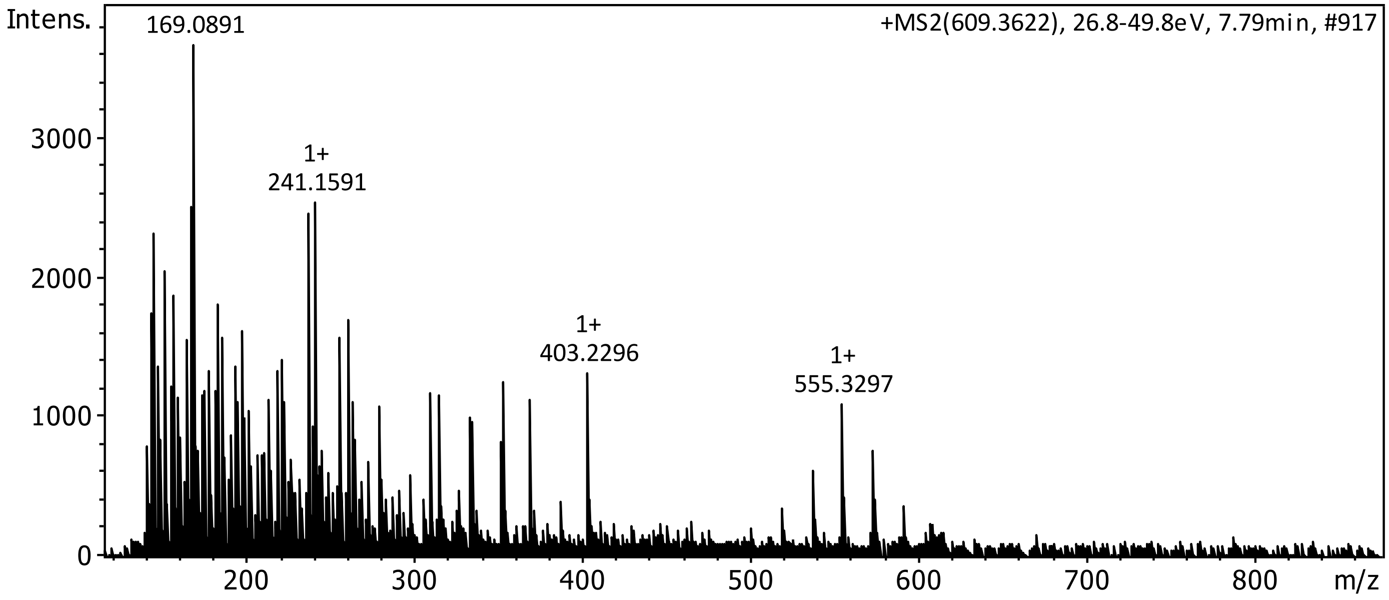
Intens.
3000
2000
1000
0
200
300
400
500
600
700
800
m/z
+MS2(609.3622), 26.8-49.8eV, 7.79min, #917
169.0891
1+
241.1591
1+
403.2296
1+
555.3297

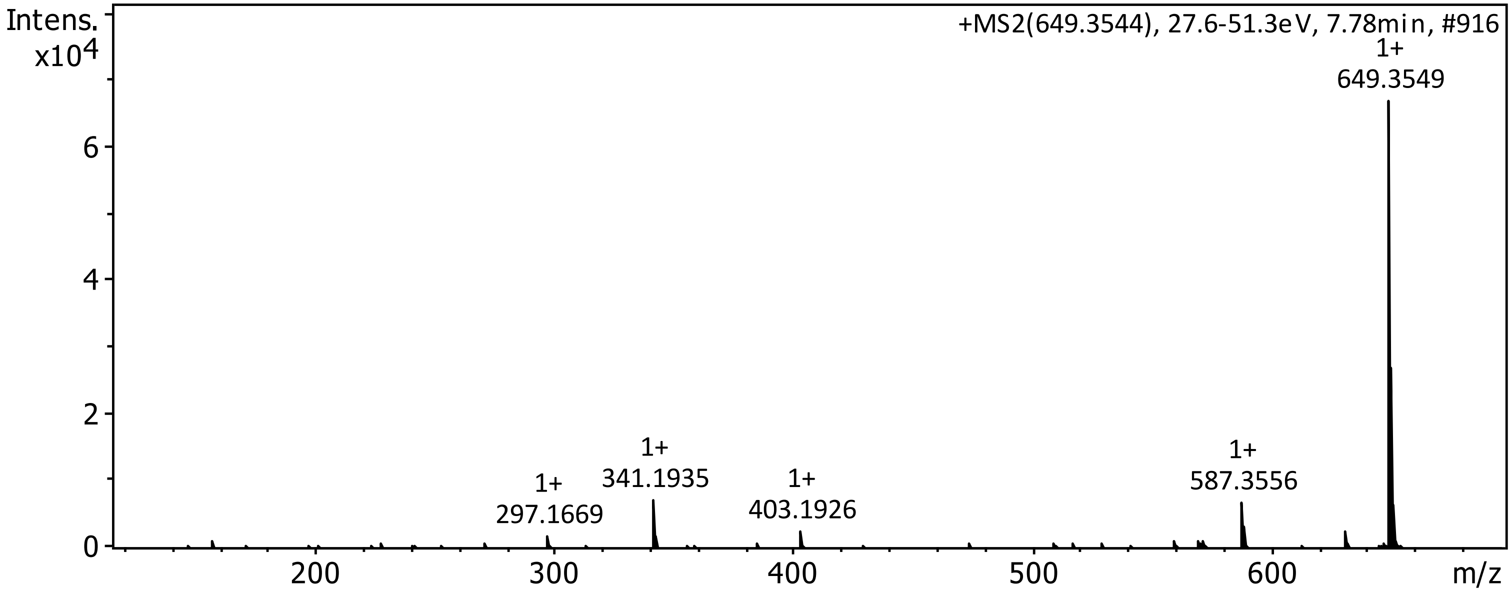
Intens.
x10^4
6
4
2
0
200
300
400
500
600
m/z
+MS2(649.3544), 27.6-51.3eV, 7.78min, #916
1+
649.3549
1+
297.1669
1+
341.1935
1+
403.1926
1+
587.3556

SID4921-G6: These are the results of dereplication using GNPS and SIRIUS 5 finding polyene macrolide compounds within the metabolomics analysis of the SID4921-G6 complex bacterial extract. Additionally presented is the MS2 spectra of the observed compound within the LC-MS/MS.

GNPS Results: No results for polyene macrolides.

SIRIUS Results:

Formula	Adduct and Mass	SMILES	CSI:FingerID Score	CANOPUS Class	PubChem Match ID
$C_{35}H_{58}O_{13}$	$[M + H]^+$, 687.3942 Da	CCCCCC(C1C(CC(CC(CC(CC(CC(C(C(C2(C(O2)C=CC=CC=CC=CC(C(OC1=O)C)O)C)O)O)O)O)O)O)O)O)O	-157.851	Monosaccharides; Macrolides	139589356
$C_{35}H_{58}O_{13}$	$[M + Na]^+$, 709.3759 Da	CCCCCC(C1C(CC(CC(CC(CC(CC(C(C(C2(C(O2)C=CC=CC=CC=CC(C(OC1=O)C)O)C)O)O)O)O)O)O)O)O)O	-199.529	Macrolides and analogues; Macrolide lactones	139589356

HR-LC-MS(MS) Spectra:

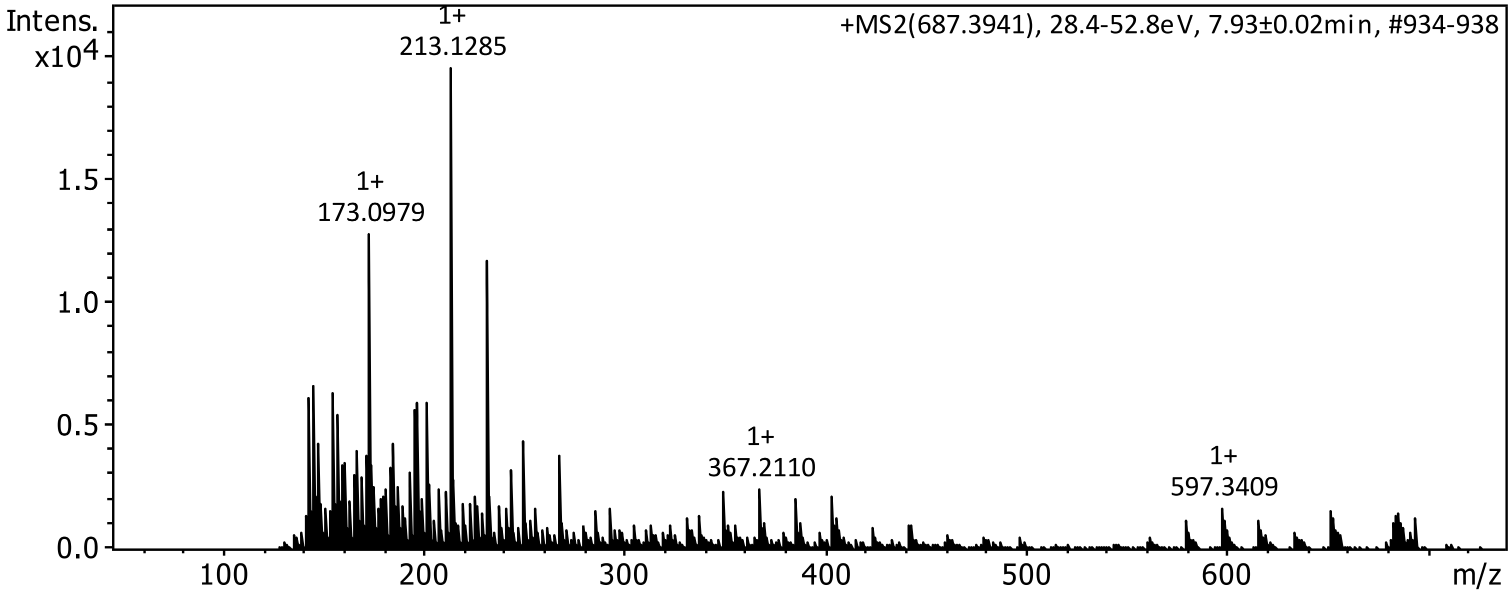
Intens.
x10^4
+MS2(687.3941), 28.4-52.8eV, 7.93±0.02min, #934-938
1+
213.1285
1+
173.0979
1+
367.2110
1+
597.3409
0.0
0.5
1.0
1.5
100
200
300
400
500
600
m/z

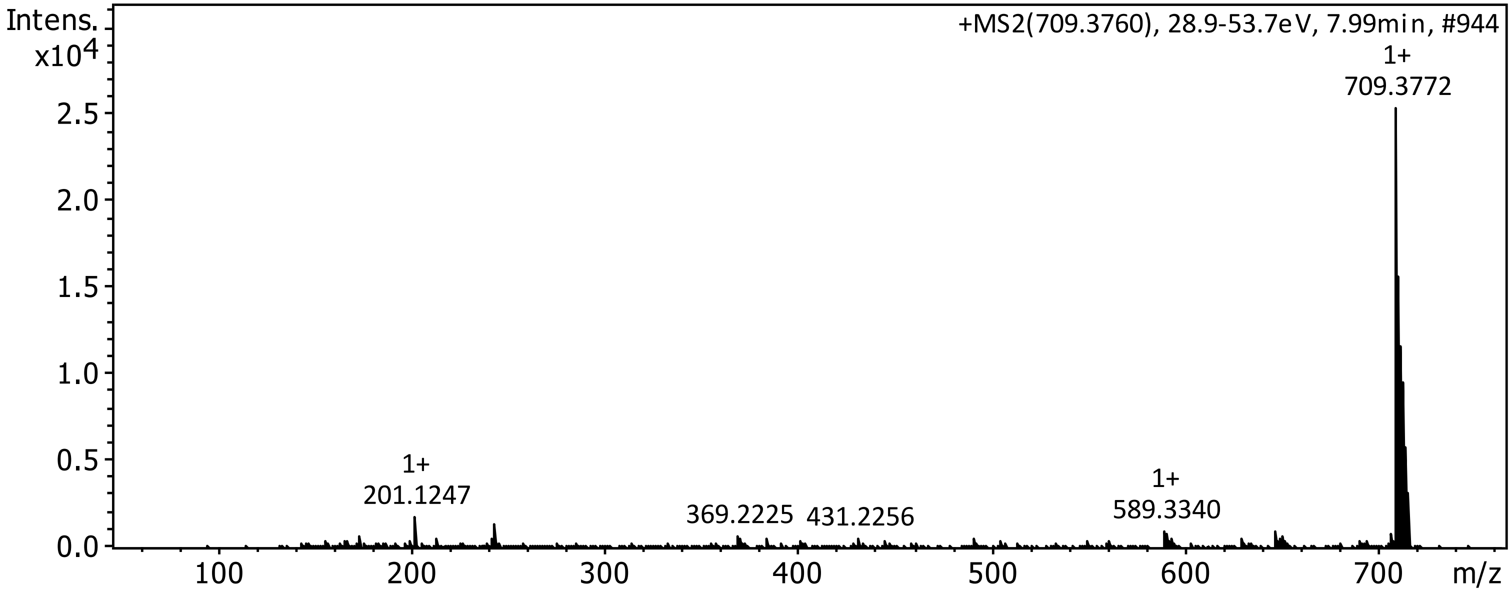
Intens.
x10^4
+MS2(709.3760), 28.9-53.7eV, 7.99min, #944
1+
709.3772
1+
201.1247
369.2225
431.2256
1+
589.3340
0.0
0.5
1.0
1.5
2.0
2.5
100
200
300
400
500
600
700
m/z

SID11285-E8: These are the results of dereplication using GNPS and SIRIUS 5 finding polyene macrolide compounds within the metabolomics analysis of the SID11285-E8 complex bacterial extract. Additionally presented is the MS2 spectra of the observed compound within the LC-MS/MS.

GNPS Results: No results for polyene macrolides.

SIRIUS Results:

Formula	Adduct and Mass	SMILES	CSI:FingerID Score	CANOPUS Class	PubChem Match ID
$C_{35}H_{53}NO_{13}$	$[M + H]^+$, 696.3574 Da	CCC1C=CC=CC=CC=CC(CC2C(C(CC(O2)(CC(CC(CC=CC(=O)OC1C)O)O)O)O)C(=O)O)OC3C(C(C(C(O3)C)O)N)O	-333.659	Diterpene glycoside; Polyketide	6441326
$C_{33}H_{49}NO_{11}$	$[M + Na]^+$, 658.3181 Da	CC1CC=CC=CC=CC=CC(CC2C(C(CC(O2)(CC(CC3C(O3)C=CC(=O)O1)O)O)O)C)OC4C(C(C(C(O4)C)O)N)O	-325.622	Amino acids and derivatives; Polyketides	122403081

HR-LC-MS(MS) Spectra:

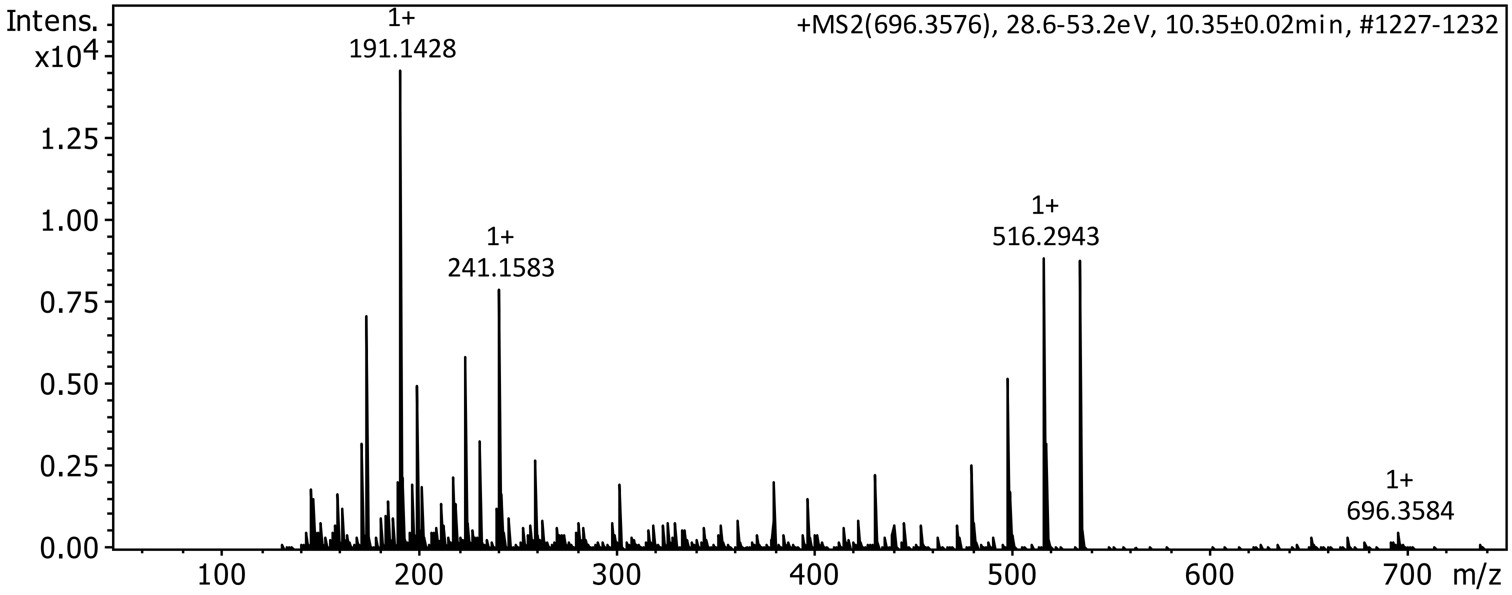
Intens.
x10^4
1.25
1.00
0.75
0.50
0.25
0.00
+MS2(696.3576), 28.6-53.2eV, 10.35±0.02min, #1227-1232
1+
191.1428
1+
241.1583
1+
516.2943
1+
696.3584
100
200
300
400
500
600
700
m/z

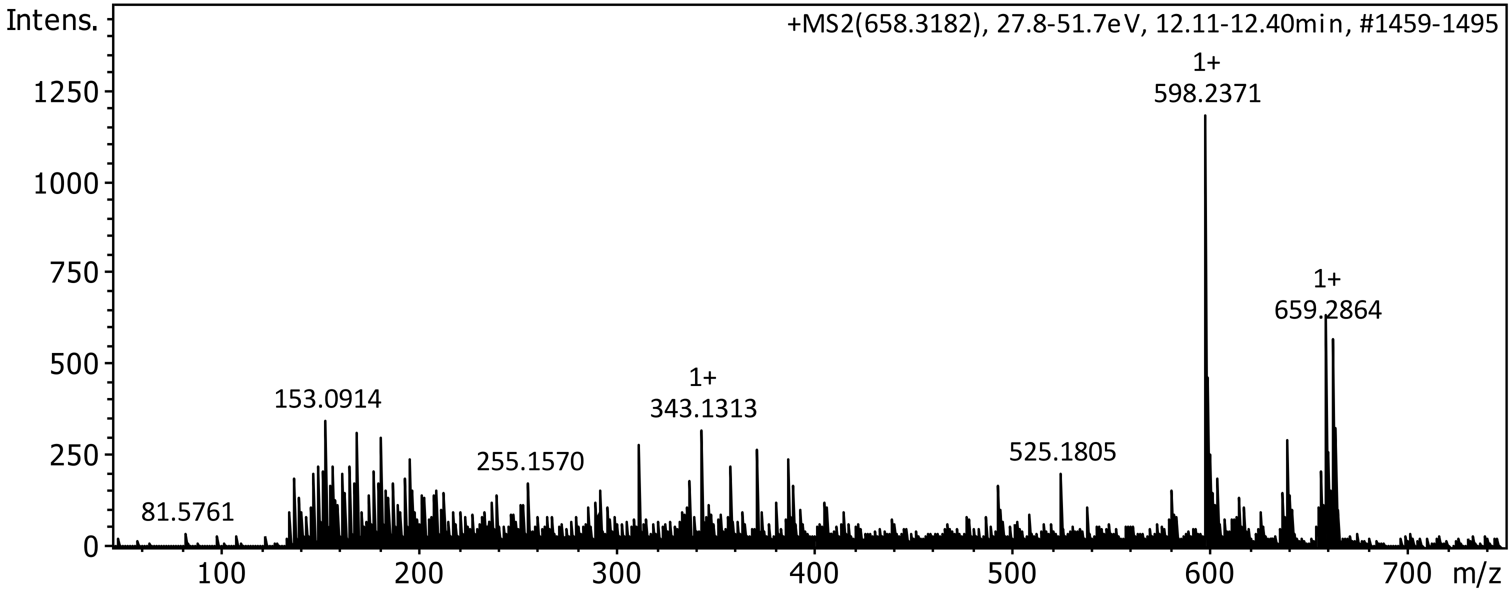
Intens.
1250
1000
750
500
250
0
+MS2(658.3182), 27.8-51.7eV, 12.11-12.40min, #1459-1495
1+
598.2371
1+
659.2864
153.0914
1+
343.1313
255.1570
525.1805
81.5761
100
200
300
400
500
600
700
m/z

SID7982-E8: These are the results of dereplication using GNPS and SIRIUS 5 finding polyene macrolide compounds within the metabolomics analysis of the SID7982-E8 complex bacterial extract. Additionally presented is the MS2 spectra of the observed compound within the LC-MS/MS.

GNPS Results: No results for polyene macrolides.

SIRIUS Results:

Formula	Adduct and Mass	SMILES	CSI:FingerID Score	CANOPUS Class	PubChem Match ID
$C_{48}H_{73}NO_{15}$	$[M + K]^{+}$, 942.4595 Da	CC1CC(=O)CC(CC(CCC(C(CC(CC2(CC(C(C(O2)CC(C=CC=CC=CC=CC=CC=CC=CC(C(C1C)O)C)OC3CC=C(C(C3O)N)O)C(=O)O)O)O)O)O)O)O)O	-247.342	Aminoglycosides; Macrolides	89333555

HR-LC-MS(MS) Spectra:

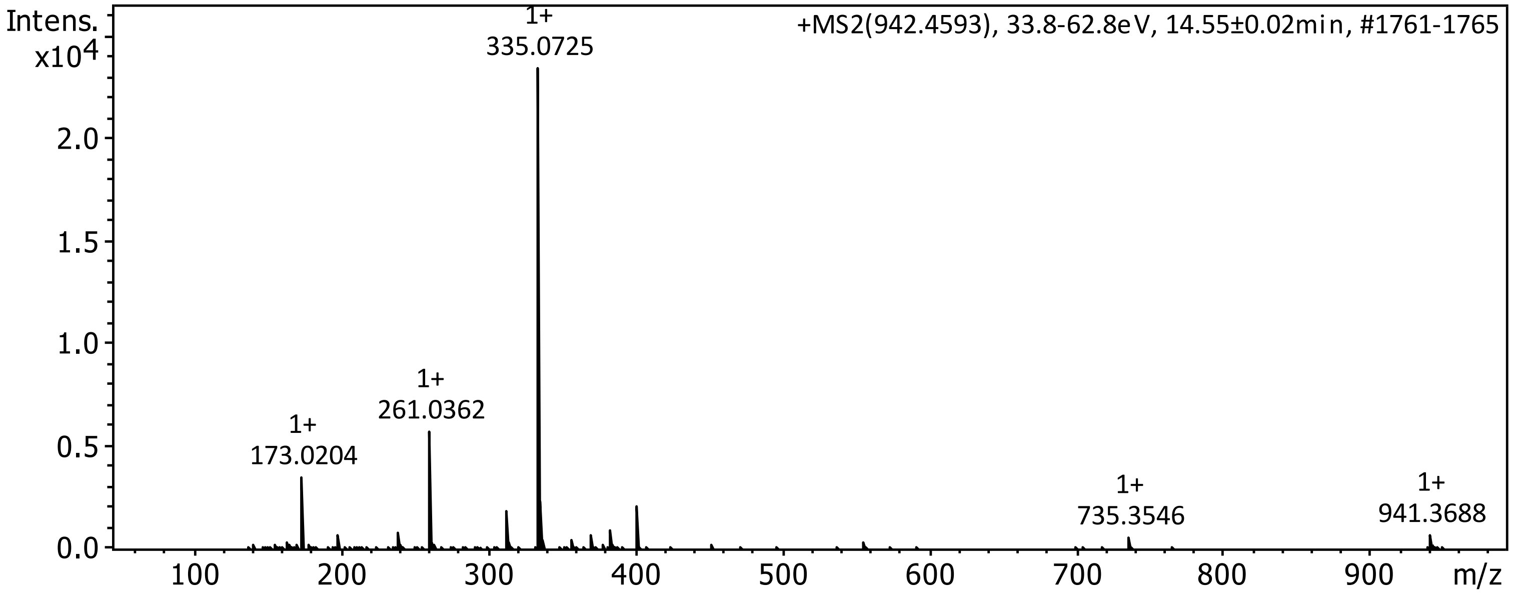

WMMC500-E6: These are the results of dereplication using GNPS and SIRIUS 5 finding polyene macrolide compounds within the metabolomics analysis of the WMMC500-E6 complex bacterial extract.

GNPS Results: No results for polyene macrolides.

SIRIUS Results: No polyene macrolide identifications.

HR-LC-MS(MS) Spectra: No polyene macrolide spectra.

Dataset S1 for Known Antifungals:

All datasets below (S1.1 – S.1.7) are composed of LC-HR-MS/MS metabolomics data and SIRIUS 5 outcomes for the identification of each of seven known antifungals within a complex bacterial extract.

Dataset S1.1: Amphotericin B: These are the results of dereplication using SIRIUS 5 finding amphotericin B within the metabolomics analysis when spiked into a complex bacterial extract. Additionally presented is the MS1 and MS2 spectra of the observed compound within the LC-MS/MS.

SIRIUS Dereplication Results:

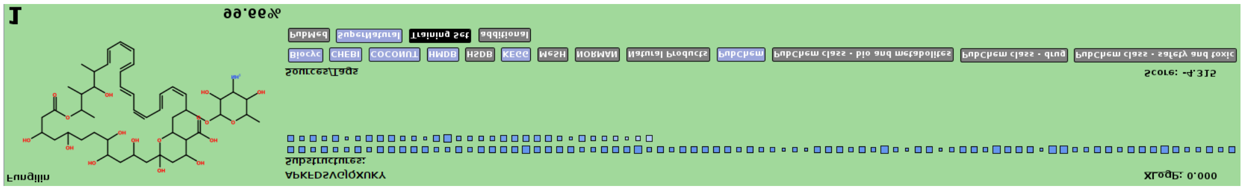

Rank	Molecular Formula	Adduct	Zodiac Score	Sirius Score	Isotope Score	Tree Score	Explained Peaks	Total Explained Intensity
1	C42H73N3O19	[M + H]+	N/A	99.994%	8.688	73.033	38/58	75.620%
2	C44H75O20	[M + H]+	N/A	0.006%	9.215	62.747	29/58	58.559%
3	C54H69NO12	[M + H]+	N/A	0.000%	6.029	63.409	36/58	75.424%
4	C47H73NO17	[M + H]+	N/A	0.000%	8.700	58.218	34/58	68.729%
5	C39H75N2O22	[M + H]+	N/A	0.000%	6.503	57.878	38/58	75.620%
6	C49H69N3O14	[M + H]+	N/A	0.000%	6.833	56.560	37/58	71.241%
7	C57H67N2O9	[M + H]+	N/A	0.000%	4.038	57.117	35/58	75.120%
8	C51H71O15	[M + H]+	N/A	0.000%	6.809	54.268	34/58	68.729%
9	C53H69N3O11	[M + H]+	N/A	0.000%	4.520	53.088	39/58	78.210%
10	C50H71N2O14	[M + H]+	N/A	0.000%	7.162	49.803	38/58	75.620%

Main Classes

Level 6: Aminoglycosides ⇨ Level 5: Aminosaccharides ⇨ Subclass: Carbohydrates and carbohydrate conjugates ⇨ Class: Organooxygen compounds ⇨ Superclass: Organic oxygen compounds ⇨ Kingdom: Organic compounds

Description

This compound belongs to the class Aminoglycosides, which describes molecules or a portion of a molecule composed of amino-modified sugars.

Alternative Classes

alternative: Macrolides and analogues; alternative: Hexoses; alternative: O-glycosyl compounds; alternative: Beta hydroxy acids and derivatives; alternative: Dicarboxylic acids and derivatives; alternative: Oxanes; alternative: 1,2-aminoalcohols; alternative: Carboxylic acid esters; alternative: Hemiacetals; alternative: Lactones; alternative: Secondary alcohols; alternative: Amino acids; alternative: Acetals; alternative: Carboxylic acids; alternative: Polyols; alternative: Oxacyclic compounds; alternative: Carbonyl compounds; alternative: Monoalkylamines; alternative: Hydrocarbon derivatives; alternative: Organic oxides; alternative: Organopnictogen compounds

Natural Product Classes

class: Polyene macrolides; Superclass: Macrolides; Pathway: Polyketides

HR-LC-MS(MS) Spectra:

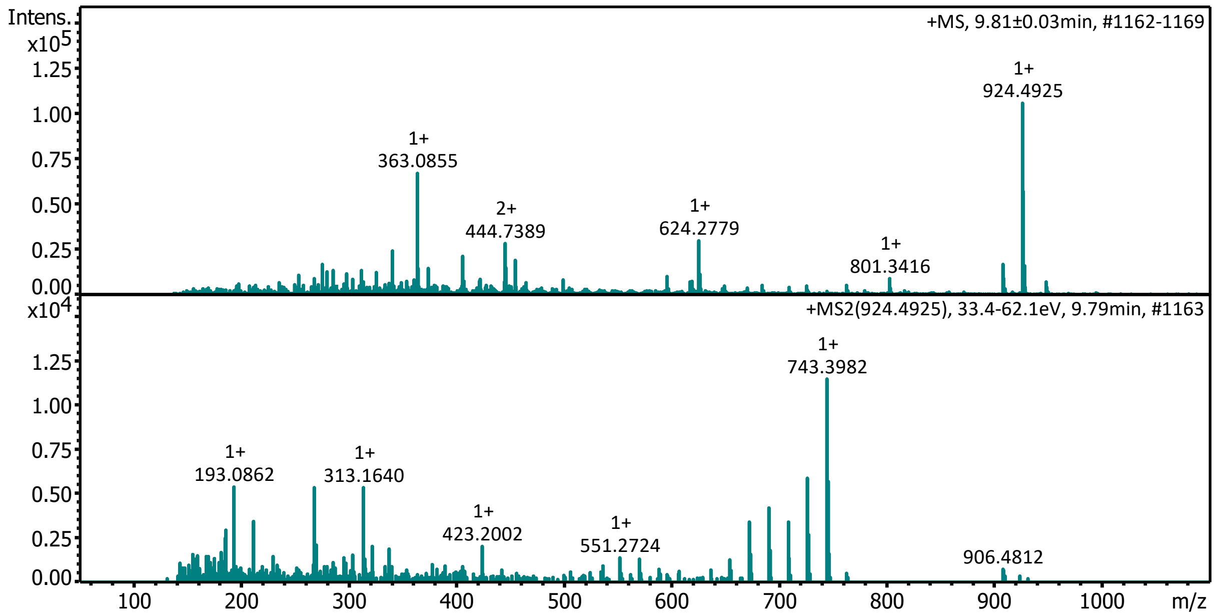

Dataset S1.2: Griseofulvin: These are the results of dereplication using SIRIUS 5 finding griseofulvin within the metabolomics analysis when spiked into a complex bacterial extract. Additionally presented is the MS1 and MS2 spectra of the observed compound within the LC-MS/MS.

SIRIUS Dereplication Results:

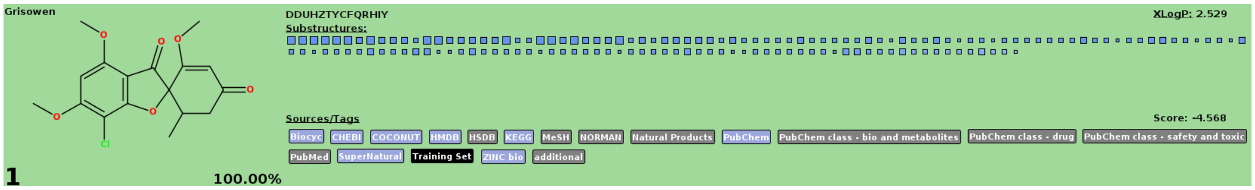

Rank	Molecular Formula	Adduct	Zodiac Score	Sirius Score	Isotope Score	Tree Score	Explained Peaks	Total Explained Intensity	Median Mass Error (pp...	Median Absolute Mass ...	Lipid Class
1	C17H17ClO6	[M + H]+	N/A	99.873%	6.879	45.114	26/58	78.434%	-1.676	2.351	None
2	C13H13ClN6O4	[M + H]+	N/A	0.107%	3.303	41.852	30/58	78.912%	4.613	4.613	None
3	C15H15ClN3O5	[M + H]+	N/A	0.018%	5.683	37.695	31/58	79.680%	2.727	2.866	None
4	C18H13ClN4O2	[M + H]+	N/A	0.002%	6.710	34.181	21/58	74.729%	-7.149	7.149	None
5	C18H12N2O6	[M + H]+	N/A	0.000%	2.529	37.177	20/58	71.503%	5.205	5.205	None
6	C10H17ClN6O4S	[M + H]+	N/A	0.000%	5.626	31.323	25/58	84.434%	-3.731	3.731	None
7	C21H10N3O3	[M + H]+	N/A	0.000%	2.846	32.012	20/58	71.503%	-5.310	5.310	None
8	C19H8N6O2	[M + H]+	N/A	0.000%	3.257	27.952	19/58	71.122%	-2.688	2.771	None
9	C12H19ClN3O5S	[M + H]+	N/A	0.000%	5.672	24.840	33/58	89.174%	-1.807	4.460	None
10	C14H21ClO6S	[M + H]+	N/A	0.000%	4.317	24.225	23/58	84.127%	-1.912	2.795	None

Main Classes

Class: Benzofurans ⇒ Superclass: Organoheterocyclic compounds ⇒ Kingdom: Organic compounds

Description

This compound belongs to the class Benzofurans, which describes organic compounds containing a benzene ring fused to a furan. Furan is a five-membered aromatic ring with four carbon atoms and one oxygen atom.

Alternative Classes

alternative: Coumarans | alternative: Anisoles | alternative: Aryl alkyl ketones | alternative: Alkyl aryl ethers | alternative: Cyclohexenones | alternative: Aryl chlorides | alternative: Vinylogous esters | alternative: Oxacyclic compounds | alternative: Organochlorides | alternative: Hydrocarbon derivatives | alternative: Organic oxides

Natural Product Classes

class: Griseofulvins | Superclass: Aromatic polyketides | Pathway: Polyketides

HR-LC-MS(MS) Spectra:

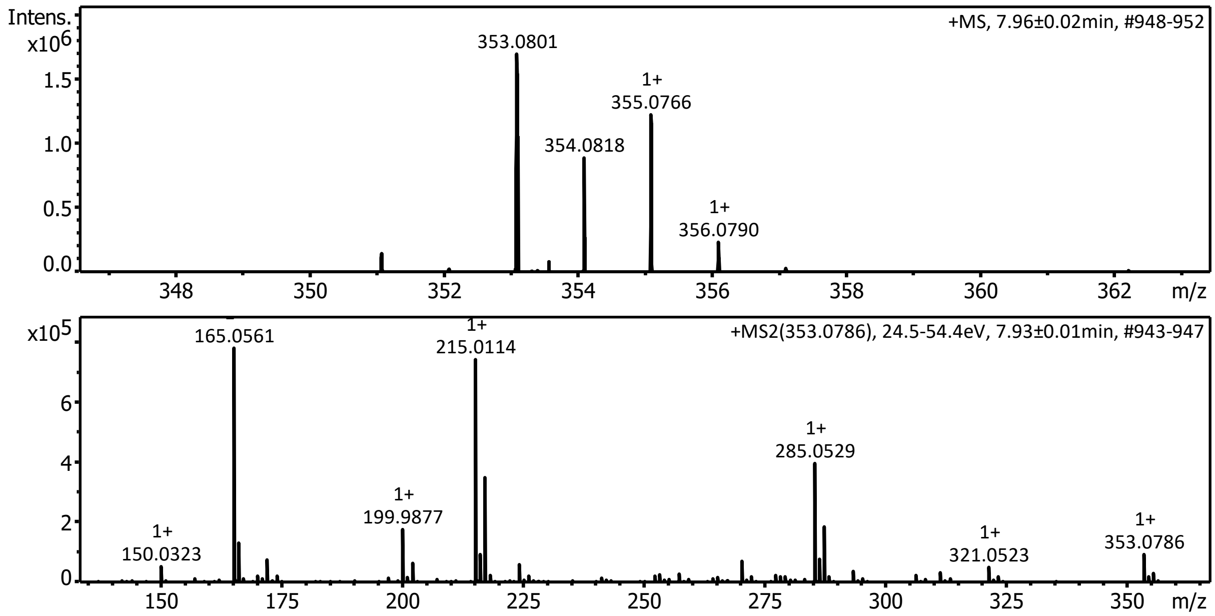

Dataset S1.3: Itraconazole: These are the results of dereplication using SIRIUS 5 finding itraconazole within the metabolomics analysis when spiked into a complex bacterial extract. Additionally presented is the MS1 and MS2 spectra of the observed compound within the LC-MS/MS.

SIRIUS Dereplication Results:

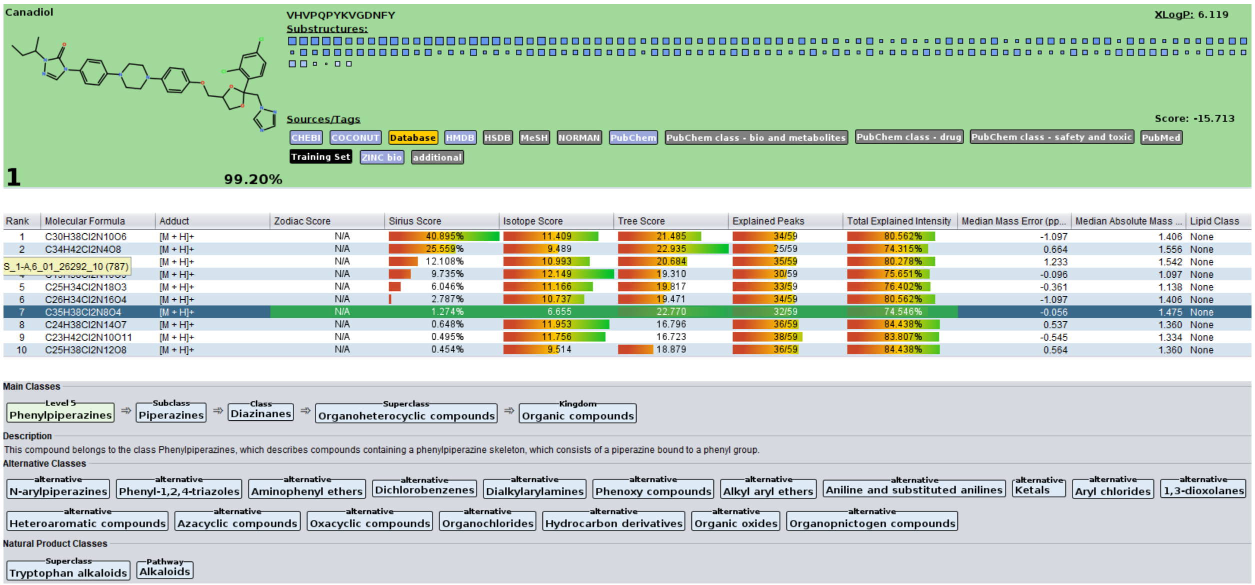

Rank	Molecular Formula	Adduct	Zodiac Score	Sirius Score	Isotope Score	Tree Score	Explained Peaks	Total Explained Intensity	Median Mass Error (pp...	Median Absolute Mass ...	Lipid Class
1	C30H38Cl2N10O6	[M + H]+	N/A	40.895%	11.409	21.485	34/59	80.562%	-1.097	1.406	None
2	C34H42Cl2N4O8	[M + H]+	N/A	25.559%	9.489	22.935	25/59	74.315%	0.664	1.556	None
[illegible]	[illegible]	[M + H]+	N/A	12.108%	10.993	20.684	35/59	80.278%	1.233	1.542	None
[illegible]	[illegible]	[M + H]+	N/A	9.735%	12.149	19.310	30/59	75.651%	-0.096	1.097	None
5	C25H34Cl2N18O3	[M + H]+	N/A	6.046%	11.166	19.817	33/59	76.402%	-0.361	1.138	None
6	C26H34Cl2N16O4	[M + H]+	N/A	2.787%	10.737	19.471	34/59	80.562%	-1.097	1.406	None
7	C35H38Cl2N8O4	[M + H]+	N/A	1.274%	6.655	22.770	32/59	74.546%	-0.056	1.475	None
8	C24H38Cl2N14O7	[M + H]+	N/A	0.648%	11.953	16.796	36/59	84.438%	0.537	1.360	None
9	C23H42Cl2N10O11	[M + H]+	N/A	0.495%	11.756	16.723	38/59	83.807%	-0.545	1.334	None
10	C25H38Cl2N12O8	[M + H]+	N/A	0.454%	9.514	18.879	36/59	84.438%	0.564	1.360	None

HR-LC-MS(MS) Spectra:

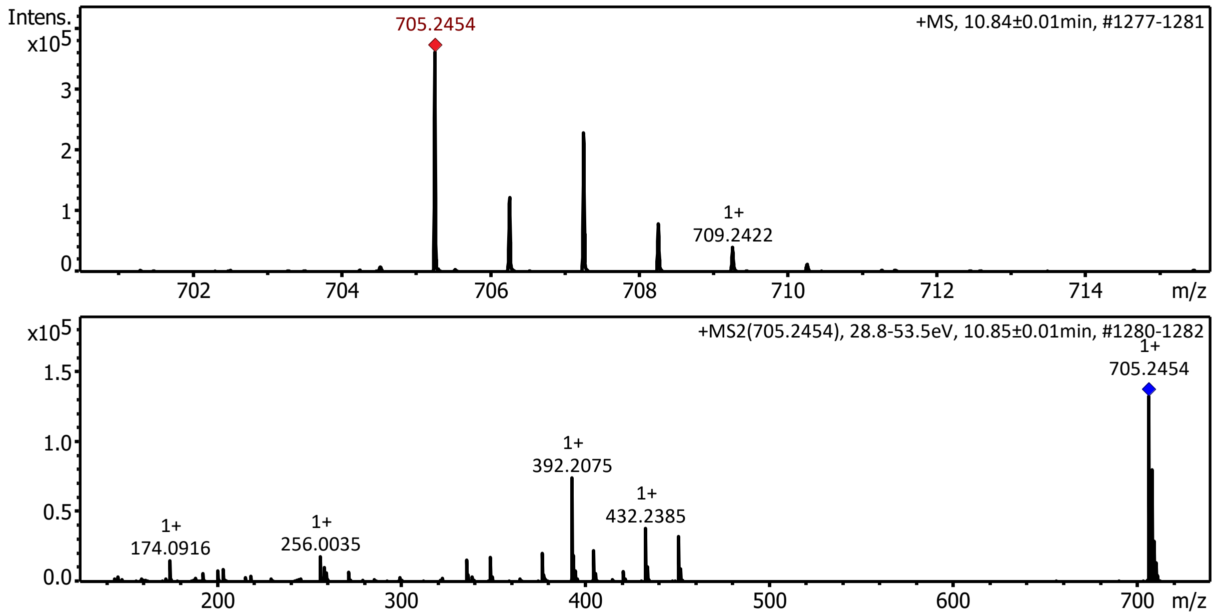

Dataset S1.4: Natamycin: These are the results of dereplication using SIRIUS 5 finding natamycin within the metabolomics analysis when spiked into a complex bacterial extract. Additionally presented is the MS1 and MS2 spectra of the observed compound within the LC-MS/MS.

SIRIUS Dereplication Results:

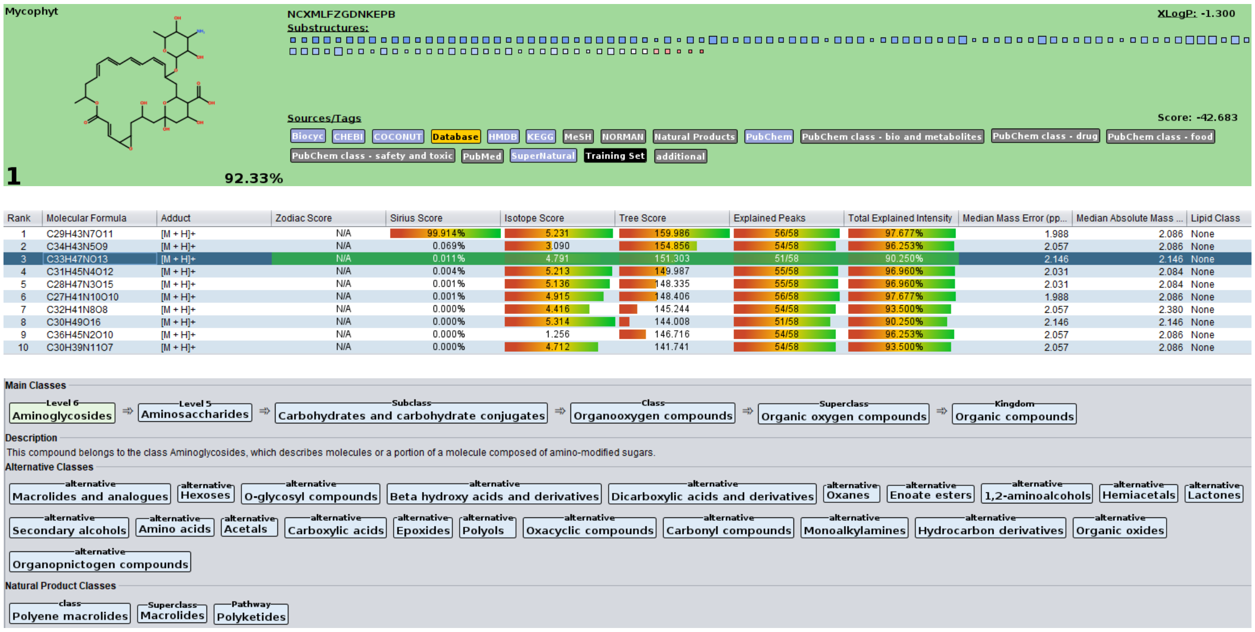

Rank	Molecular Formula	Adduct	Zodiac Score	Sirius Score	Isotope Score	Tree Score	Explained Peaks	Total Explained Intensity	Median Mass Error (pp...	Median Absolute Mass ...	Lipid Class
1	C29H43N7O11	[M + H]+	N/A	99.914%	5.231	159.986	56/58	97.677%	1.988	2.086	None
2	C34H43N5O9	[M + H]+	N/A	0.069%	3.090	154.856	54/58	96.253%	2.057	2.086	None
3	C33H47NO13	[M + H]+	N/A	0.011%	4.791	151.303	51/58	90.250%	2.146	2.146	None
4	C31H45N4O12	[M + H]+	N/A	0.004%	5.213	149.987	55/58	96.960%	2.031	2.084	None
5	C28H47N3O15	[M + H]+	N/A	0.001%	5.136	148.335	55/58	96.960%	2.031	2.084	None
6	C27H41N10O10	[M + H]+	N/A	0.001%	4.915	148.406	56/58	97.677%	1.988	2.086	None
7	C32H41N8O8	[M + H]+	N/A	0.000%	4.416	145.244	54/58	93.500%	2.057	2.380	None
8	C30H49O16	[M + H]+	N/A	0.000%	5.314	144.008	51/58	90.250%	2.146	2.146	None
9	C36H45N2O10	[M + H]+	N/A	0.000%	1.256	146.716	54/58	96.253%	2.057	2.086	None
10	C30H39N11O7	[M + H]+	N/A	0.000%	4.712	141.741	54/58	93.500%	2.057	2.086	None

Main Classes

Level 6: Aminoglycosides ⇒ Level 5: Aminosaccharides ⇒ Subclass: Carbohydrates and carbohydrate conjugates ⇒ Class: Organooxygen compounds ⇒ Superclass: Organic oxygen compounds ⇒ Kingdom: Organic compounds

Description

This compound belongs to the class Aminoglycosides, which describes molecules or a portion of a molecule composed of amino-modified sugars.

Alternative Classes

alternative: Macrolides and analogues | alternative: Hexoses | alternative: O-glycosyl compounds | alternative: Beta hydroxy acids and derivatives | alternative: Dicarboxylic acids and derivatives | alternative: Oxanes | alternative: Enoate esters | alternative: 1,2-aminoalcohols | alternative: Hemiacetals | alternative: Lactones | alternative: Secondary alcohols | alternative: Amino acids | alternative: Acetals | alternative: Carboxylic acids | alternative: Epoxides | alternative: Polyols | alternative: Oxacyclic compounds | alternative: Carbonyl compounds | alternative: Monoalkylamines | alternative: Hydrocarbon derivatives | alternative: Organic oxides | alternative: Organopnictogen compounds

Natural Product Classes

class: Polyene macrolides | Superclass: Macrolides | Pathway: Polyketides

HR-LC-MS(MS) Spectra:

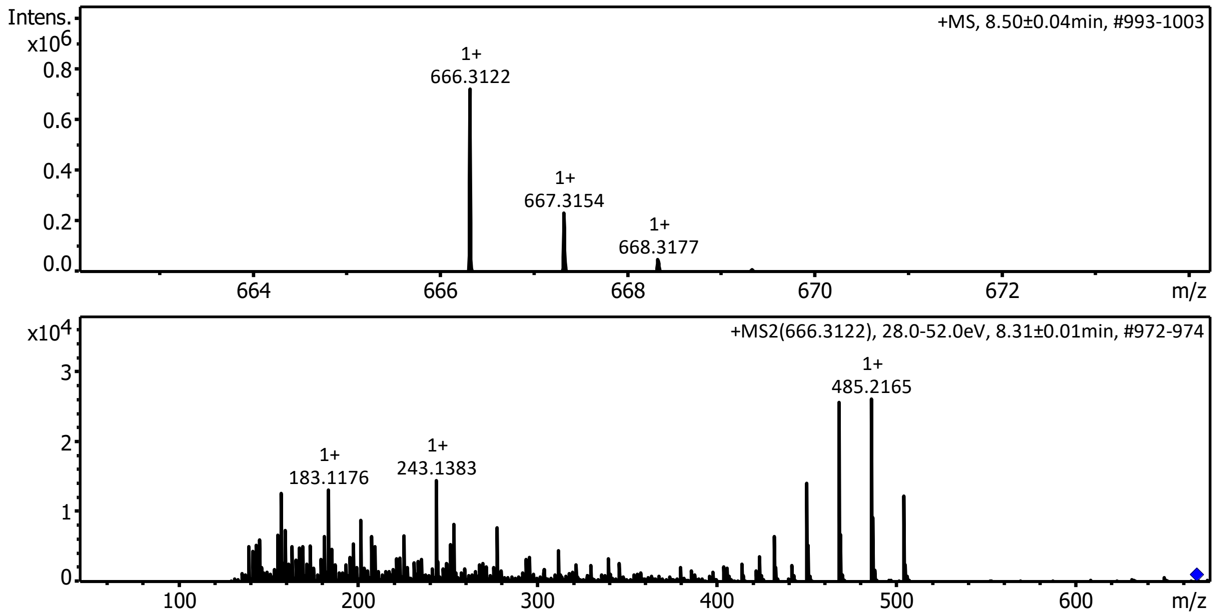

Dataset S1.5: Micafungin: These are the results of dereplication using SIRIUS 5 finding micafungin within the metabolomics analysis when spiked into a complex bacterial extract. Additionally presented is the MS1 spectra of the observed compound within the LC-MS/MS.

SIRIUS Dereplication Results:

SIRIUS was unable to identify the micafungin compound in the mass spectrometry data because the existing ions of micafungin were doubly charged, which SIRIUS cannot utilize. The parent ion, $[M+H]^+$, 1270.4458 is present but is dominated by the doubly charged, $[M+2H]^{2+}$, 595.7470. The accuracy of the LC-MS/MS mass is 0.9 mDa or 0.3 ppm.

HR-LC-MS/MS Spectra:

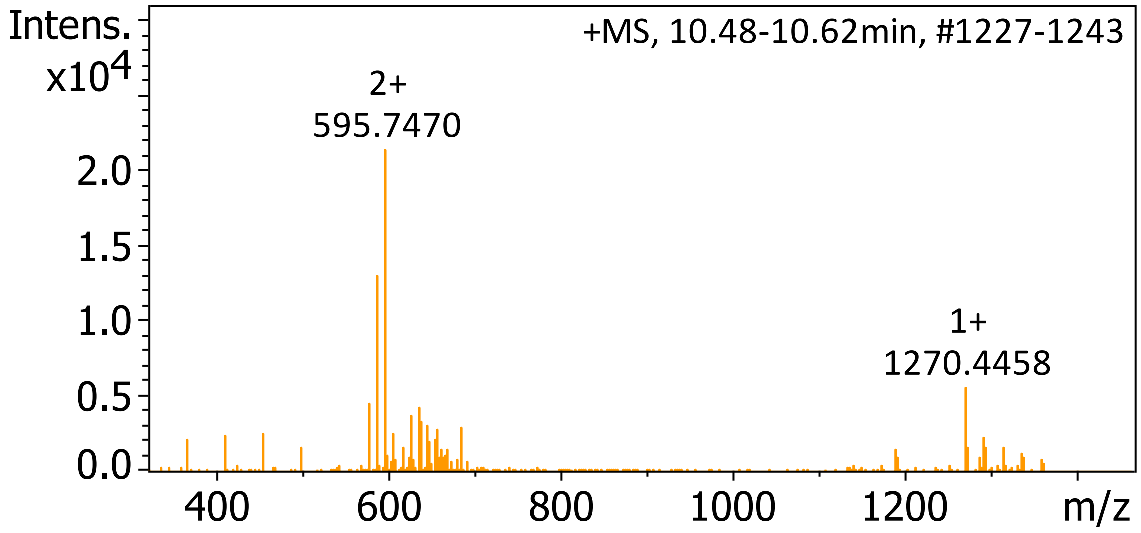

Dataset S1.6: Caspofungin: These are the results of dereplication using SIRIUS 5 finding caspofungin within the metabolomics analysis when spiked into a complex bacterial extract. Additionally presented is the MS1 and MS2 spectra of the observed compound within the LC-MS/MS.

SIRIUS Dereplication Results:

SIRIUS was not able to dereplicate caspofungin directly because the compound degraded either in solution or from in-source fragmentation. The losses from caspofungin equate to a loss of C_2H_8NO (62.0605 Da) from the original compound.

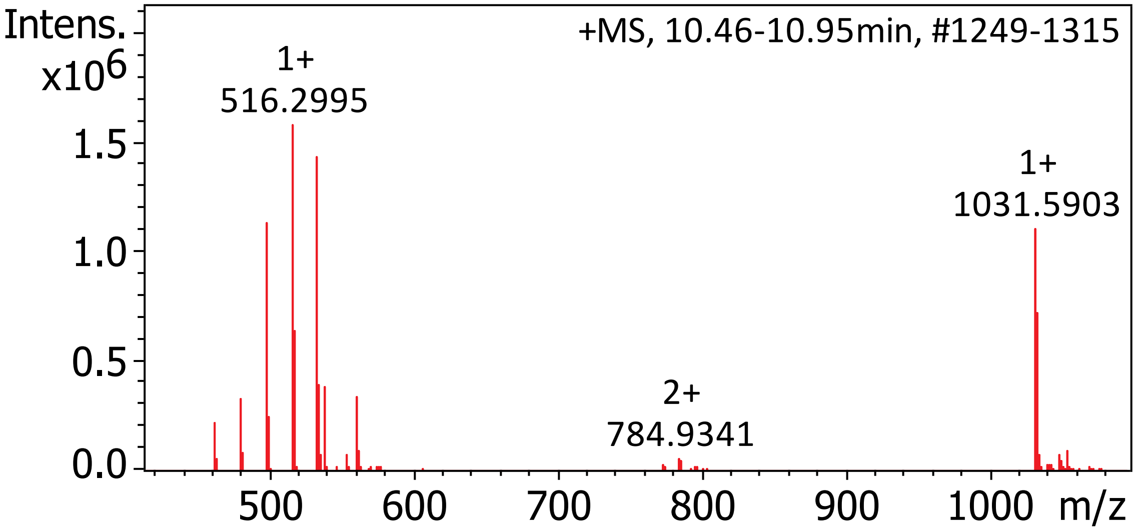

However, when the fragmentation spectra of 1031.5894 is paired with the parent mass of caspofungin, SIRIUS can identify it as caspofungin.

Caspofungin Degradation Product Identification:

Rank	Molecular Formula	Adduct	Zodiac Score	Sirius Score	Isotope Score	Tree Score	Explained Peaks	Total Explained Inte...	Median Mass Error (...	Median Absolute Ma...	Lipid Class
1	C57H88N8O13	[M + H]+	N/A	37.624%	0.000	38.224	26/40	94.406%	-0.582	0.943	None
2	C48H84N16O13	[M + H]+	N/A	15.112%	0.000	37.311	25/40	93.800%	1.044	1.066	None
3	C47H88N12O17	[M + H]+	N/A	11.200%	0.000	37.012	25/40	93.800%	1.066	1.286	None
4	C44H80N22O11	[M + H]+	N/A	7.297%	0.000	36.583	25/40	93.800%	1.066	1.286	None
5	C52H88N10O15	[M + H]+	N/A	7.186%	0.000	36.568	26/40	94.406%	-0.184	0.654	None
6	C51H92N6O19	[M + H]+	N/A	5.159%	0.000	36.237	26/40	94.406%	0.176	1.267	None
7	C43H84N18O15	[M + H]+	N/A	4.489%	0.000	36.097	25/40	93.800%	1.066	1.286	None
8	C36H88N18O20	[M + H]+	N/A	4.325%	0.000	36.060	25/40	93.800%	0.257	0.911	None
9	C42H84N20O14	[M + H]+	N/A	4.233%	0.000	36.039	26/40	94.406%	-0.582	0.943	None
10	C38H80N26O12	[M + H]+	N/A	3.374%	0.000	35.812	26/40	94.406%	-0.582	0.943	None

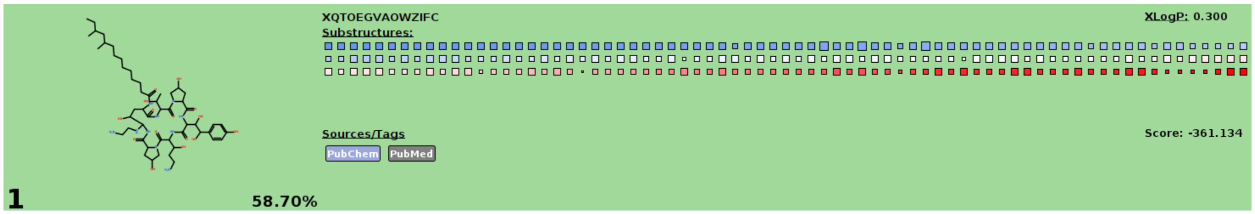

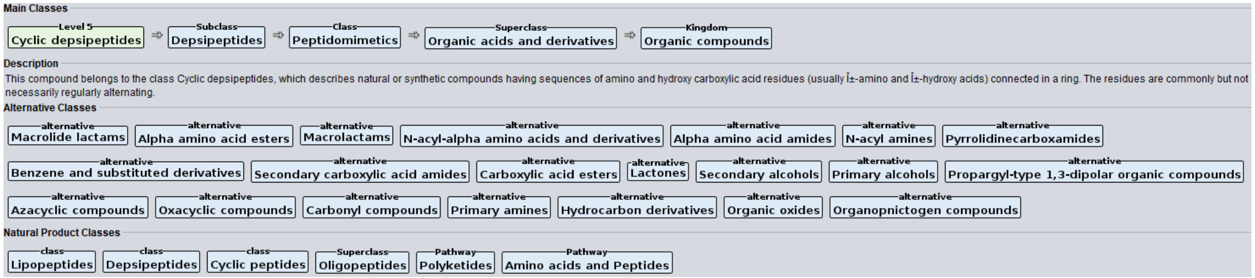

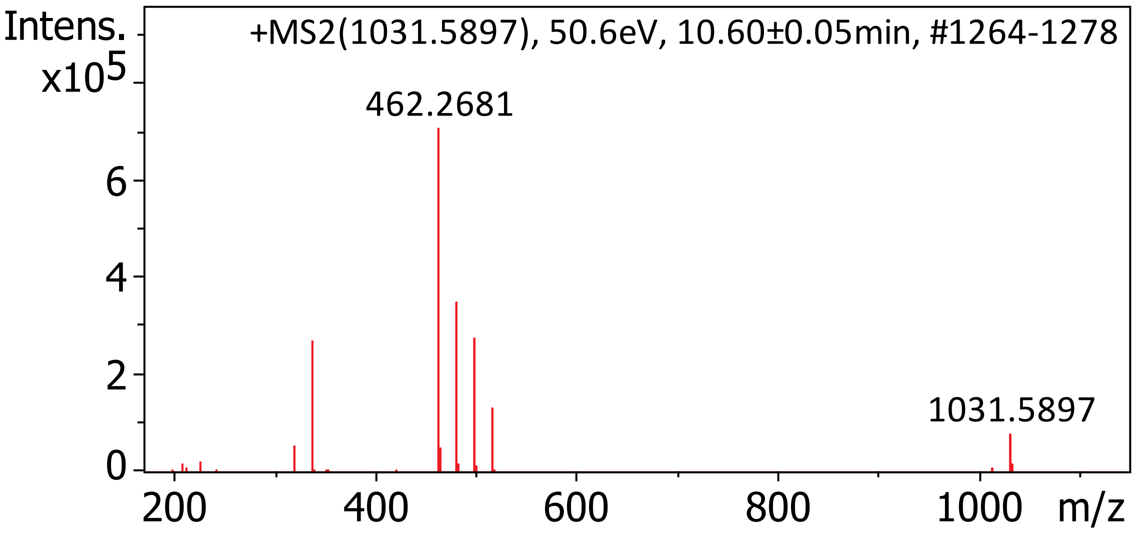

Dataset S1.7: Voriconazole: These are the results of dereplication using SIRIUS 5 finding voriconazole within the metabolomics analysis when spiked into a complex bacterial extract. Additionally presented is the MS1 and MS2 spectra of the observed compound within the LC-MS/MS.

SIRIUS Dereplication Results:

The parent ion of voriconazole can be seen in the LC-MS/MS data, in addition to the sodium and methanol adducts. However, the parent ion was not selected for fragmentation and SIRIUS was not able to identify voriconazole from the sodiated or methanolic adducts.

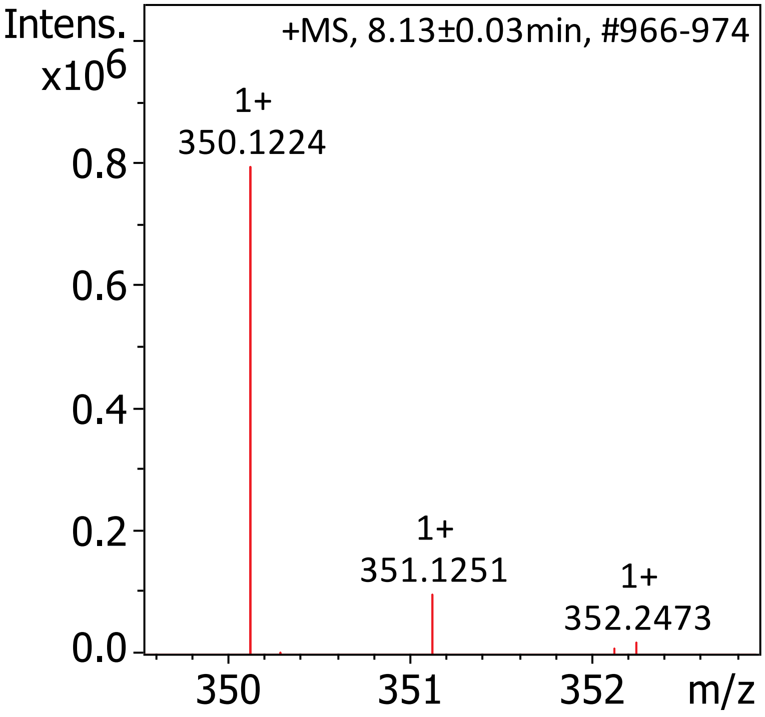

Sodium Adduct $[M+Na]^+$;

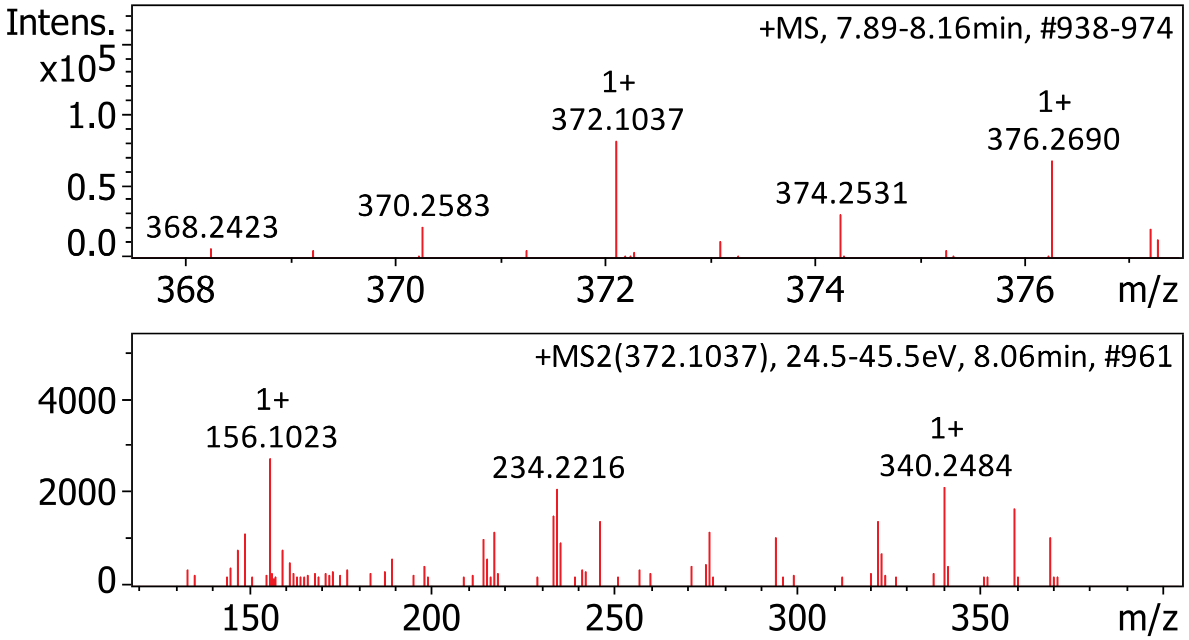

Methanol Adduct $[M+CH_4O]^+$;

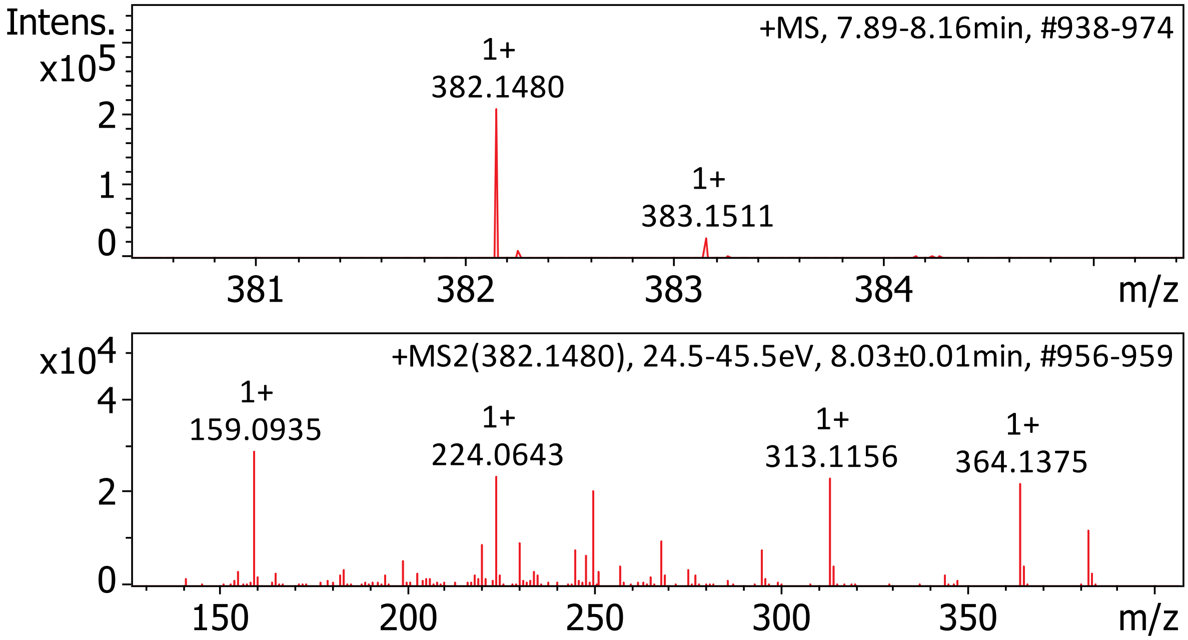

Dataset S2. Macrotetrolide Dereplication in Complex Bacterial Extract Fractions.

Dataset S2.1. SID7958-F6 Results: These data constitute dereplication of bacterial extract SID7958-F6 using SIRIUS 5, GNPS, and HR-LC-MS/MS. Presented are the GNPS spectra which match macrotetrolide spectra within the MSMS data of the extract, SIRIUS 5 macrotetrolide compound identifications with their associated mass, adduct, CSI Confidence Score, classification, and PubChem ID, and the MS1 and MS2 spectra associated with each identified macrotetrolide compound.

GNPS Match Results:

Adduct	Compound Name	MZ Error PPM	Spectrum ID	NPClassifier Class
$[M+NH_4]^+$	Monactin	4.36823	CCMSLIB00000851861	Macrotetrolides
$[M+NH_4]^+$	Monactin	3.09747	CCMSLIB00000851861	Macrotetrolides
$[M+NH_4]^+$	Monactin	5.79783	CCMSLIB00000851861	Macrotetrolides

SIRIUS Results:

Macrotetrolide Masses (Da)			Detected by SIRIUS	CSI Score	CANOPUS Class	PubChem ID Match
Monactin	751.4632	$[M+H]^+$	No	N/A	N/A	N/A
	768.489	$[M+NH_4]^+$	Yes	-4.796	Macrotetrolide	169015
	773.4467	$[M +Na]^+$	No	N/A	N/A	N/A
	789.419	$[M+K]^+$	No	N/A	N/A	N/A
Dinactin	765.4789	$[M+H]^+$	No	N/A	N/A	N/A
	782.5054	$[M+NH_4]^+$	Yes	-13.35	Macrotetrolide	13454270
	787.460	$[M+Na]^+$	No	N/A	N/A	N/A
	803.434	$[M+K]^+$	No	N/A	N/A	N/A
Trinactin	779.4945	$[M+H]^+$	No	N/A	N/A	N/A
	796.5211	$[M+NH_4]^+$	Yes	-8.99	Macrotetrolide	169021
	801.4765	$[M+Na]^+$	No	N/A	N/A	N/A
	817.4504	$[M+K]^+$	No	N/A	N/A	N/A
Tetranactin	793.5102	$[M+H]^+$	No	N/A	N/A	N/A
	810.5367	$[M+NH_4]^+$	No	N/A	N/A	N/A
	815.4921	$[M+Na]^+$	No	N/A	N/A	N/A
	831.4660	$[M+K]^+$	No	N/A	N/A	N/A
Nonactin	737.4476	$[M+H]^+$	No	N/A	N/A	N/A
	754.4741	$[M+NH_4]^+$	Yes	-19.83	Macrotetrolide	72519
	759.4295	$[M+Na]^+$	No	N/A	N/A	N/A
	775.4034	$[M+K]^+$	No	N/A	N/A	N/A

HR-LC-MS(MS) Spectra:

Monactin Spectra:

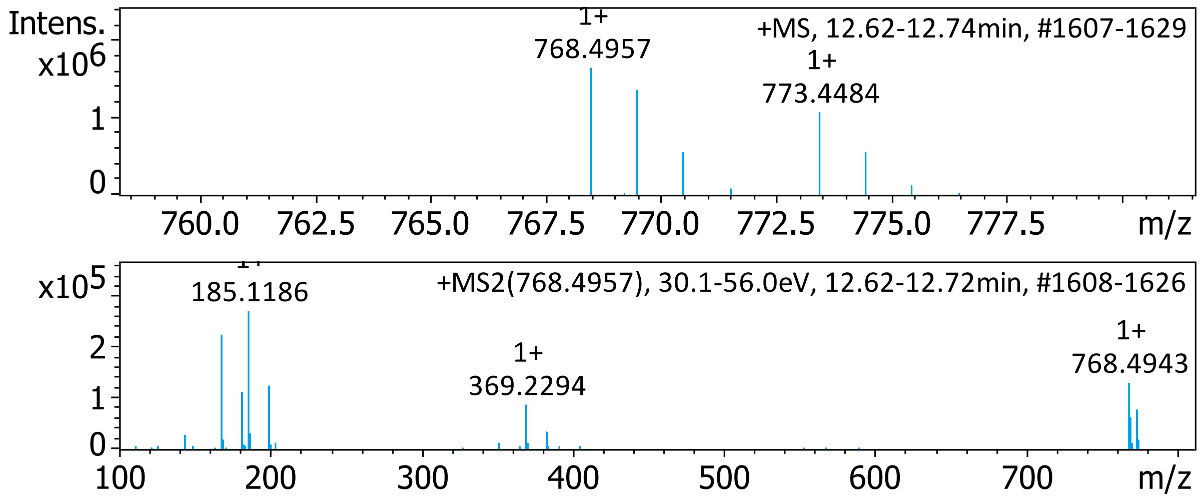

Dinactin Spectra:

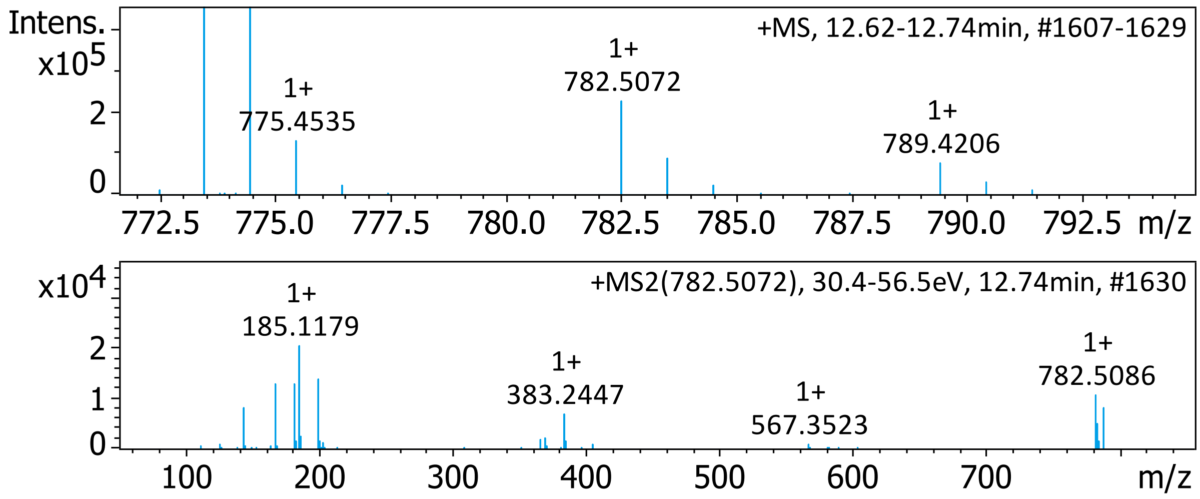

Trinactin Spectra:

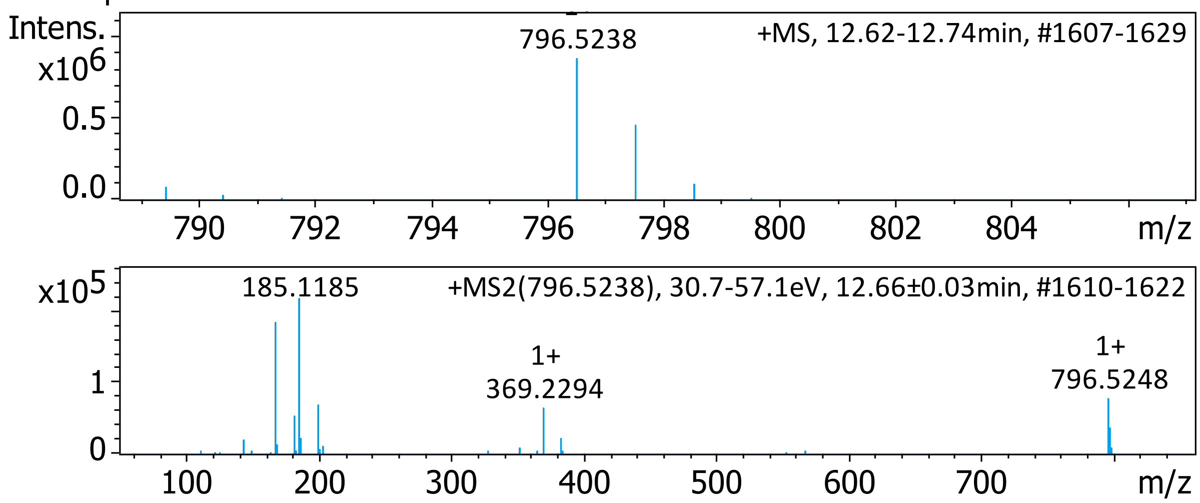

Nonactin Spectra:

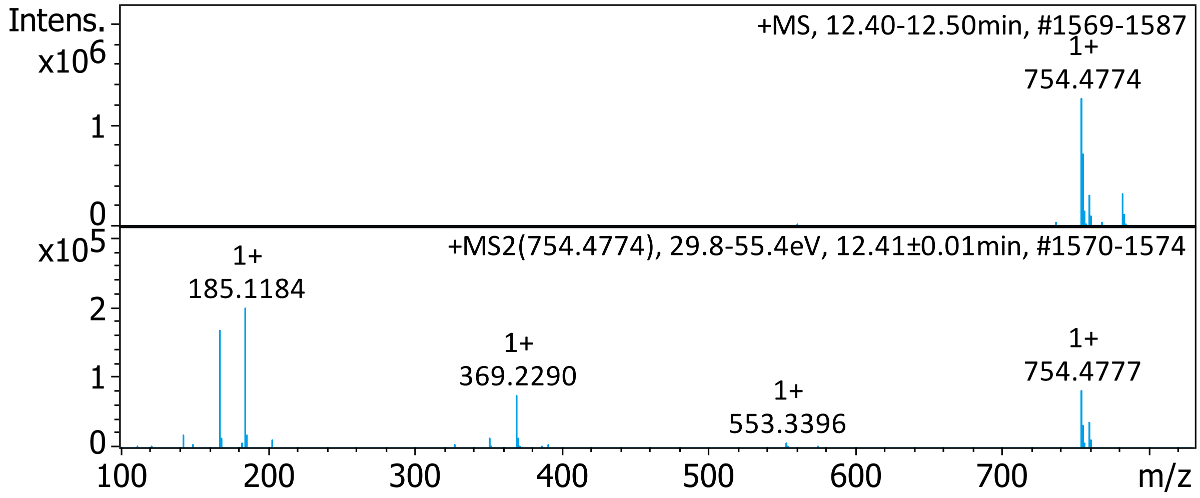

Dataset S2.2. SID7958-H8 Results: These data constitute the dereplication of bacterial extract SID7958-H8 using SIRIUS 5, GNPS, and HR-LC-MS/MS. Presented are the GNPS spectra which match macrotetrolide spectra within the MSMS data of the extract, SIRIUS 5 macrotetrolide compound identifications with their associated mass, adduct, CSI Confidence Score, classification, and PubChem ID, and the MS1 and MS2 spectra associated with each identified macrotetrolide compound.

GNPS Match Results:

Adduct	Compound Name	MZ Error PPM	Spectrum ID	NPClassifier Class
$[M+NH_4]^+$	Monactin	3.25631	CCMSLIB00000851861	Macrotetrolides
$[M+NH_4]^+$	Monactin	1.58845	CCMSLIB00000851861	Macrotetrolides

$[M+H]^+$	Monactin	0.243665	CCMSLIB00000851859	Macrotetrolides
$[M+H]^+$	Monactin	0.974661	CCMSLIB00000851859	Macrotetrolides
$[M+NH_4]^+$	Monactin	1.98556	CCMSLIB00000851861	Macrotetrolides
$[M+H]^+$	Nonactin	0.413827	CCMSLIB00010109074	Macrotetrolides
$[M+H]^+$	Nonactin	5.87635	CCMSLIB00010109074	Macrotetrolides

SIRIUS Results:

Macrotetrolide Masses			Detected by SIRIUS	CSI Score	CANOPUS Class	PubChem ID Match
Monactin	751.4632	$[M+H]^+$	Yes	-11.52	Macrotetrolide	169015
	768.489	$[M+NH_4]^+$	Yes	-8.71	Macrotetrolide	169015
	773.4467	$[M+Na]^+$	Yes	-7.47	Macrotetrolide	169015
	789.419	$[M+K]^+$	Yes	-12.65	Macrotetrolide	169015
Dinactin	765.4789	$[M+H]^+$	No	N/A	N/A	N/A
	782.5054	$[M+NH_4]^+$	Yes	-5.18	Macrotetrolide	6916048
	787.460	$[M+Na]^+$	No	N/A	N/A	N/A
	803.434	$[M+K]^+$	No	N/A	N/A	N/A
Trinactin	779.4945	$[M+H]^+$	No	N/A	N/A	N/A
	796.5211	$[M+NH_4]^+$	Yes	-11.37	Macrotetrolide	169021
	801.4765	$[M+Na]^+$	No	N/A	N/A	N/A
	817.4504	$[M+K]^+$	No	N/A	N/A	N/A
Tetranactin	793.5102	$[M+H]^+$	No	N/A	N/A	N/A
	810.5367	$[M+NH_4]^+$	No	N/A	N/A	N/A
	815.4921	$[M+Na]^+$	No	N/A	N/A	N/A
	831.4660	$[M+K]^+$	No	N/A	N/A	N/A
Nonactin	737.4476	$[M+H]^+$	Yes	-35.47	Macrotetrolide	72519
	754.4741	$[M+NH_4]^+$	Yes	-23.94	Macrotetrolide	72519
	759.4295	$[M+Na]^+$	No	N/A	N/A	N/A
	775.4034	$[M+K]^+$	Yes	-40.62	Macrotetrolide	72519

HR-LC-MS(MS) Spectra

Monactin Spectra:

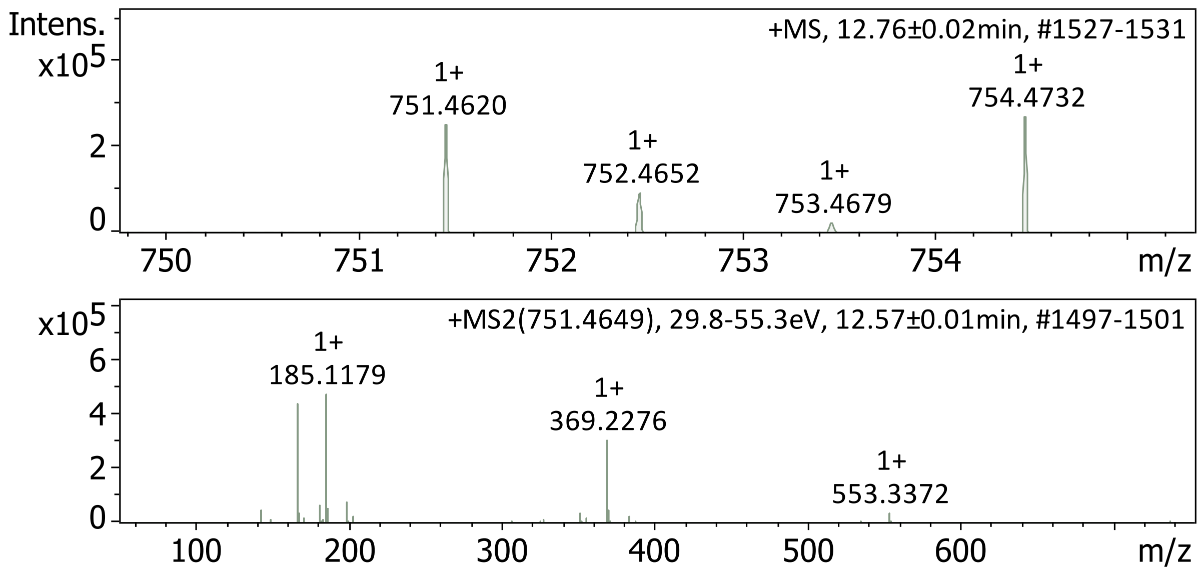

Dinactin Spectra:

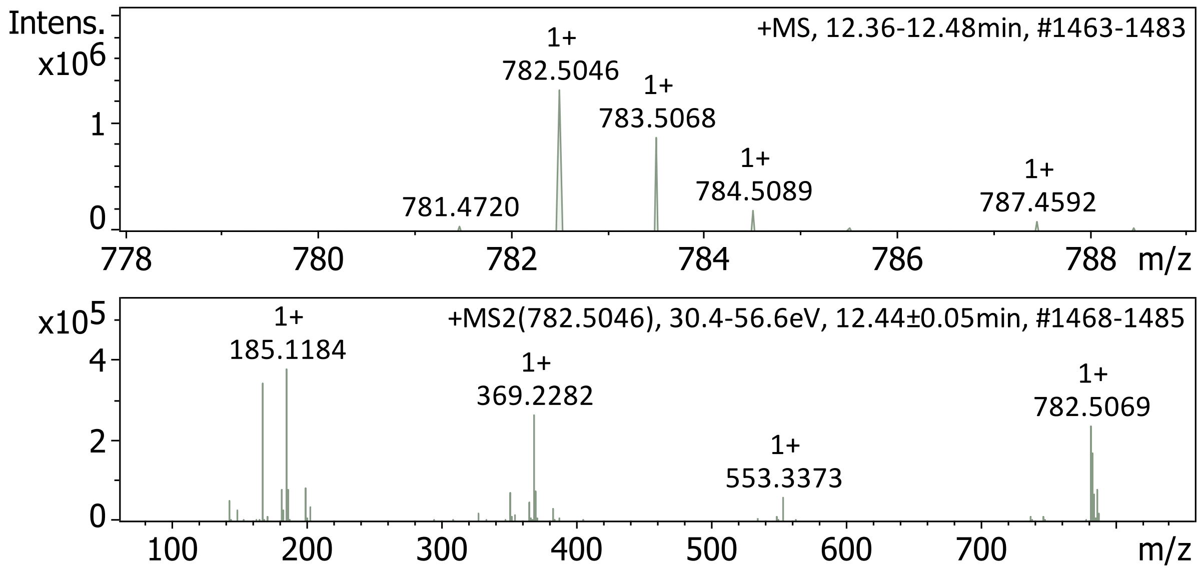

Trinactin Spectra:

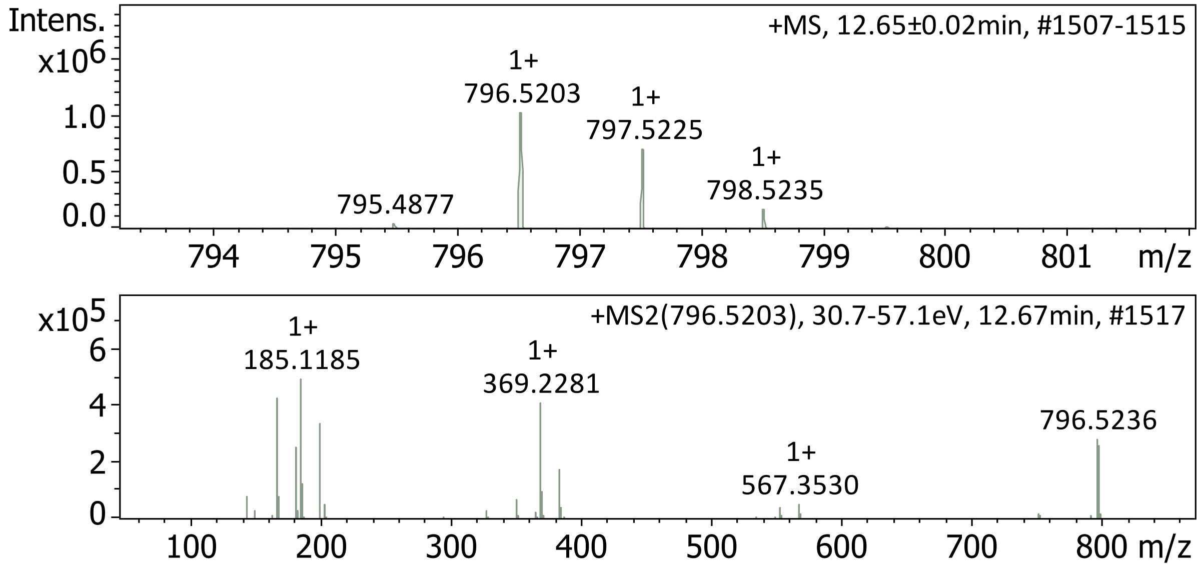

Nonactin Spectra:

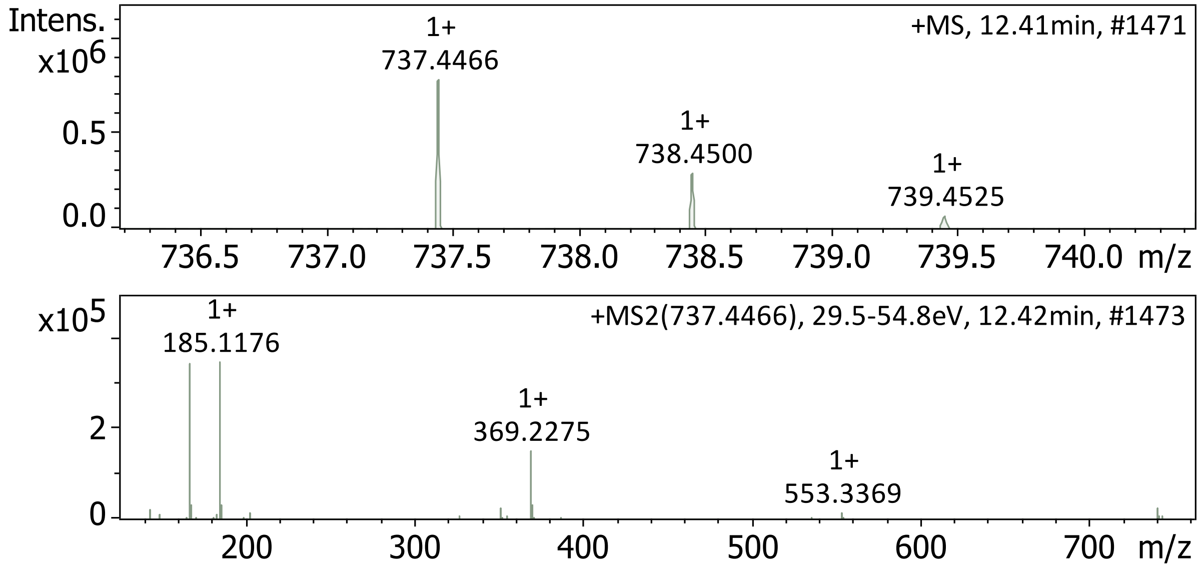

Dataset S2.3. SID9913-H5 Results: These data constitute dereplication of the bacterial extract SID9913-H5 using SIRIUS 5, GNPS, and HR-LC-MS/MS. Presented are the GNPS spectra which match macrotetrolide spectra within the MSMS data of the extract, SIRIUS 5 macrotetrolide compound identifications with their associated mass, adduct, CSI Confidence Score, classification, and PubChem ID, and the MS1 and MS2 spectra associated with each identified macrotetrolide compound.

GNPS Match Results:

Adduct	Compound Name	MZ Error PPM	Spectrum ID	NPClassifier Class
$[M+NH_4]^+$	Monactin	5.0036	CCMSLIB00000851861	Macrotetrolides
$[M+H]^+$	Monactin	2.27421	CCMSLIB00000851859	Macrotetrolides
$[M+H]^+$	Monactin	4.71086	CCMSLIB00000851859	Macrotetrolides
$[M+NH_4]^+$	Monactin	0.476534	CCMSLIB00000851861	Macrotetrolides
$[M+H]^+$	Dinactin	4.06647	CCMSLIB00000856076	Macrotetrolides
$[M+H]^+$	Dinactin	3.28864	CCMSLIB00000854224	Macrotetrolides
$[M+H]^+$	Nonactin	4.63487	CCMSLIB00010109074	Macrotetrolides
$[M+H]^+$	Trinactin	1.64432	CCMSLIB00000854224	Macrotetrolides
$[M+H]^+$	Dinactin	4.30567	CCMSLIB00000856076	Macrotetrolides
$[M+H]^+$	Nonactin	6.04188	CCMSLIB00010109074	Macrotetrolides

SIRIUS Results:

Macrotetrolide Masses			Detected by SIRIUS	CSI Score	CANOPUS Class	PubChem ID Match
Monactin	751.4632	$[M+H]^+$	Yes	-12.69	Macrotetrolide	169015
	768.489	$[M+NH_4]^+$	Yes	-7.98	Macrotetrolide	169015
	773.4467	$[M +Na]^+$	No	N/A	N/A	N/A
	789.419	$[M+K]^+$	Yes	-17.20	Macrotetrolide	169015
Dinactin	765.4789	$[M+H]^+$	Yes	-4.62	Macrotetrolide	6916048
	782.5054	$[M+NH_4]^+$	Yes	-6.426	Macrotetrolide	6916048
	787.460	$[M+Na]^+$	No	N/A	N/A	N/A
	803.434	$[M+K]^+$	Yes	-11.40	Macrotetrolide	6916048
Trinactin	779.4945	$[M+H]^+$	Yes	-5.20	Macrotetrolide	169021
	796.5211	$[M+NH_4]^+$	Yes	-4.80	Macrotetrolide	169021
	801.4765	$[M+Na]^+$	No	N/A	N/A	N/A
	817.4504	$[M+K]^+$	Yes	-10.03	Macrotetrolide	169021
Tetranactin	793.5102	$[M+H]^+$	Yes	-25.23	Macrotetrolide	441165
	810.5367	$[M+NH_4]^+$	Yes	-23.67	Macrotetrolide	441165
	815.4921	$[M+Na]^+$	Yes	-23.37	Macrotetrolide	441165
	831.4660	$[M+K]^+$	Yes	-24.64	Macrotetrolide	441165
Nonactin	737.4476	$[M+H]^+$	Yes	-39.31	Macrotetrolide	72519
	754.4741	$[M+NH_4]^+$	Yes	-40.68	Macrotetrolide	72519
	759.4295	$[M+Na]^+$	No	N/A	N/A	N/A
	775.4034	$[M+K]^+$	No	N/A	N/A	N/A

HR-LC-MS(MS) Spectra

Monactin spectra:

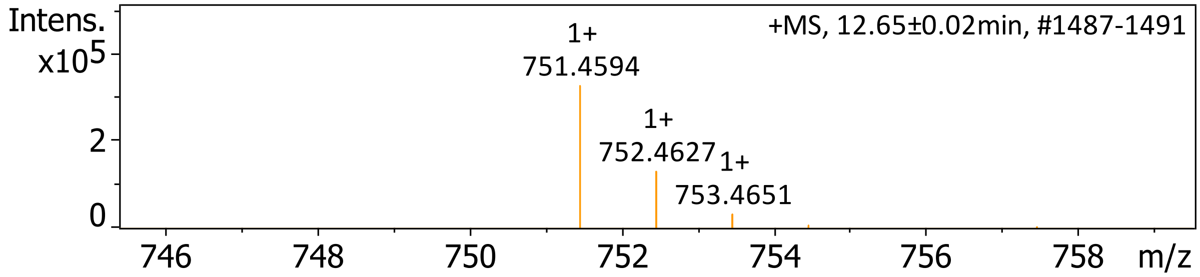

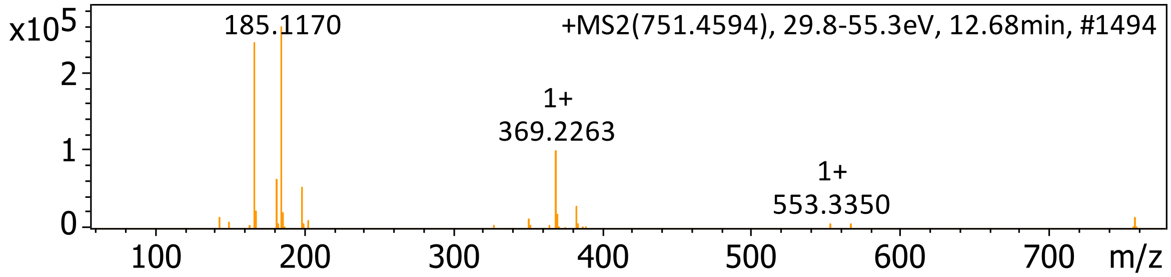

Dinactin Spectra:

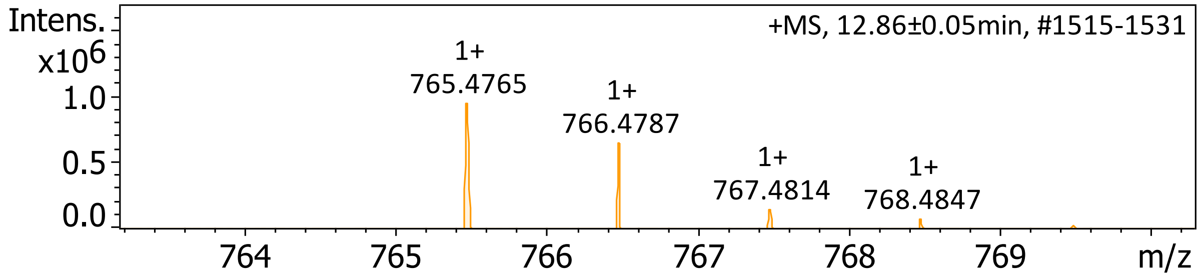

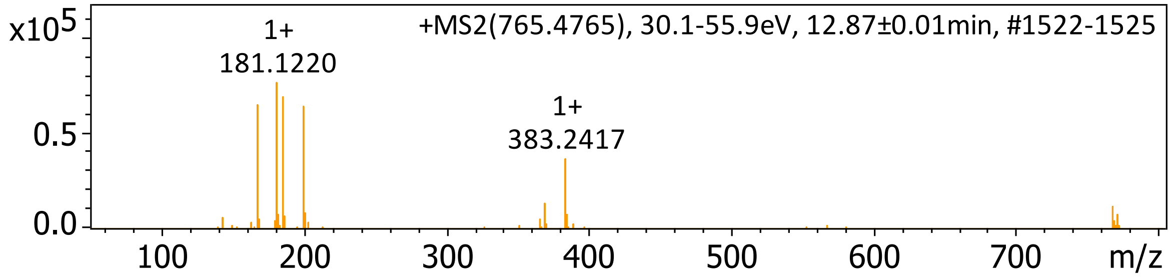

Trinactin Spectra:

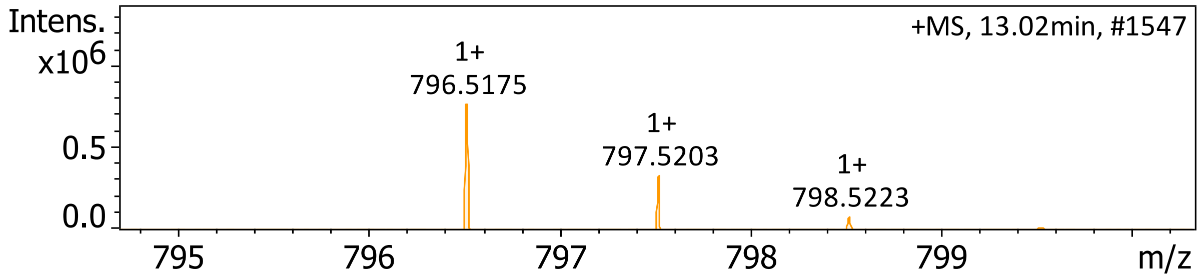

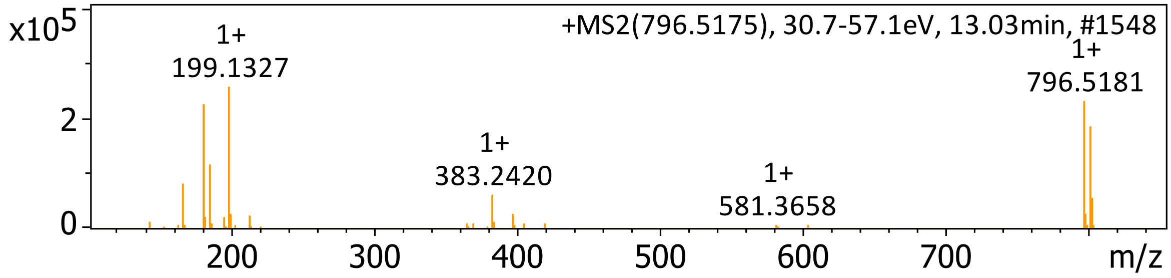

Tetranactin Spectra:

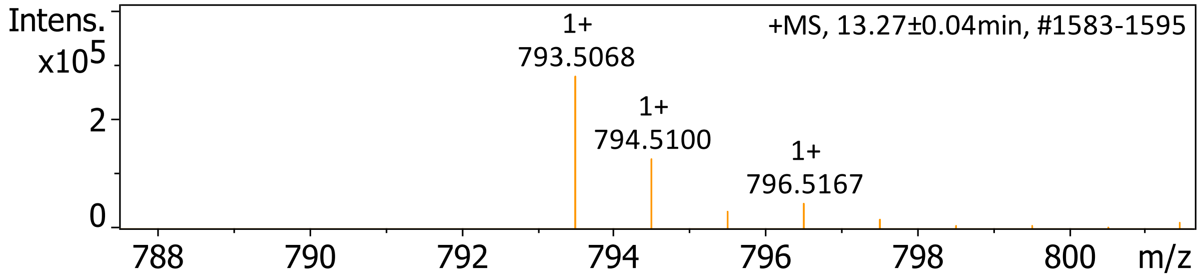

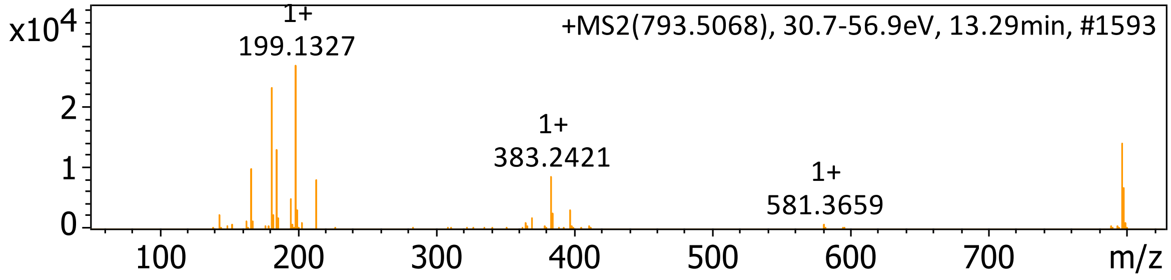

Nonactin Spectra:

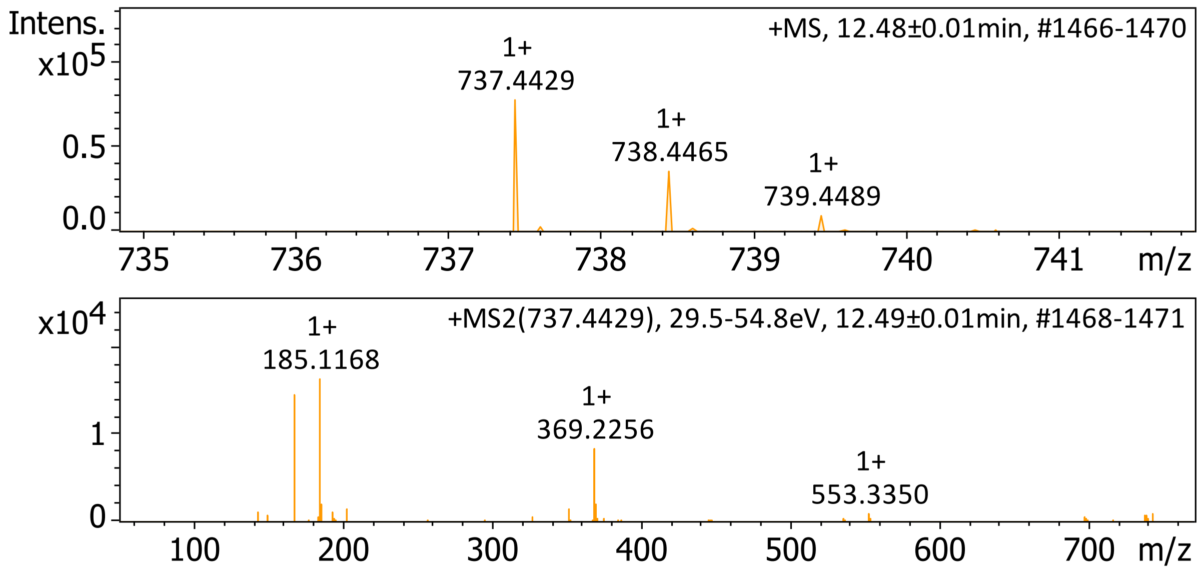

Dataset S2.4. SID9913-H6 Results: These data constitute dereplication of the bacterial extract SID9913-H6 using SIRIUS 5, GNPS, and HR-LC-MS/MS. Presented are the GNPS spectra which match macrotetrolide spectra within the MSMS data of the extract, SIRIUS 5 macrotetrolide compound identifications with their associated mass, adduct, CSI Confidence Score, classification, and PubChem ID, and the MS1 and MS2 spectra associated with each identified macrotetrolide compound.

GNPS Results

Adduct	Compound Name	MZ Error PPM	Spectrum ID	NPClassifier Class
$[M+NH_4]^+$	Monactin	3.97111	CCMSLIB00000851861	Macrotetrolides
$[M+NH_4]^+$	Monactin	0.953067	CCMSLIB00000851861	Macrotetrolides
$[M+H]^+$	Monactin	5.6043	CCMSLIB00000851859	Macrotetrolides
$[M+H]^+$	Monactin	4.14231	CCMSLIB00000851859	Macrotetrolides
$[M+H]^+$	Monactin	0.730996	CCMSLIB00000851859	Macrotetrolides
$[M+H]^+$	Nonactin	4.05551	CCMSLIB00010109074	Macrotetrolides
$[M+H]^+$	Dinactin	0.797347	CCMSLIB00000856076	Macrotetrolides
$[M+H]^+$	Monactin	5.68552	CCMSLIB00000851859	Macrotetrolides
$[M+H]^+$	Dinactin	4.46514	CCMSLIB00000856076	Macrotetrolides
$[M+H]^+$	Trinactin	3.28864	CCMSLIB00000854224	Macrotetrolides
$[M+H]^+$	Trinactin	1.80092	CCMSLIB00000854224	Macrotetrolides
$[M+H]^+$	Nonactin	5.62805	CCMSLIB00010109074	Macrotetrolides

SIRIUS Results

Macrotetrolide Masses			Detected by SIRIUS	CSI Score	CANOPUS Class	PubChem ID Match
Monactin	751.4632	$[M+H]^+$	Yes	-7.90	Macrotetrolide	169015
	768.489	$[M+NH_4]^+$	Yes	-12.39	Macrotetrolide	169015
	773.4467	$[M +Na]^+$	No	N/A	N/A	N/A
	789.419	$[M+K]^+$	Yes	-11.49	Macrotetrolide	169015
Dinactin	765.4789	$[M+H]^+$	Yes	-6.25	Macrotetrolide	6916048
	782.5054	$[M+NH_4]^+$	Yes	-5.34	Macrotetrolide	6916048
	787.460	$[M+Na]^+$	No	N/A	N/A	N/A
	803.434	$[M+K]^+$	No	N/A	N/A	N/A
Trinactin	779.4945	$[M+H]^+$	Yes	-4.26	Macrotetrolide	169021
	796.5211	$[M+NH_4]^+$	Yes	-4.82	Macrotetrolide	169021
	801.4765	$[M+Na]^+$	No	N/A	N/A	N/A
	817.4504	$[M+K]^+$	Yes	-8.16	Macrotetrolide	169021
Tetranactin	793.5102	$[M+H]^+$	No	N/A	N/A	N/A
	810.5367	$[M+NH_4]^+$	Yes	-47.19	Macrotetrolide	441165
	815.4921	$[M+Na]^+$	No	N/A	N/A	N/A
	831.4660	$[M+K]^+$	No	N/A	N/A	N/A
Nonactin	737.4476	$[M+H]^+$	No	N/A	N/A	N/A
	754.4741	$[M+NH_4]^+$	No	N/A	N/A	N/A
	759.4295	$[M+Na]^+$	No	N/A	N/A	N/A
	775.4034	$[M+K]^+$	No	N/A	N/A	N/A

HR-LC-MS(MS) Spectra:

Monactin Spectrum:

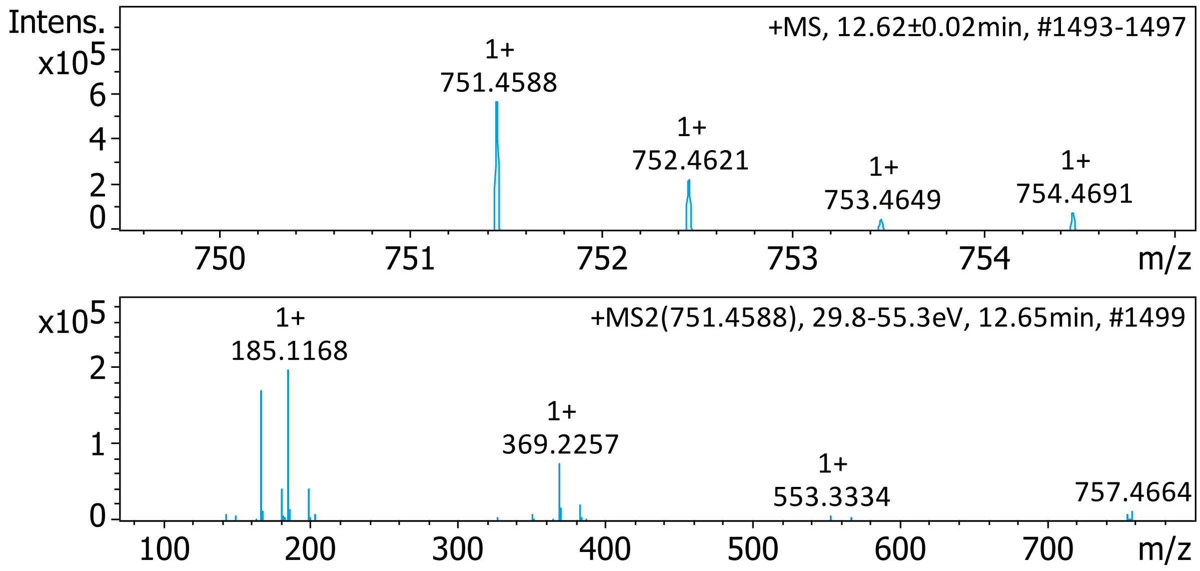

Dinactin Spectrum:

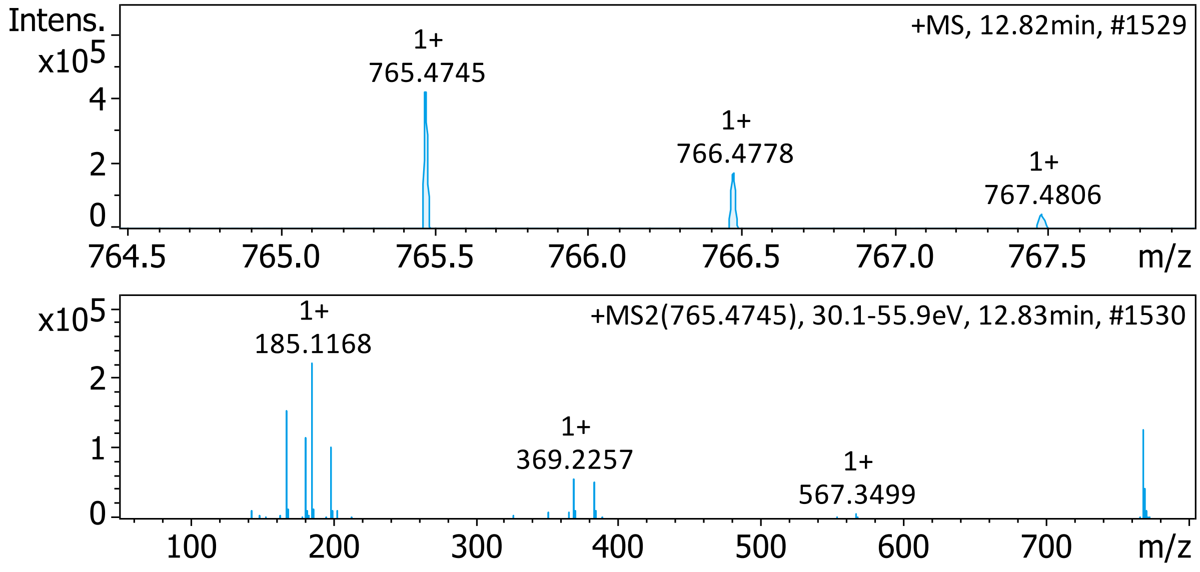

Trinactin Spectrum:

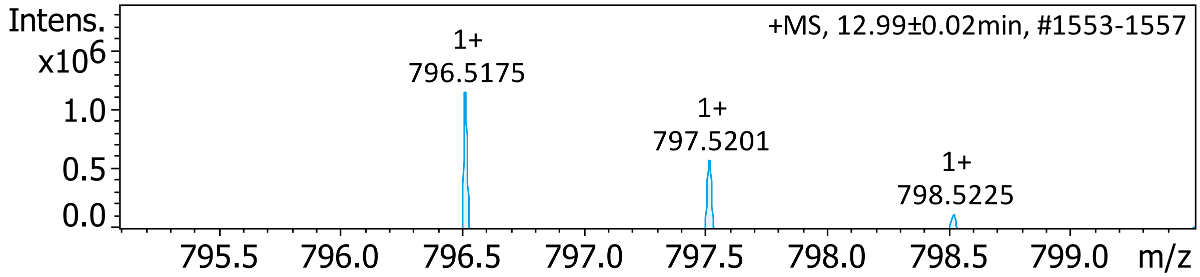

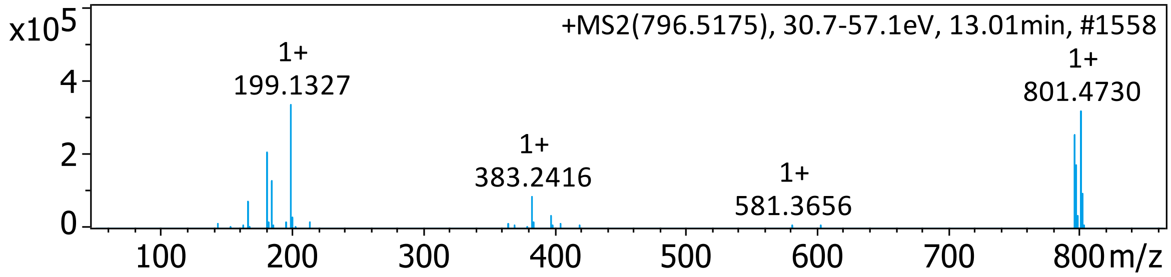

Tetranactin Spectrum:

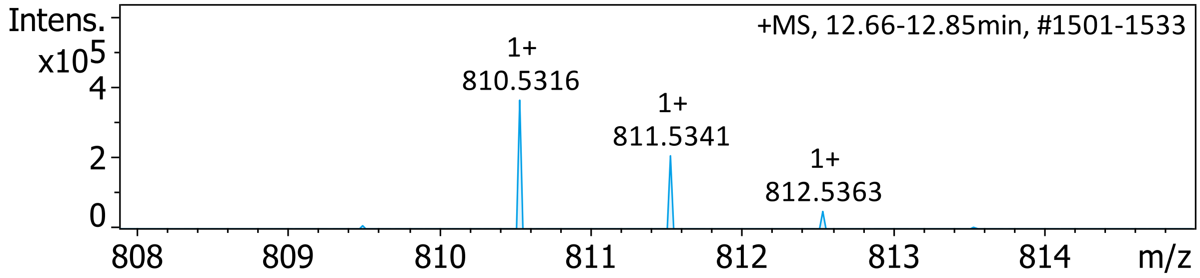

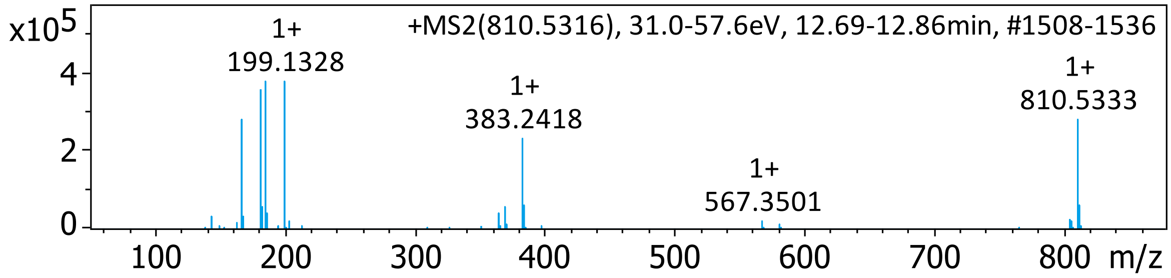

Dataset S2.5. SID8465-H6 Results: These data constitute dereplication of the bacterial extract SID8465-H6 using SIRIUS 5, GNPS, and HR-LC-MS/MS. Presented are the GNPS spectra which match macrotetrolide spectra within the MSMS data of the extract, SIRIUS 5 macrotetrolide compound identifications with their associated mass, adduct, CSI Confidence Score, classification, and PubChem ID, and the MS1 and MS2 spectra associated with each identified macrotetrolide compound.

GNPS Match Results:

Adduct	Compound Name	MZ Error PPM	Spectrum ID	NPClassifier Class
$[M+H]^+$	Monactin	2.76154	CCMSLIB00000851859	Macrotetrolides
$[M+NH_4]^+$	Monactin	1.82671	CCMSLIB00000851861	Macrotetrolides
$[M+H]^+$	Monactin	3.24887	CCMSLIB00000851859	Macrotetrolides
$[M+NH_4]^+$	Monactin	0.397111	CCMSLIB00000851861	Macrotetrolides
$[M+H]^+$	Nonactin	0.579358	CCMSLIB00010109074	Macrotetrolides
$[M+H]^+$	Dinactin	1.8339	CCMSLIB00000856076	Macrotetrolides
$[M+H]^+$	Nonactin	2.56573	CCMSLIB00010109074	Macrotetrolides
$[M+H]^+$	Dinactin	0	CCMSLIB00000856076	Macrotetrolides
$[M+H]^+$	Trinactin	1.64432	CCMSLIB00000854224	Macrotetrolides
$[M+H]^+$	Trinactin	1.09621	CCMSLIB00000854224	Macrotetrolides
$[M+H]^+$	Nonactin	2.89679	CCMSLIB00010109074	Macrotetrolides
$[M+H]^+$	Trinactin	2.58393	CCMSLIB00000854224	Macrotetrolides
$[M+H]^+$	Dinactin	3.26912	CCMSLIB00000856076	Macrotetrolides

SIRIUS Results:

Macrotetrolide Masses			Detected by SIRIUS	CSI Score	CANOPUS Class	PubChem ID Match
Monactin	751.4632	$[M+H]^+$	Yes	-12.77	Macrotetrolide	169015
	768.489	$[M+NH_4]^+$	Yes	-11.78	Macrotetrolide	169015
	773.4467	$[M +Na]^+$	No	N/A	N/A	N/A
	789.419	$[M+K]^+$	Yes	-9.98	Macrotetrolide	169015
Dinactin	765.4789	$[M+H]^+$	Yes	-5.35	Macrotetrolide	6916048
	782.5054	$[M+NH_4]^+$	Yes	-4.64	Macrotetrolide	6916048
	787.460	$[M+Na]^+$	No	N/A	N/A	N/A
	803.434	$[M+K]^+$	Yes	-4.94	Macrotetrolide	6916048
Trinactin	779.4945	$[M+H]^+$	Yes	-4.38	Macrotetrolide	169021
	796.5211	$[M+NH_4]^+$	Yes	-5.38	Macrotetrolide	169021
	801.4765	$[M+Na]^+$	No	N/A	N/A	N/A
	817.4504	$[M+K]^+$	Yes	-9.87	Macrotetrolide	169021
Tetranactin	793.5102	$[M+H]^+$	No	N/A	N/A	N/A

	810.5367	$[M+NH_4]^+$	Yes	-44.65	Macrotetrolide	
	815.4921	$[M+Na]^+$	No	N/A	N/A	N/A
	831.4660	$[M+K]^+$	No	N/A	N/A	N/A
Nonactin	737.4476	$[M+H]^+$	No	N/A	N/A	N/A
	754.4741	$[M+NH_4]^+$	No	N/A	N/A	N/A
	759.4295	$[M+Na]^+$	No	N/A	N/A	N/A
	775.4034	$[M+K]^+$	No	N/A	N/A	N/A

HR-LC-MS(MS) Spectra:

Monactin Spectrum:

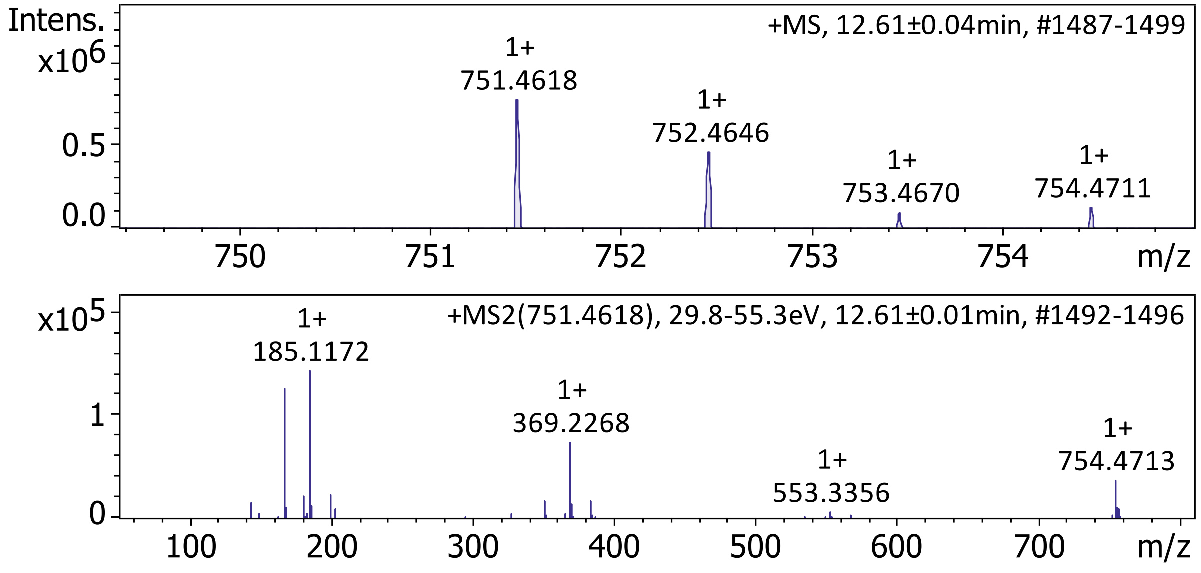

Dinactin Spectrum:

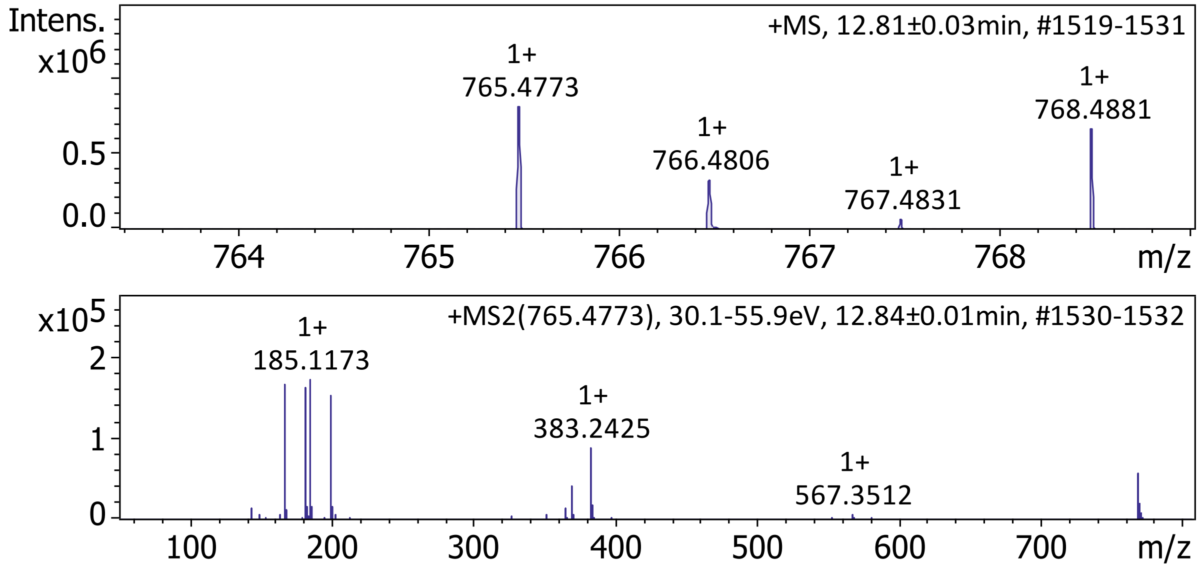

Trinactin Spectrum:

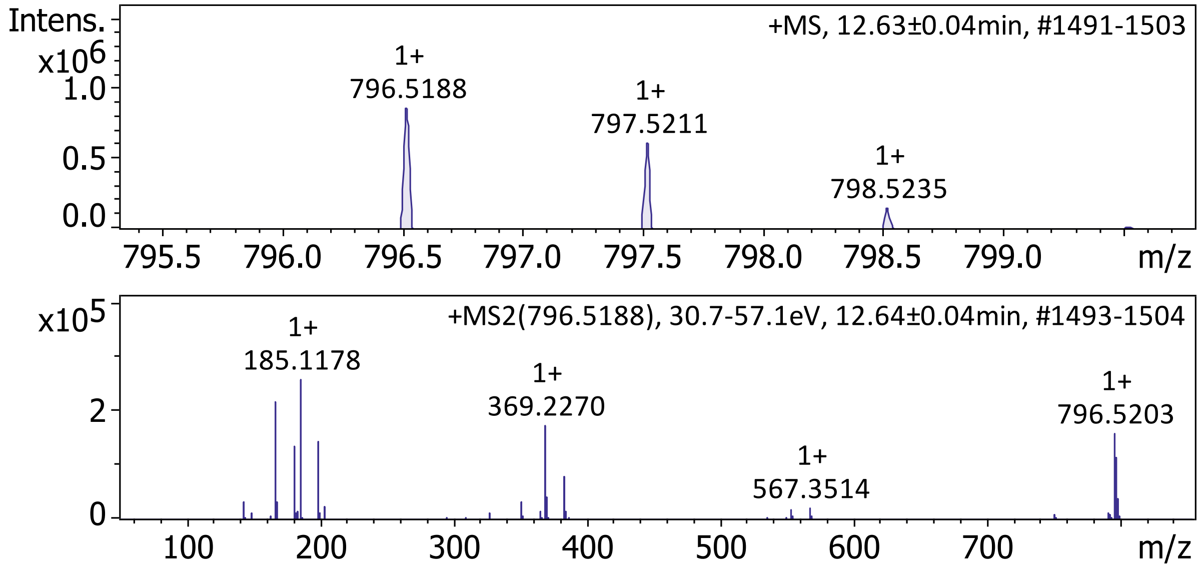

Tetranactin Spectrum:

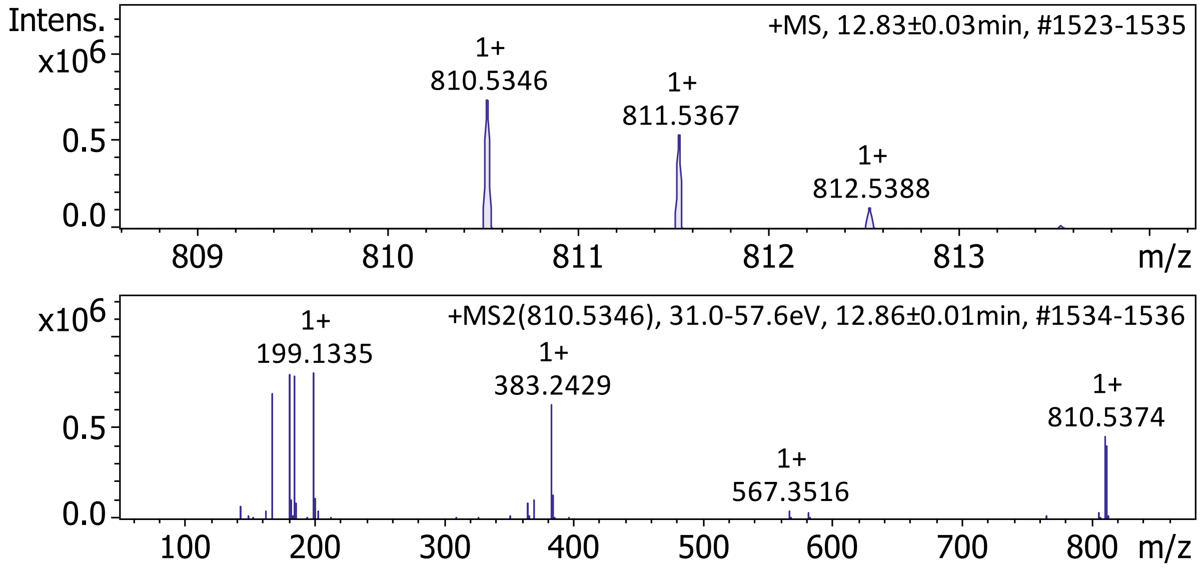

Dataset S3. Polyene identification data. LC-UV-MSMS spectra collected for fractions SID4915 – E4, SID4921 – E6, SID4921 – E7, SID4921 – G7, SID8366 – E9, SID8366 – G6, SID8366 – G7, SID11285 – E8, and SID11285 – G7 as listed below. Depictions of UV and MS1 spectra for each extract are stacked [sometimes at multiple retention times (*Rt*)] and UV signatures often resemble those of polyene antifungal signatures. See **Figure S5** for standard polyene signature spectra.

SID4915 – E4 LC-UV-MS (*Rt* = 6.78 – 6.81 min)
SID4921 – E6 LC-UV-MS (*Rt* = 6.52 – 6.55 min)
SID4921 – E6 LC-UV-MS (*Rt* = 7.23 – 7.26 min)
SID4921 – E6 LC-UV-MS (*Rt* = 7.92 – 7.95 min)
SID4921 – E7 LC-UV-MS (*Rt* = 7.922 – 7.932 min)
SID4921 – E7 LC-UV-MS (*Rt* = 8.32 – 8.38 min)
SID4921 – E7 LC-UV-MS (*Rt* = 8.52 – 8.56 min)
SID4921 – G7 LC-UV-MS (*Rt* = 8.19 – 8.21 min)
SID4921 – G7 LC-UV-MS (*Rt* = 8.53 – 8.58 min)
SID4921 – G7 LC-UV-MS (*Rt* = 8.91 – 8.94 min)
SID4921 – G7 LC-UV-MS (*Rt* = 9.14 – 9.18 min)
SID7982 – G7 LC-UV-MS/MS (*Rt* = 9.65 – 9.68 min)
SID8366 – E9 LC-UV-MS/MS (*Rt* = 9.13 – 9.17 min)
SID8366 – E9 LC-UV-MS/MS (*Rt* = 10.07 – 10.10 min)
SID8366 – G6 LC-UV-MS (*Rt* = 8.18 – 8.22 min)
SID8366 – G6 LC-UV-MS (*Rt* = 8.87 – 8.94 min)
SID8366 – G7 LC-UV-MS/MS (*Rt* = 8.16 – 8.23 min)
SID8366 – G7 LC-UV-MS/MS (*Rt* = 8.53 – 8.56 min)
SID8366 – G7 LC-UV-MS/MS (*Rt* = 8.89 – 8.95 min)
SID8366 – G7 LC-UV-MS/MS (*Rt* = 9.15 – 9.20 min)
SID11285 – E8 LC-UV-MS/MS (*Rt* = 9.34 – 9.39 min)
SID11285 – G7 LC-UV-MS/MS (*Rt* = 9.46 – 9.52 min)

SID4915 – E4 LC-UV-MS (*Rt* = 6.78 – 6.81 min):

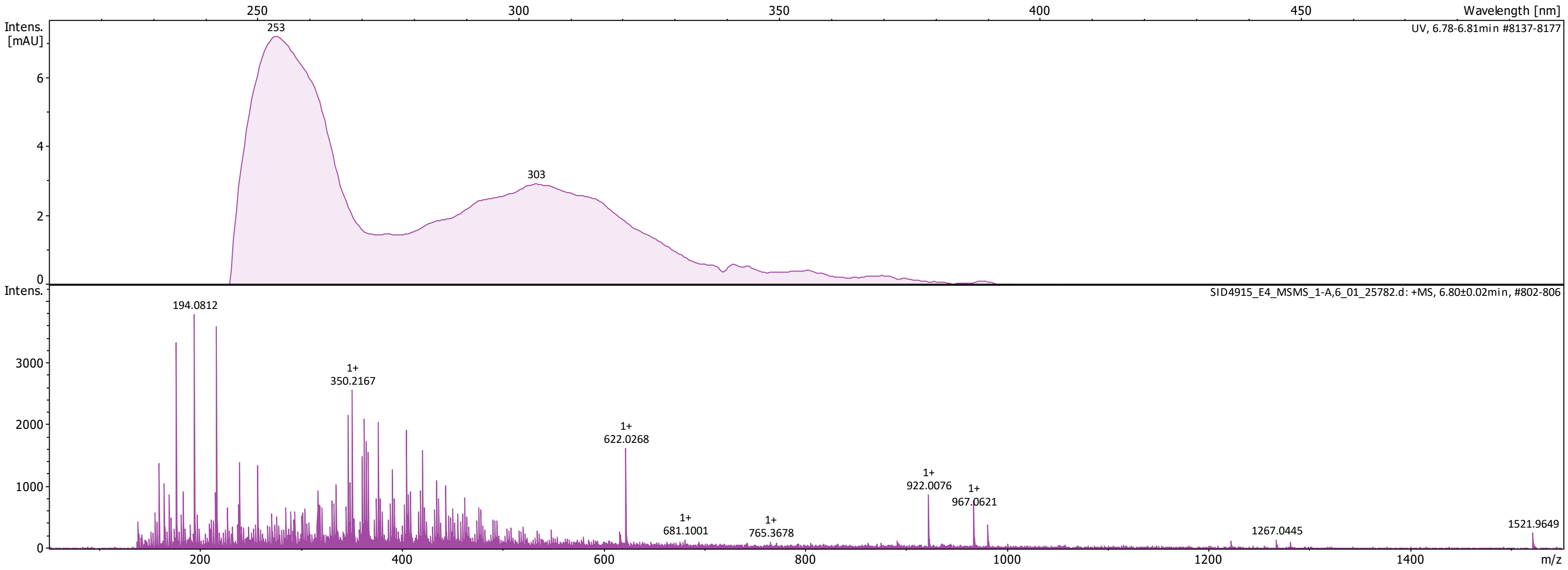

SID4921 – E6 LC-UV-MS (*Rt* = 6.52 – 6.55 min):

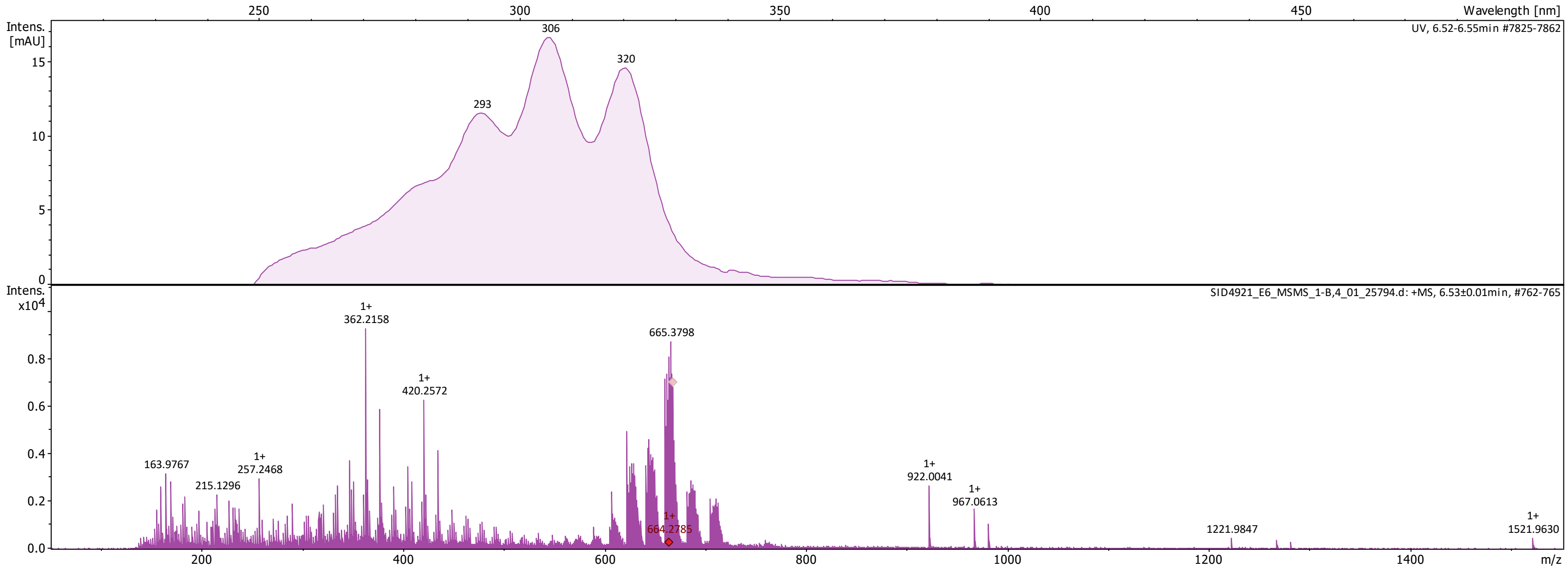

SID4921 – E6 LC-UV-MS (*Rt* = 7.23 – 7.26 min):

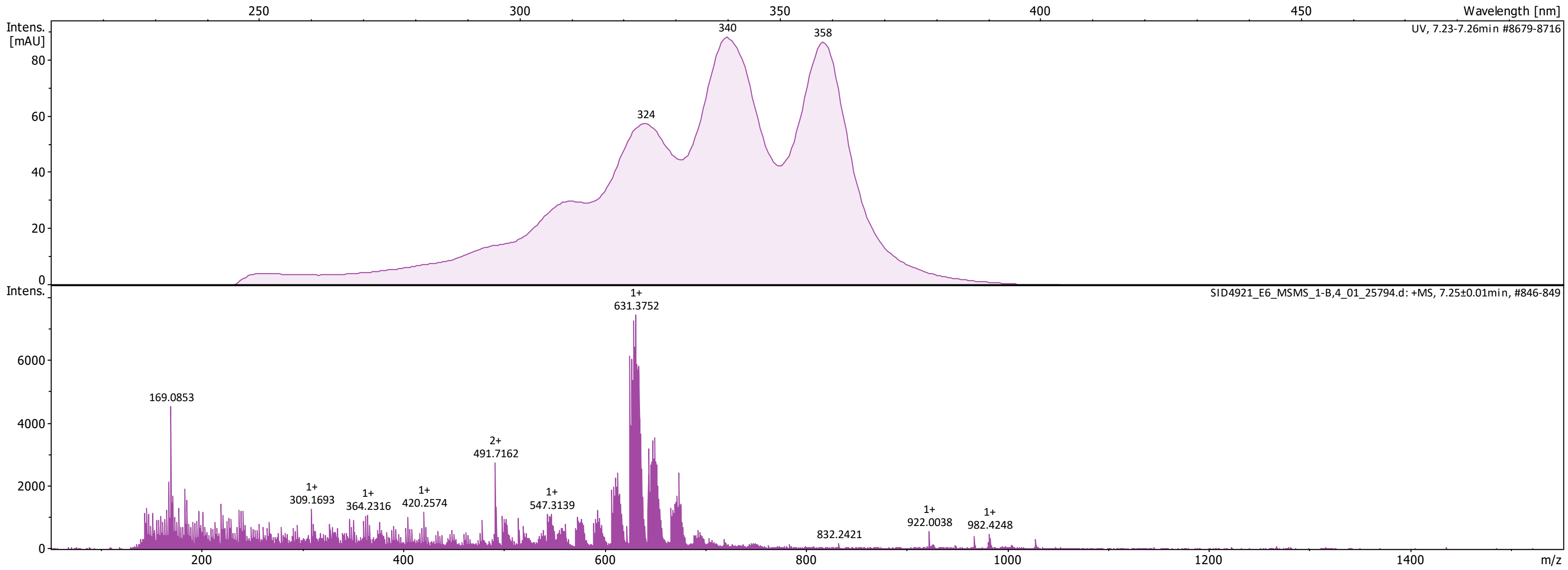

SID4921 – E6 LC-UV-MS (*Rt* = 7.92 – 7.95 min):

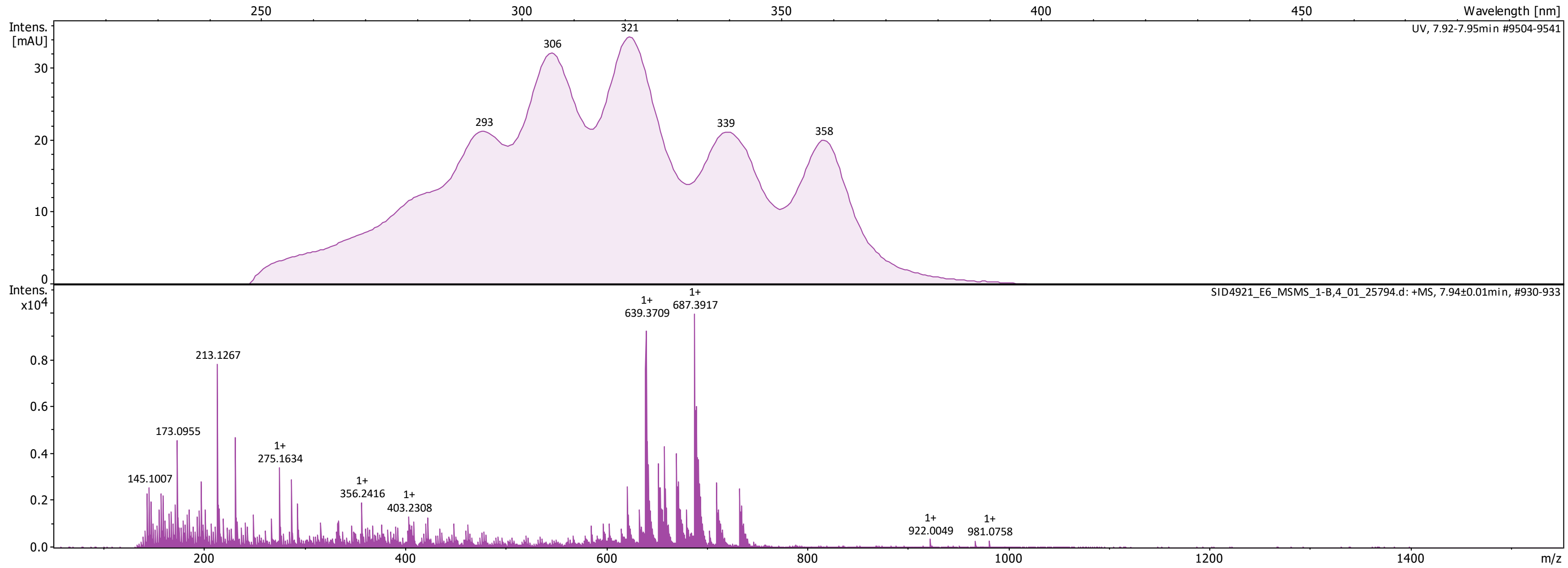

SID4921 – E7 LC-UV-MS (*Rt* = 7.922 – 7.932 min):

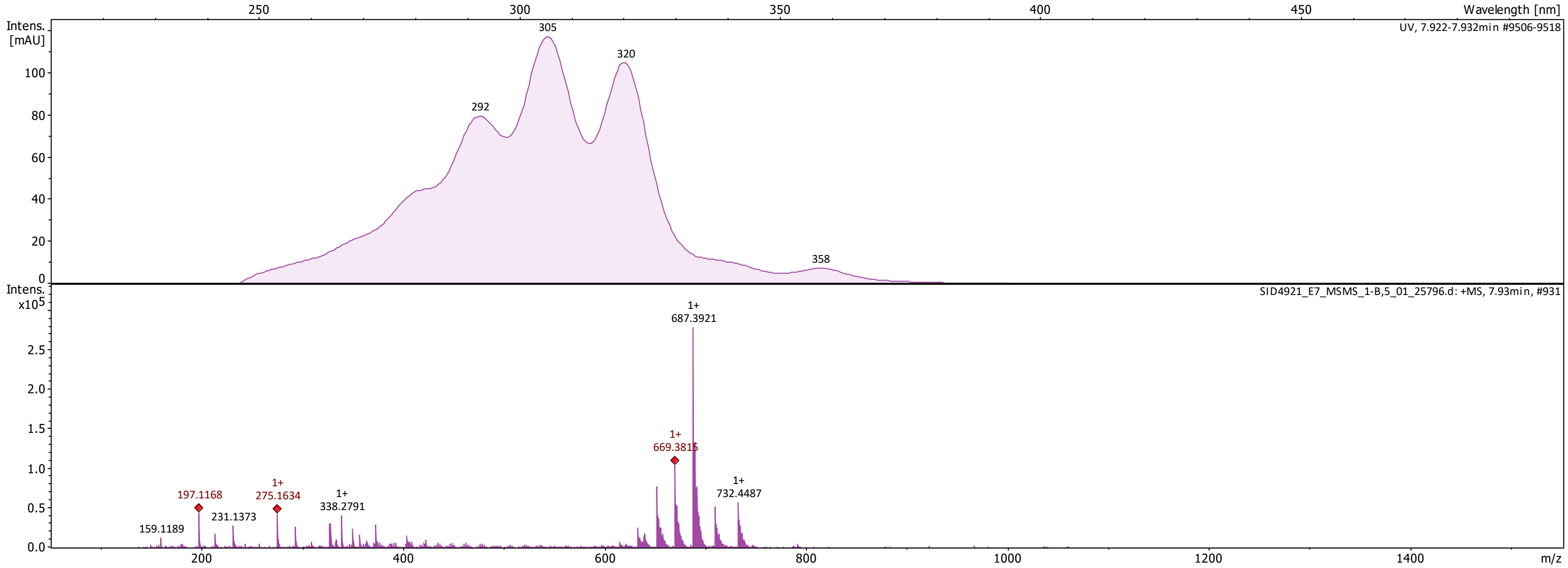

SID4921 – E7 LC-UV-MS (*Rt* = 8.32 – 8.38 min):

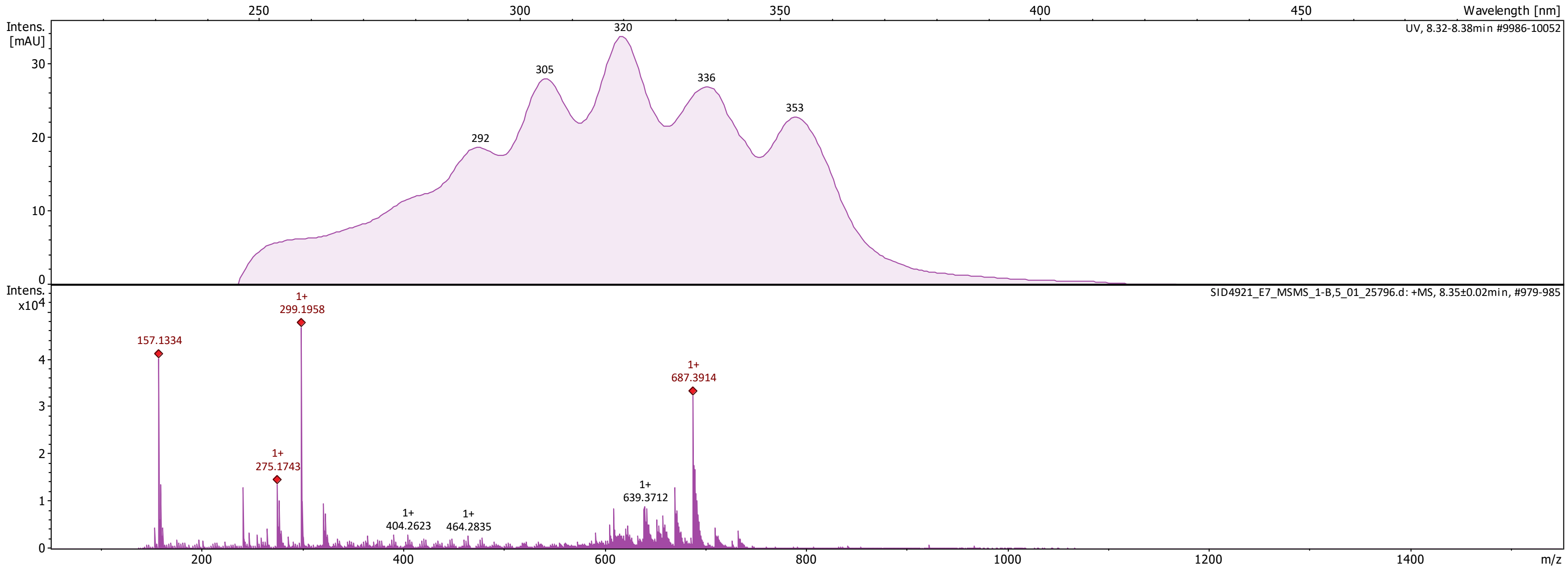

SID4921 – E7 LC-UV-MS (*Rt* = 8.52 – 8.56 min):

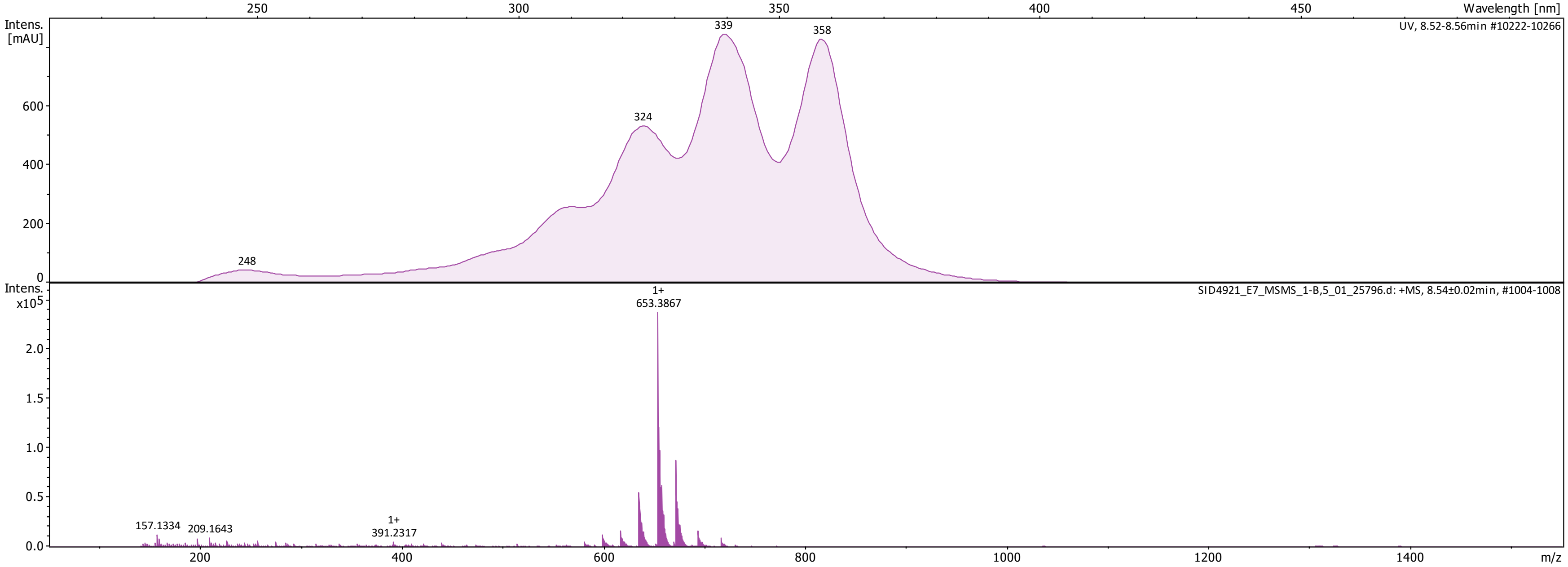

SID4921 – G7 LC-UV-MS (*Rt* = 8.19 – 8.21 min):

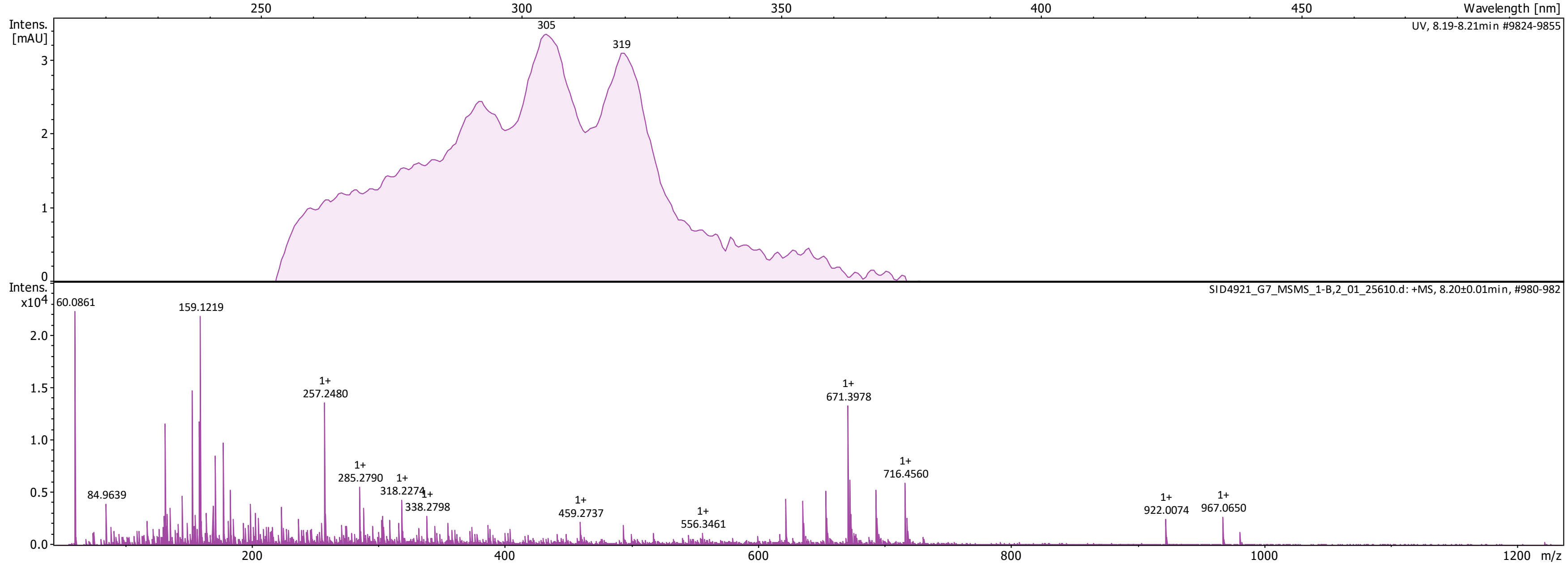

SID4921 – G7 LC-UV-MS (*Rt* = 8.53 – 8.58 min):

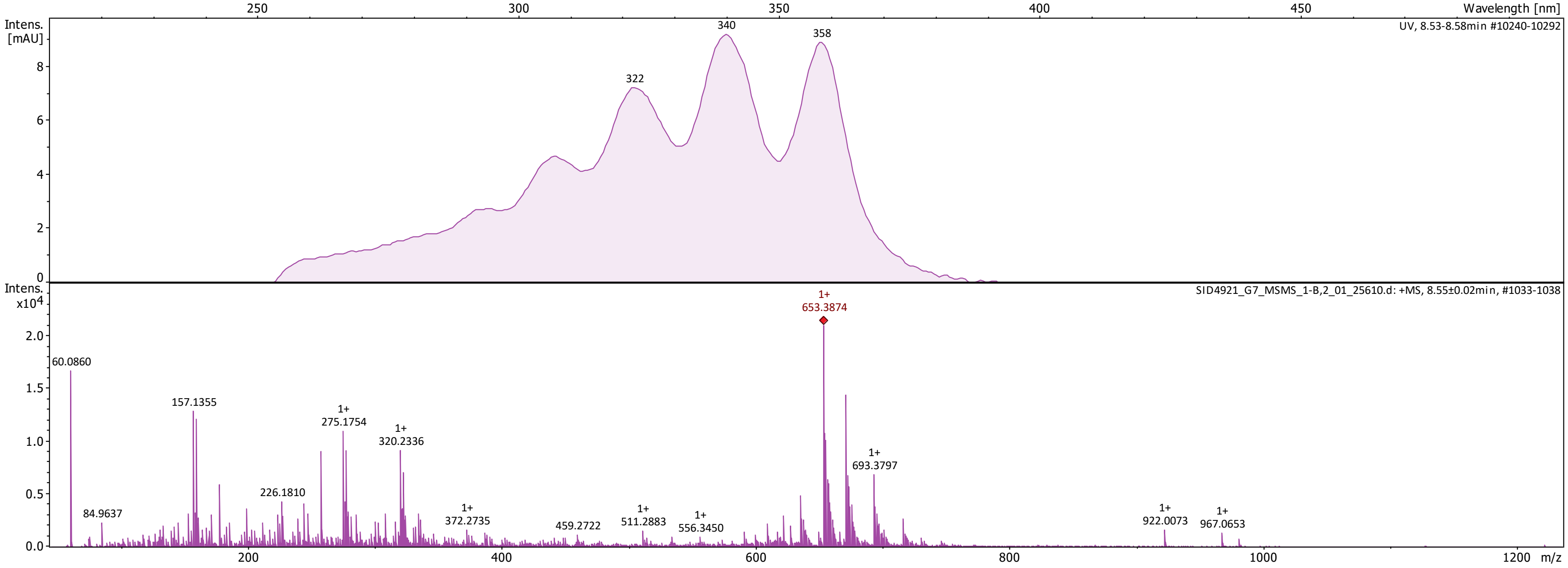

SID4921 – G7 LC-UV-MS (*Rt* = 8.91 – 8.94 min):

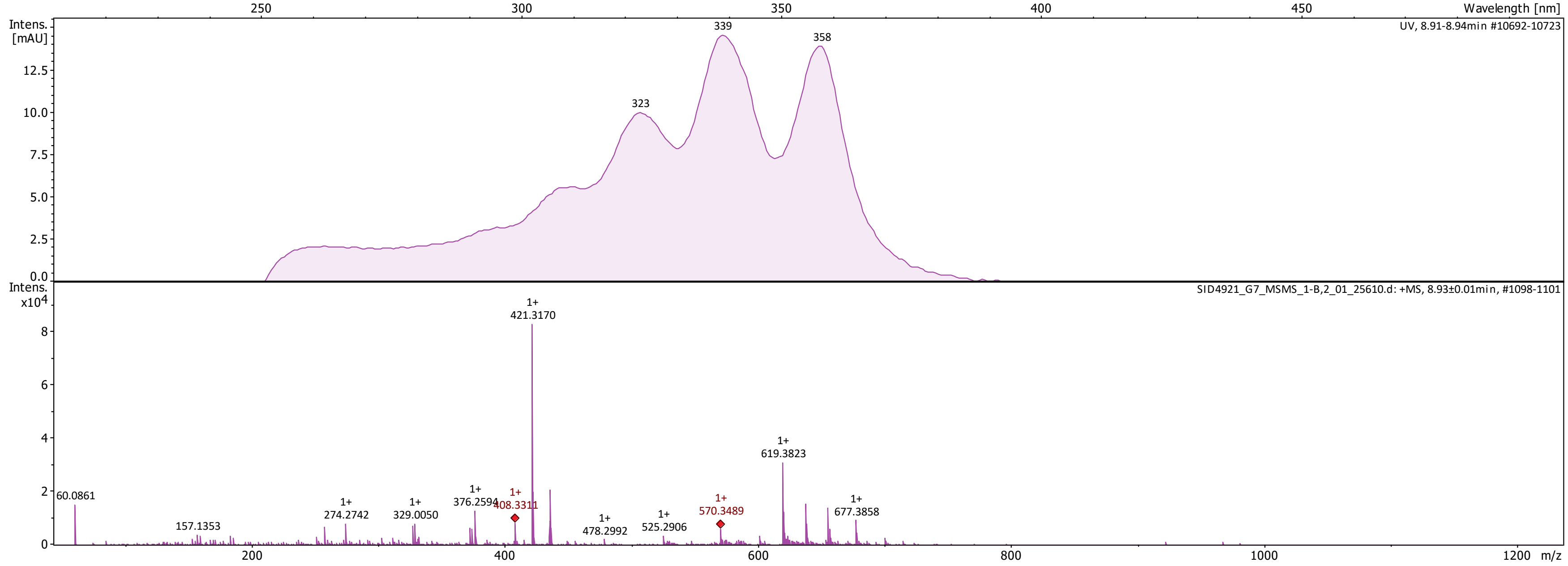

SID4921 – G7 LC-UV-MS (*Rt* = 9.14 – 9.18 min):

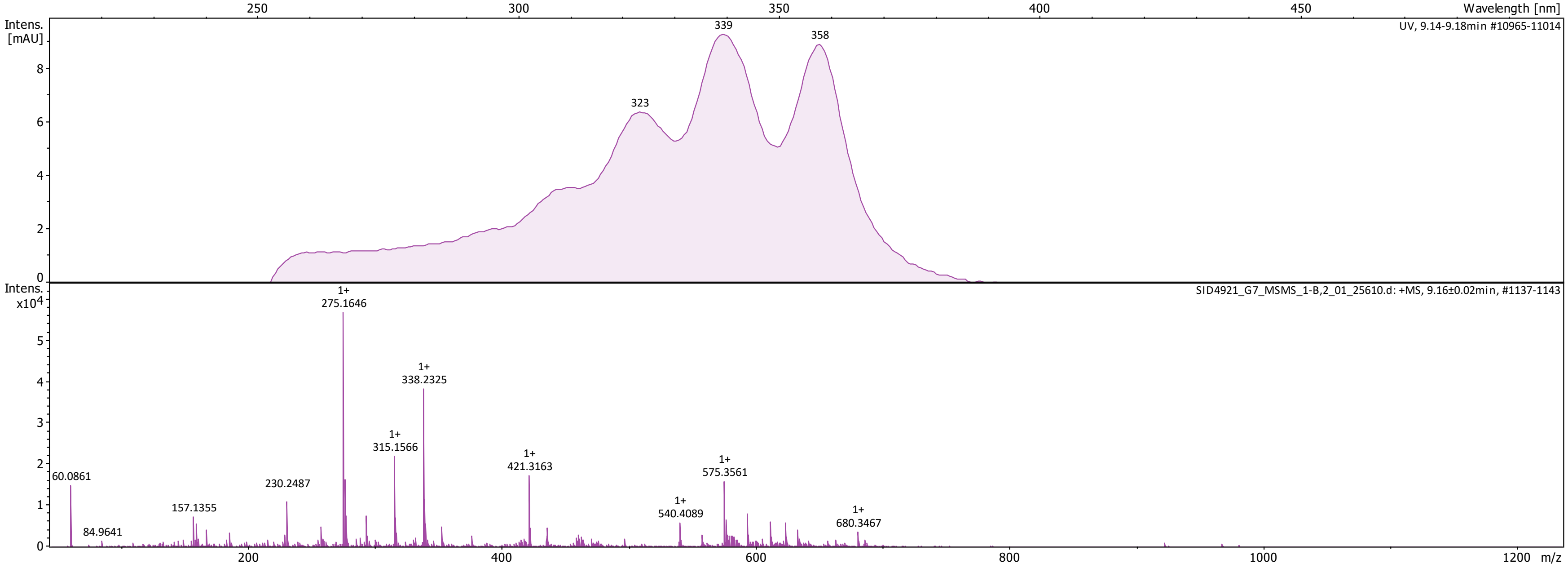

SID7982 – G7 LC-UV-MS/MS (*Rt* = 9.65 – 9.68 min):

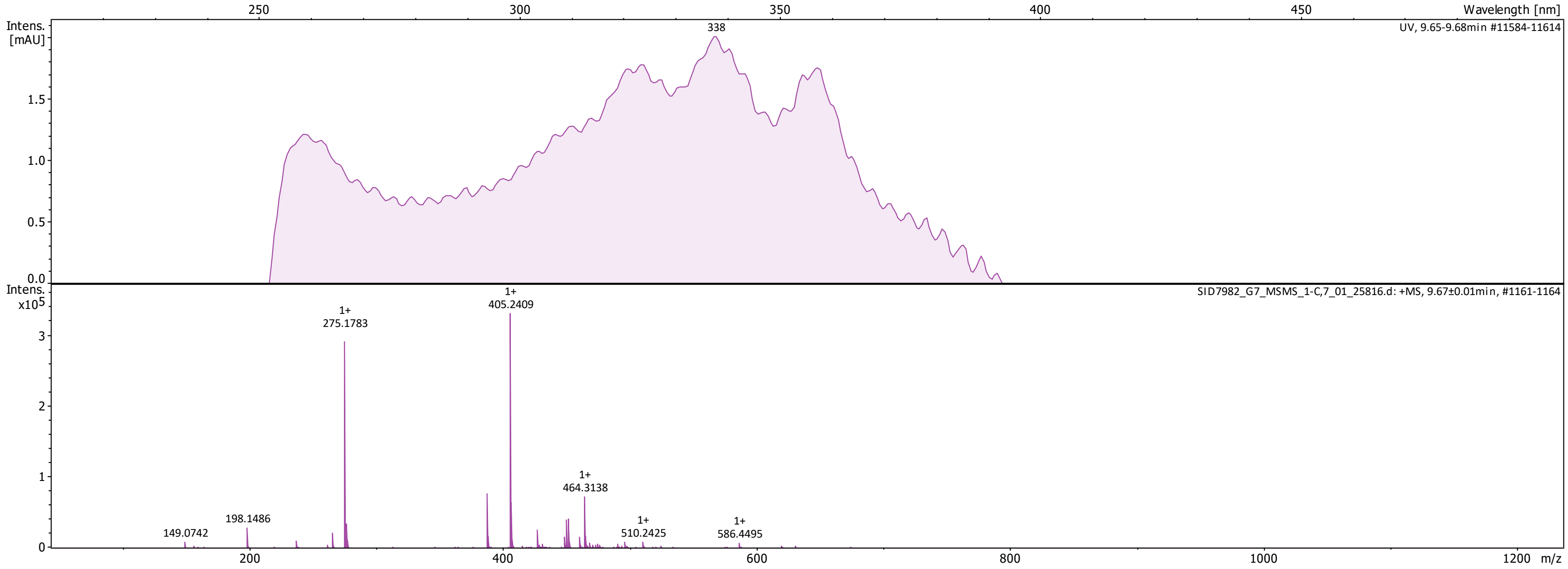

SID8366 – E9 LC-UV-MS/MS (*Rt* = 9.13 – 9.17 min):

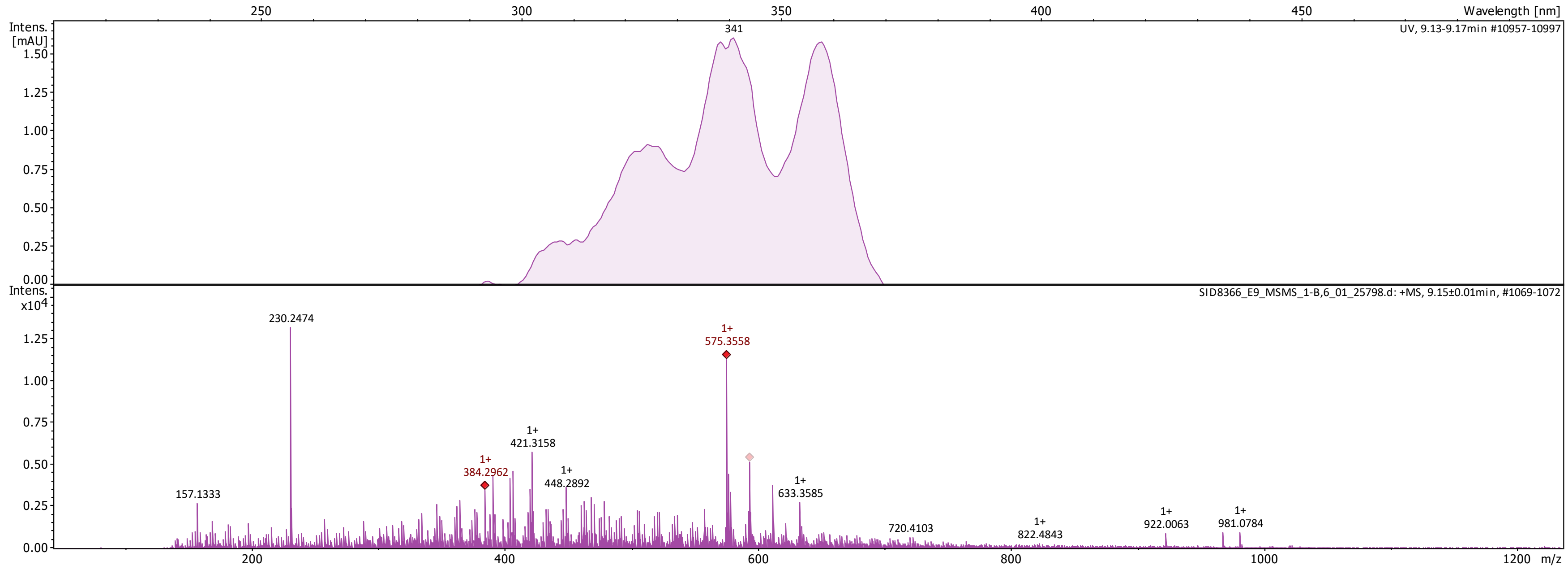

SID8366 – E9 LC-UV-MS/MS (*Rt* = 10.07 – 10.10 min):

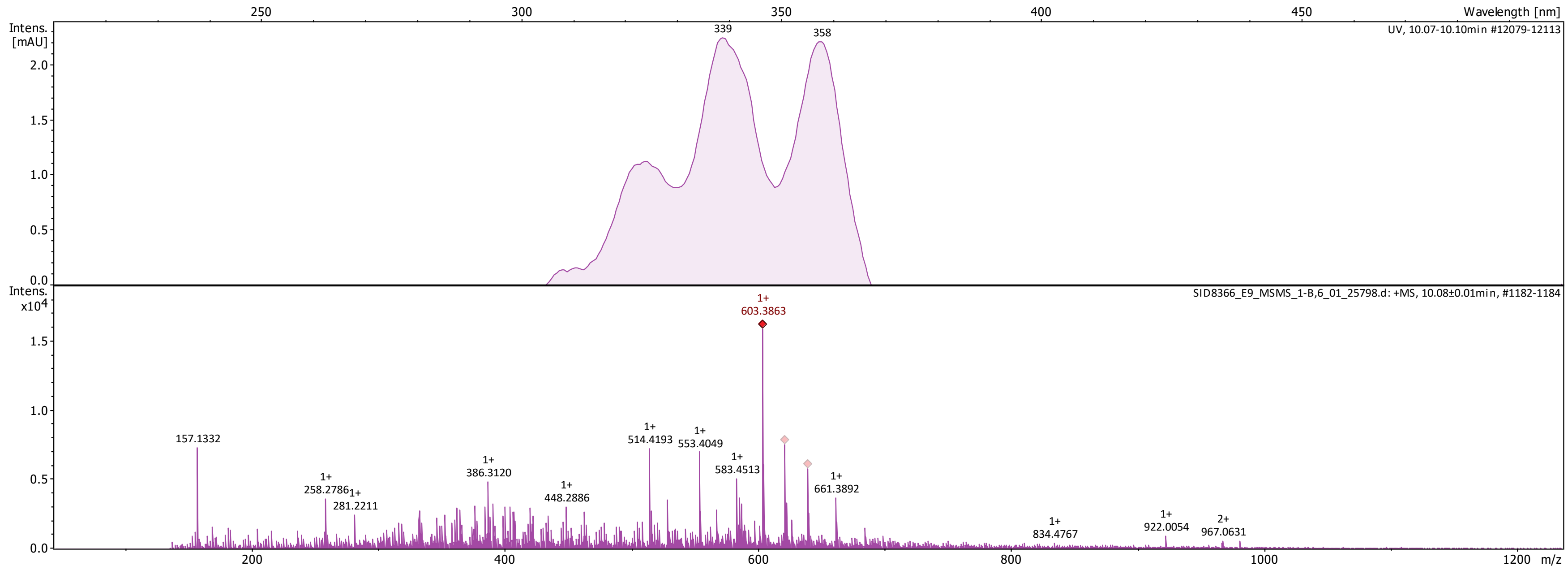

SID8366 – G6 LC-UV-MS (*Rt* = 8.18 – 8.22 min):

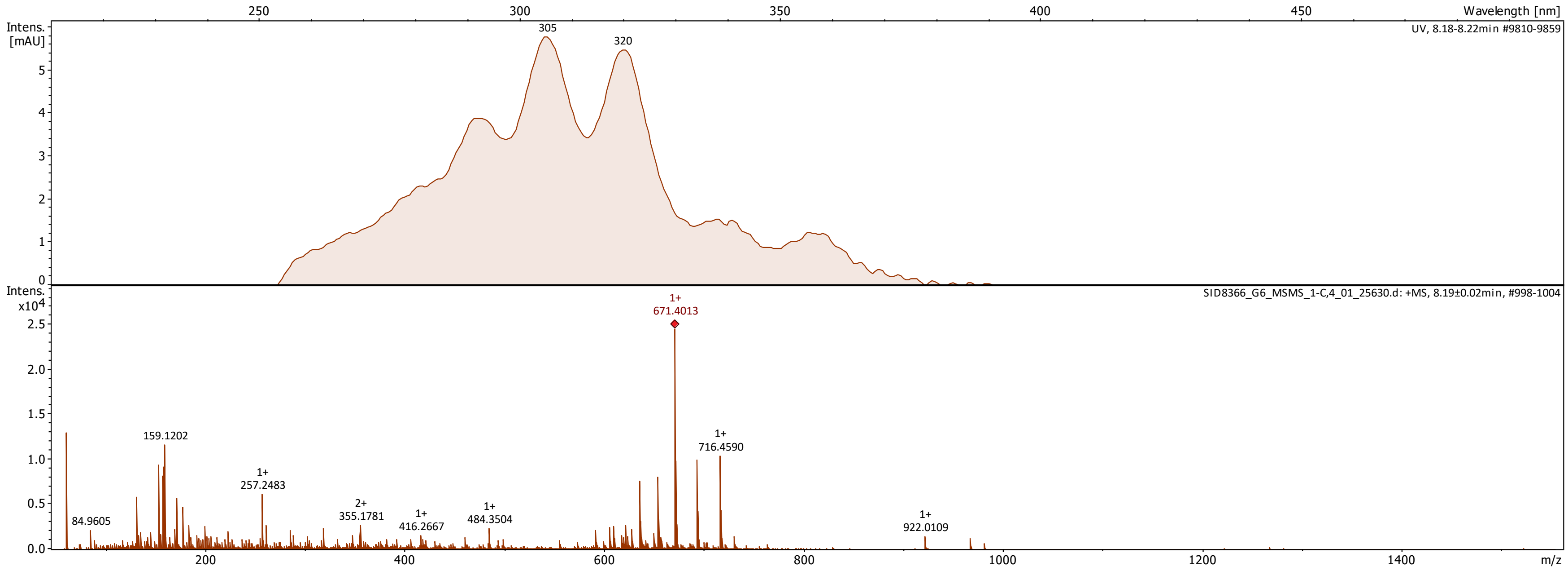

SID8366 – G6 LC-UV-MS (*Rt* = 8.87 – 8.94 min):

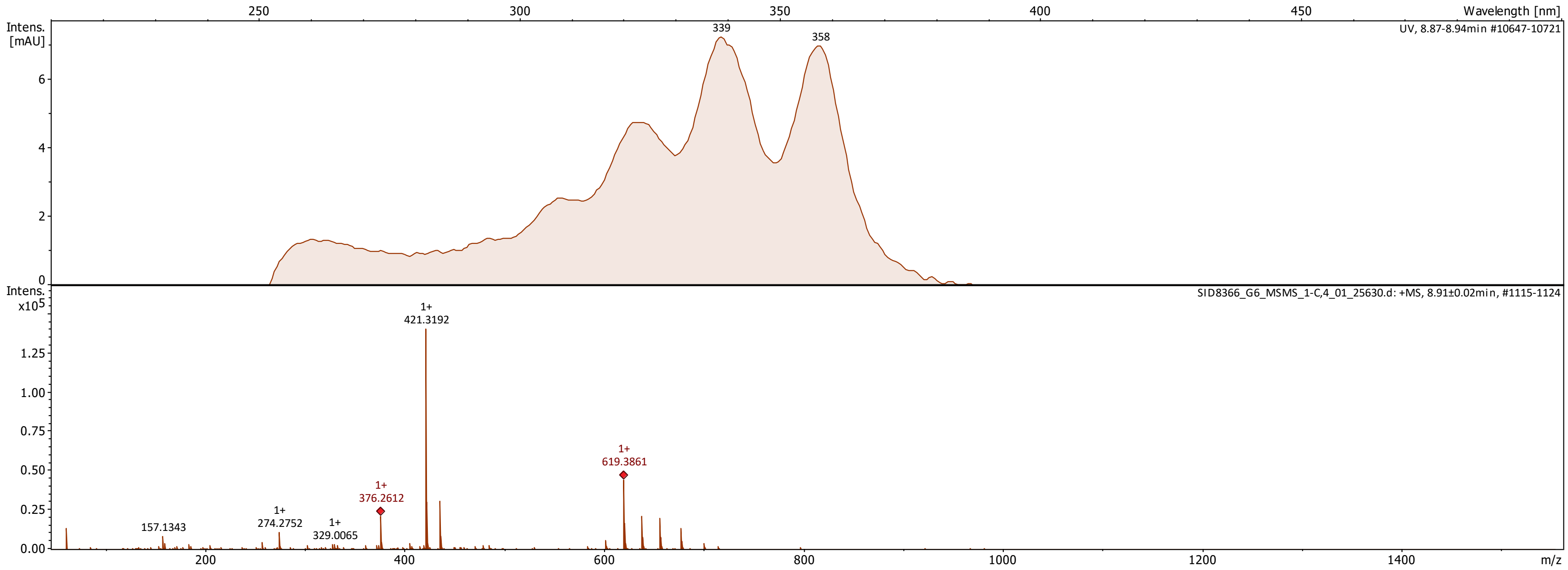

SID8366 – G7 LC-UV-MS/MS (*Rt* = 8.16 – 8.23 min):

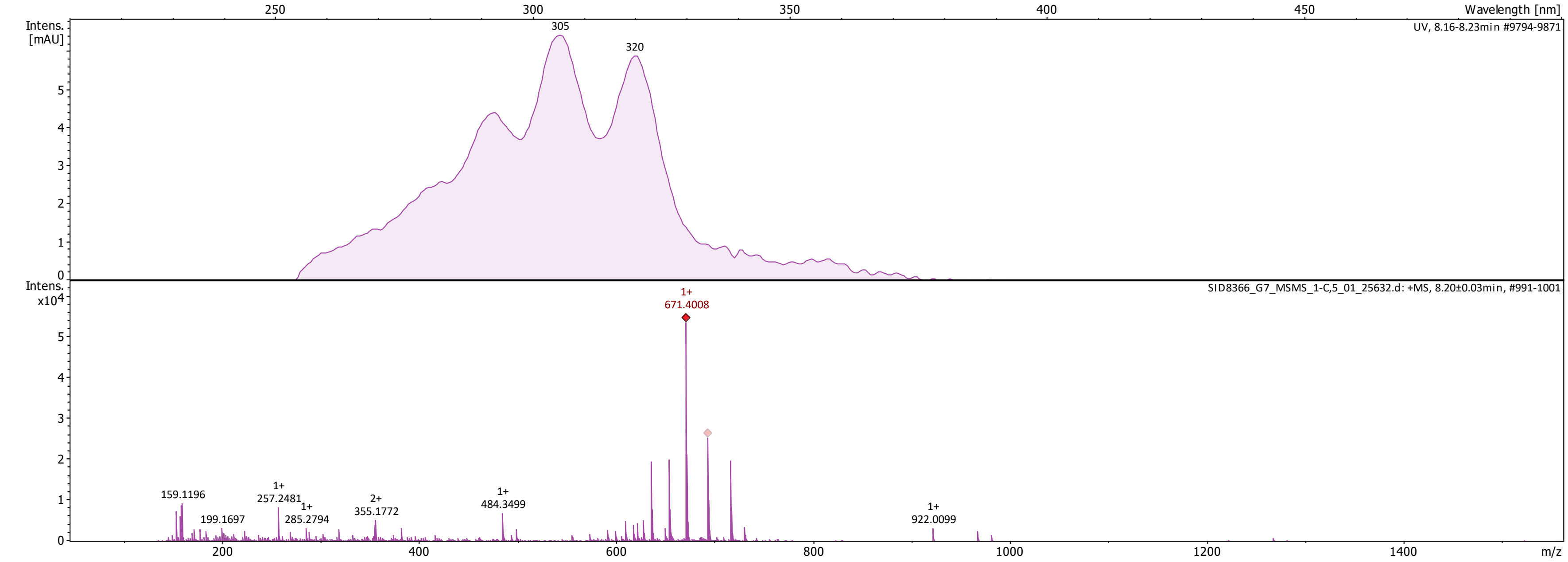

SID8366 – G7 LC-UV-MS/MS (*Rt* = 8.53 – 8.56 min):

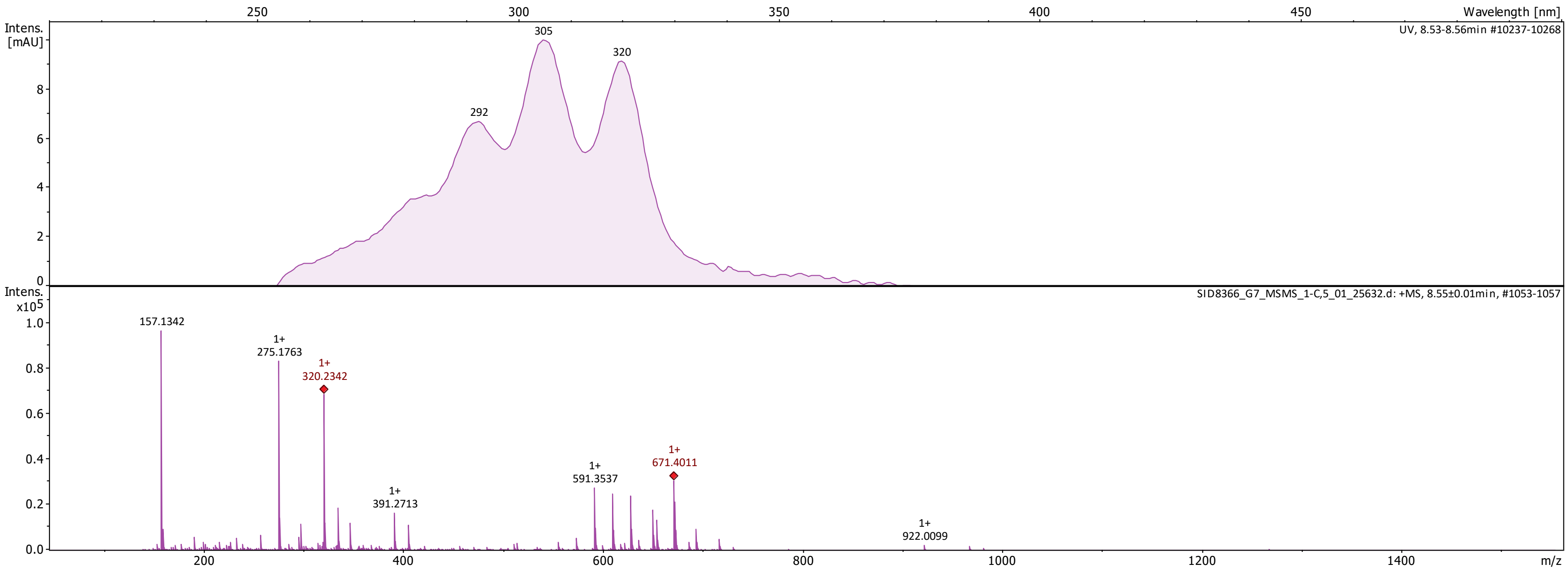

SID8366 – G7 LC-UV-MS/MS (*Rt* = 8.89 – 8.95 min):

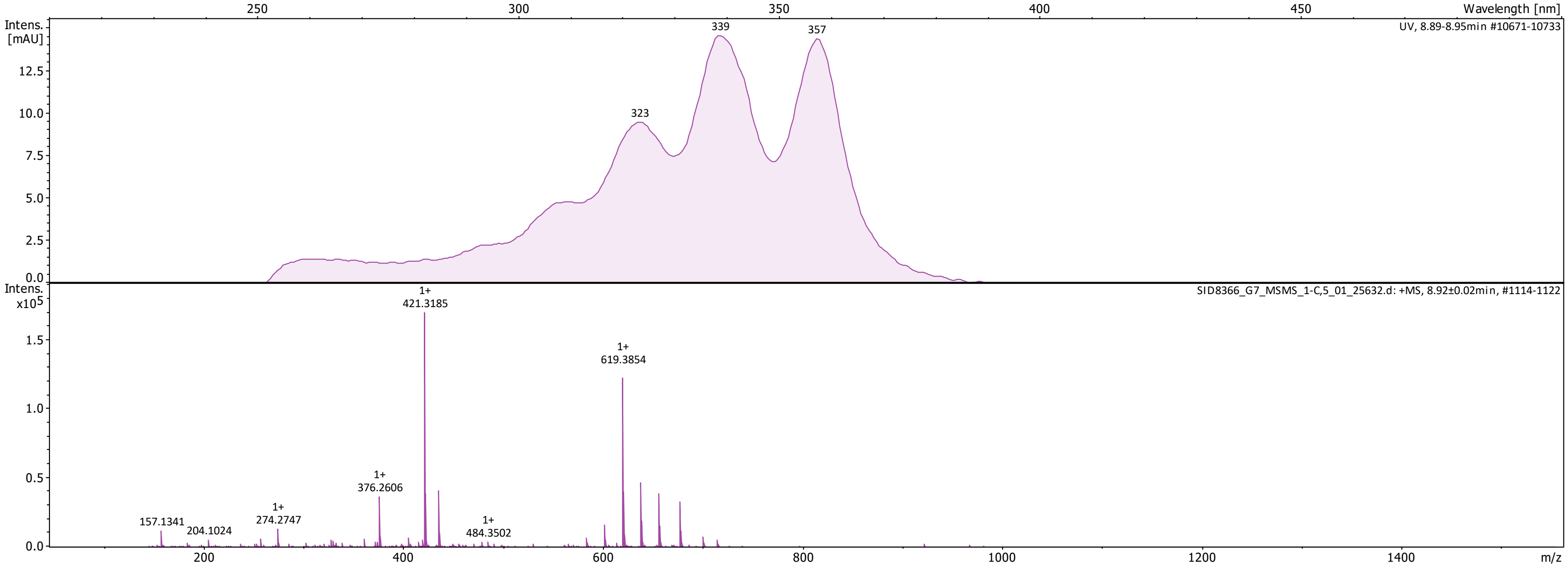

SID8366 – G7 LC-UV-MS/MS (*Rt* = 9.15 – 9.20 min):

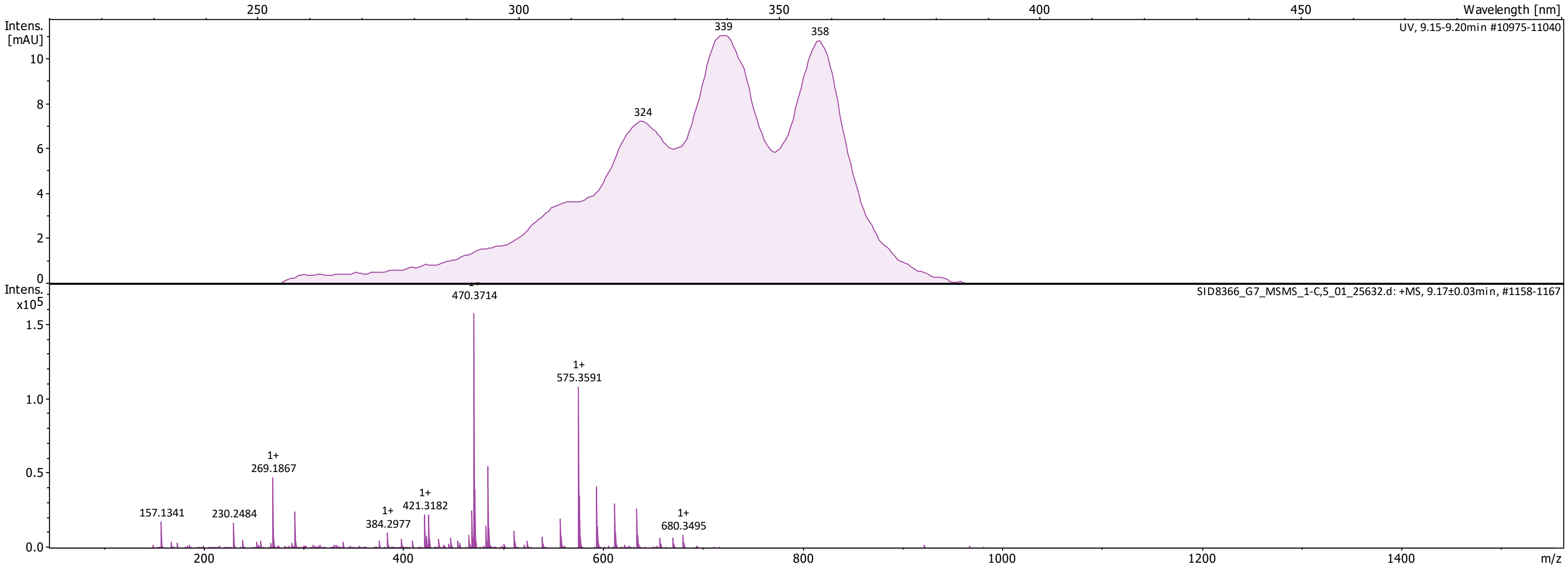

SID11285 – E8 LC-UV-MS/MS (*Rt* = 9.34 – 9.39 min):

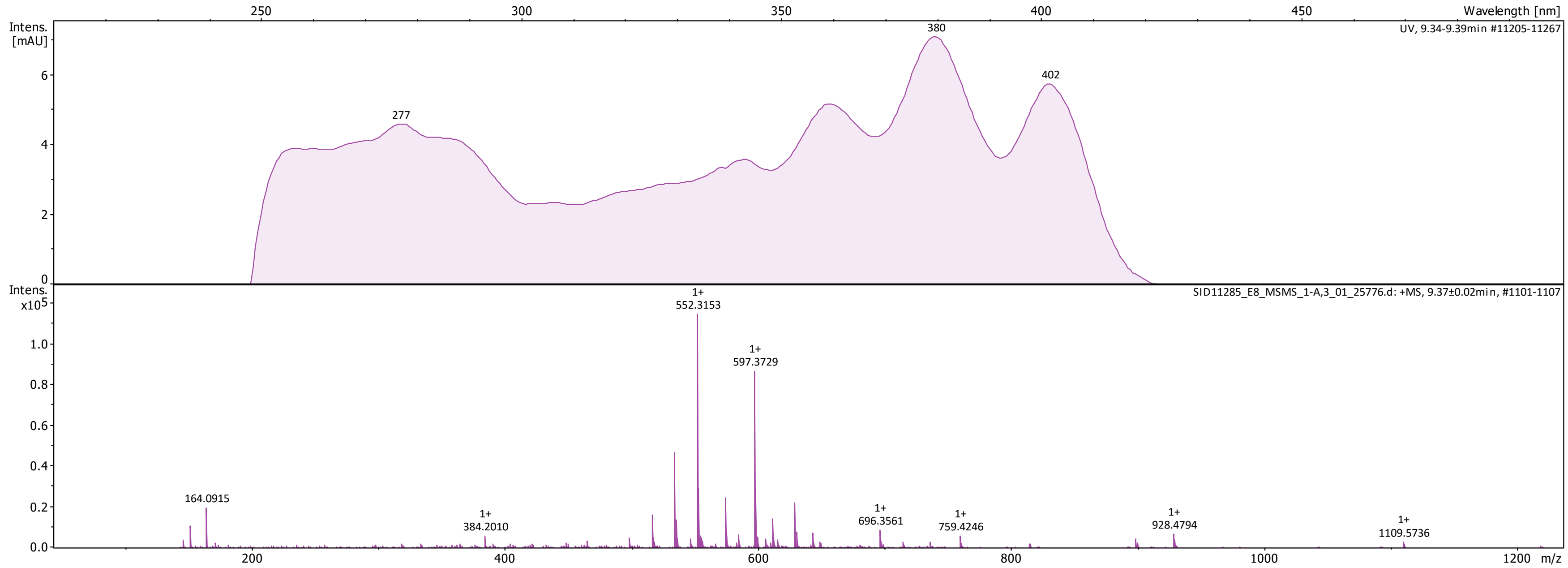

SID11285 – E8 LC-UV-MS/MS (*Rt* = 9.65 – 9.69 min):

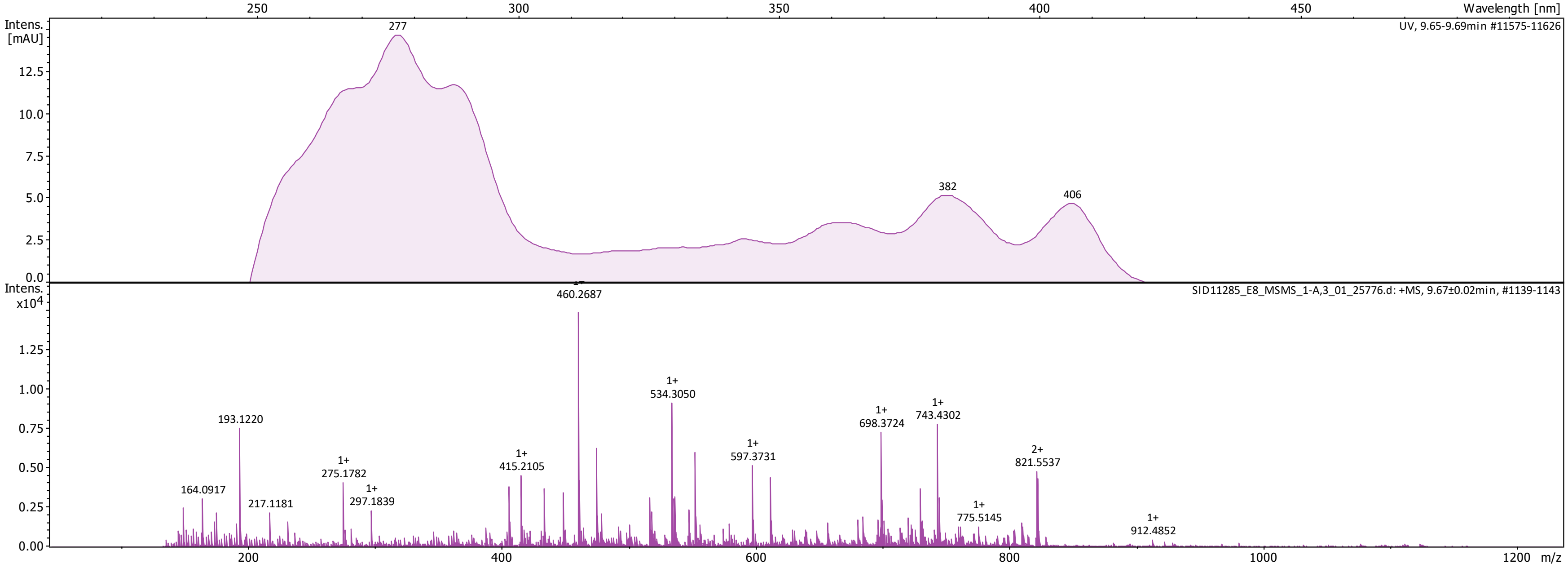

SID11285 – G7 LC-UV-MS (*Rt* = 9.46 – 9.52 min):

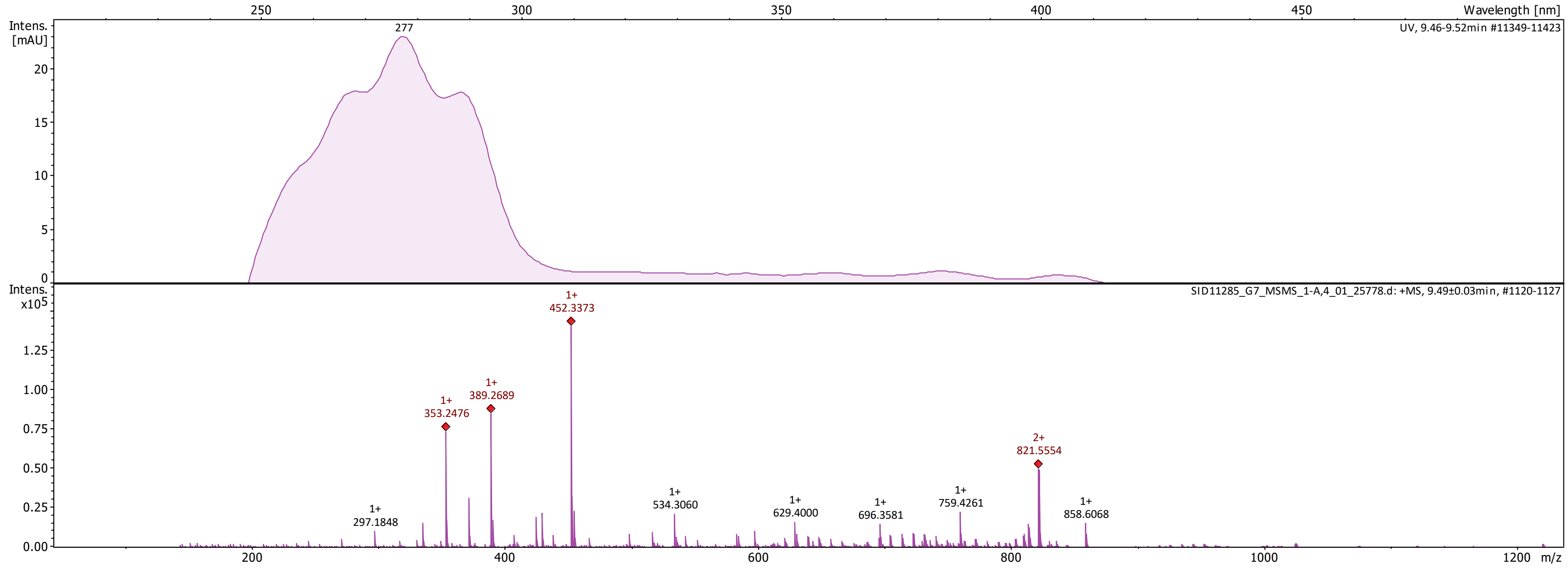

Dataset S4.1. SID7958-F6:

These data constitute the dereplication of bacterial extract SID7958-F6 using GNPS library searching with a mirror match to show cosine similarity. Presented below is experimental spectrum of SID7958-F6 (black) and the GNPS spectrum CCMSLIB00000851861 (green) for Monactin with a cosine similarity of 0.805.

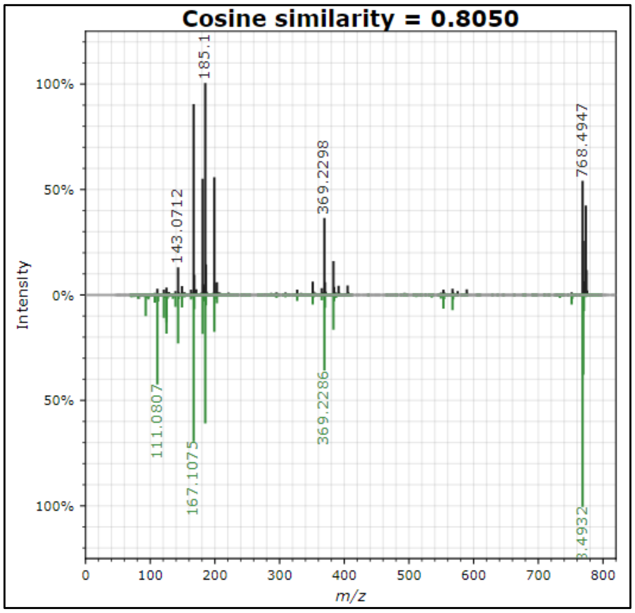

Dataset S4.2. SID7958-H8:

These data constitute the dereplication of bacterial extract SID7958-H8 using GNPS library searching with a mirror match to show cosine similarity. Presented in panel A is the experimental spectrum of SID7958-H8 (black) and the GNPS spectrum CCMSLIB00000851861 for Monactin (green) with a cosine similarity of 0.6982. Presented in B is the experimental spectrum of SID7958-H8 (black) and GNPS spectrum CCMSLIB00010109074 (green) for Nonactin with a cosine similarity of 0.8551.

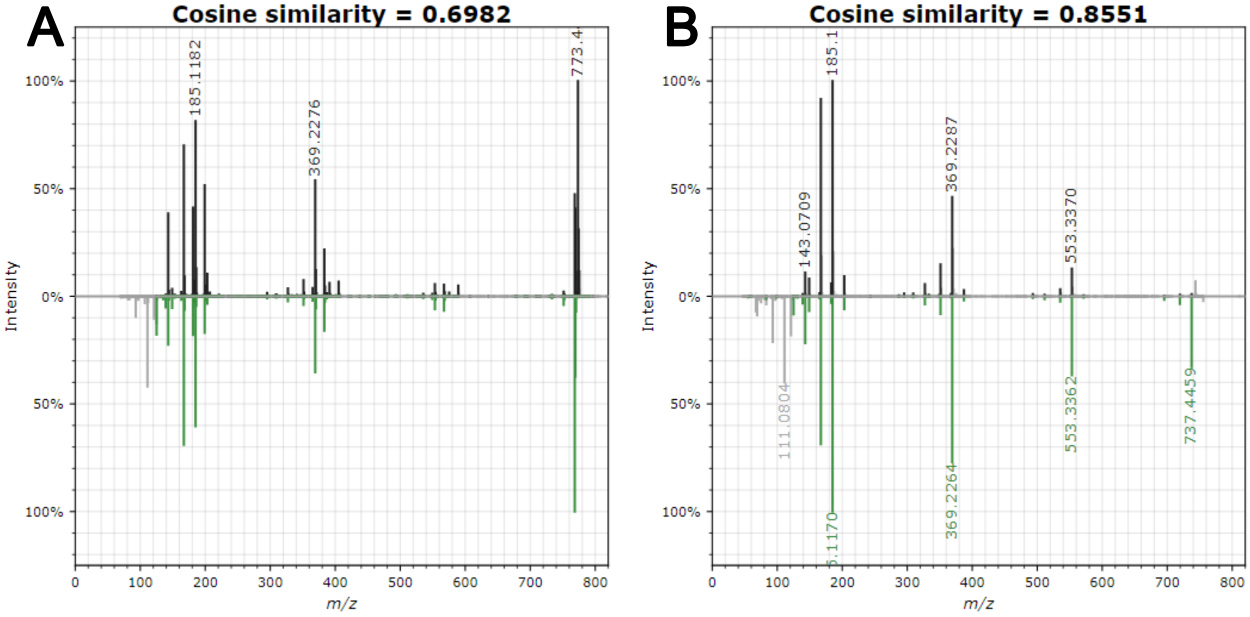

Dataset S4.3. SID8465 – H6:

These data constitute the dereplication of bacterial extract SID8465-H6 using GNPS library searching with a mirror match to show cosine similarity. Presented in panel A is the experimental spectrum of SID8465-H6 (black) and the GNPS spectrum CCMSLIB00000851861 (green) for Monactin with a cosine similarity of 0.8455. Presented in B is experimental spectrum of SID8465-H6 (black) and the GNPS spectrum CCMSLIB00000856076 (green) for Dinactin with a cosine similarity of 0.7409. Presented in C is experimental spectrum of SID8465-H6 (black) and the GNPS spectrum CCMSLIB00000854224 (green) for Trinactin with a cosine similarity of 0.7286. Presented in panel D is the experimental spectrum of SID8465-H6 (black) and the GNPS spectrum CCMSLIB00010109074 (green) for Nonactin with a cosine similarity of 0.8731.

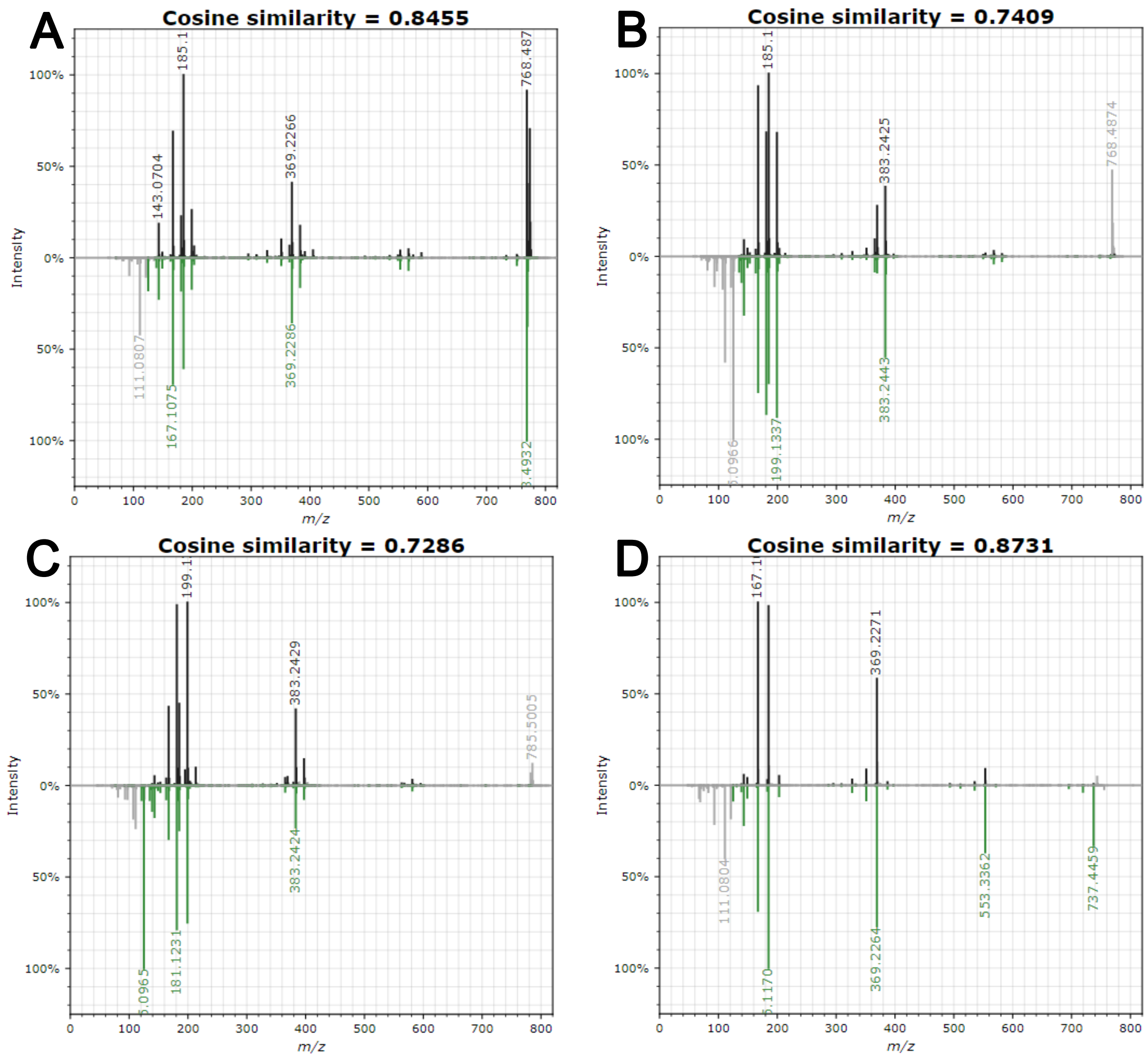

Dataset S4.4. SID9913 – H5:

These data constitute the dereplication of bacterial extract SID9913-H5 using GNPS library searching with a mirror match to show cosine similarity. Presented in A is the experimental spectrum of SID9913-H5 (black) and the GNPS spectrum CCMSLIB00000851861 (green) for Monactin with a cosine similarity of 0.8434. Presented in B is the experimental spectrum of SID9913-H5 (black) and the GNPS spectrum CCMSLIB00000856076 (green) for Dinactin with a cosine similarity of 0.8025. Presented in C is the experimental spectrum of SID9913-H5 (black) and the GNPS spectrum CCMSLIB00000854224 (green) for Trinactin with a cosine similarity of 0.7269. Presented in D is the experimental spectrum of SID9913-H5 (black) and the GNPS spectrum CCMSLIB00010109074 (green) for Nonactin with a cosine similarity of 0.8521.

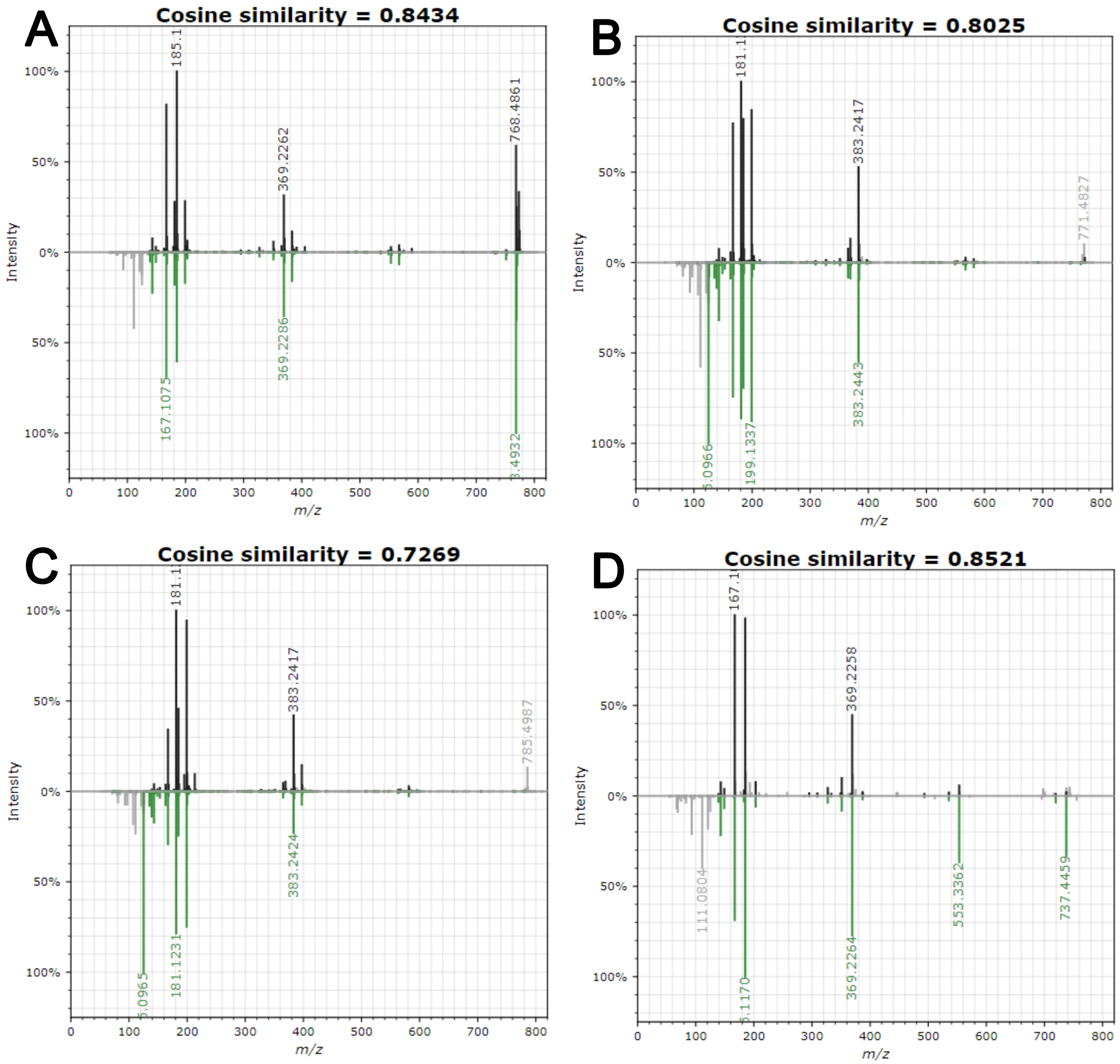

Dataset S4.5. SID9913 – H6:

These data constitute the dereplication of bacterial extract SID9913-H6 using GNPS library searching with a mirror match to show cosine similarity. Presented in A is experimental spectrum of SID9913-H6 (black) and the GNPS spectrum CCMSLIB00000851861 (green) for Monactin with a cosine similarity of 0.8347. Presented in B is experimental spectrum of SID9913-H6 (black) and the GNPS spectrum CCMSLIB00000856076 (green) for Dinactin with a cosine similarity of 0.7973. Presented in C is experimental spectrum of SID9913-H6 (black) and the GNPS spectrum CCMSLIB00000854224 (green) for Trinactin with a cosine similarity of 0.7246. Presented in D is experimental spectrum of SID9913-H6 (black) and the GNPS spectrum CCMSLIB00010109074 (green) for Nonactin with a cosine similarity of 0.8719.

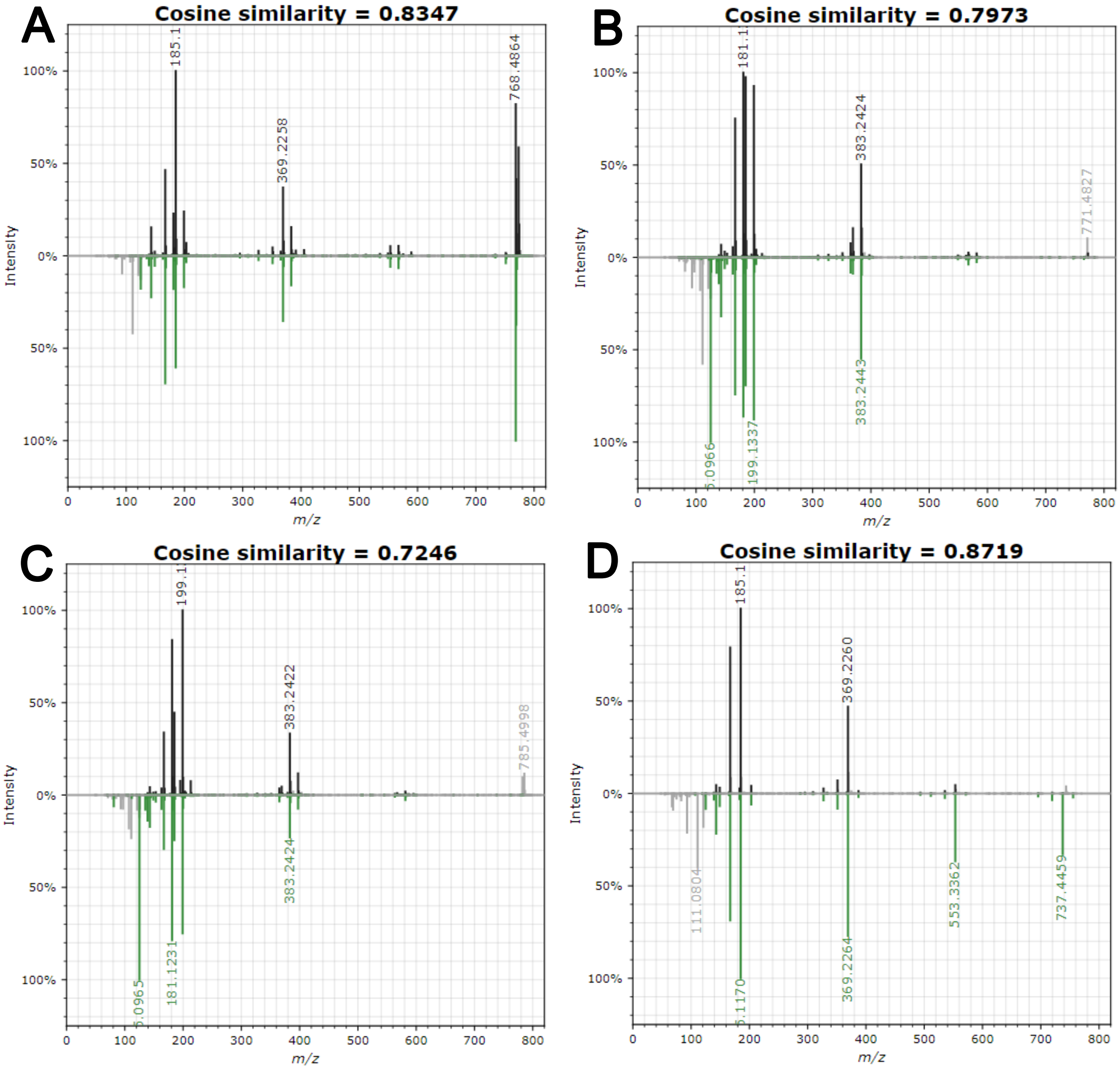

.

Dataset S5 - Comprehensive heatmap data from which manuscript **Figures 2** and **4** as well as Supplementary Materials **Figures S1**-**S4** were derived. Shown on the pages that follow are the full heatmaps for experiments detailing YCG data for the azole antifungals (**Dataset S5.1**), micafungin and MMS (**Dataset S5.2**), and assorted strains containing suspected polyenes alongside the polyene nystatin and positive control micafungin (**Dataset S5.3**).

The UW SMSF uses an internal research sample coding system and all heatmaps indicated within this dataset employ that coding system along the horizontal sample axis. Strain names employed within the manuscript are depicted in this dataset using the UW SMSF designators as indicated below. In addition, all comprehensive heatmap samples employing known antifungal agents or other controls are color coded for clarity and ease of visualization as indicated below.

heatmap sample name (top axis)	strain name
SMSSF-0569223	SID8366-E6
SMSSF-0570771	SID4921-G6
SMSSF-0619252	SID11285-B8
SMSSF-0570691	SID4915-G6
SMSSF-0570672	SID4915-E7
SMSSF-0523573	WMMC500-E6
SMSSF-0619301	SID11285-G7
SMSSF-0619282	SID11285-E8
SMSSF-0570673	SID4915-E8
SMSSF-0524705	SID6-F8
SMSSF-0619155	SID11233-H11
SMSSF-0618564	SID7982-E10
SMSSF-0569226	SID8366-E9

Color code for standards:

- Micafungin
- Micafungin spike
- Itraconazole
- Itraconazole spike
- Voriconazole
- Voriconazole spike
- MMS
- Nystatin

Dataset 5.1 – Comprehensive heatmap data supporting data/experiments depicted in Figure 2A. Shown are results of YCG experiments with itraconazole, voriconazole, and positive controls MMS and micafungin. Black boxes indicate strain abundance relative to DMSO controls, green indicates reduced abundance (hypersensitivity), red indicates increased abundance (resistance) and gray = absence or below "cutoff" for detection.

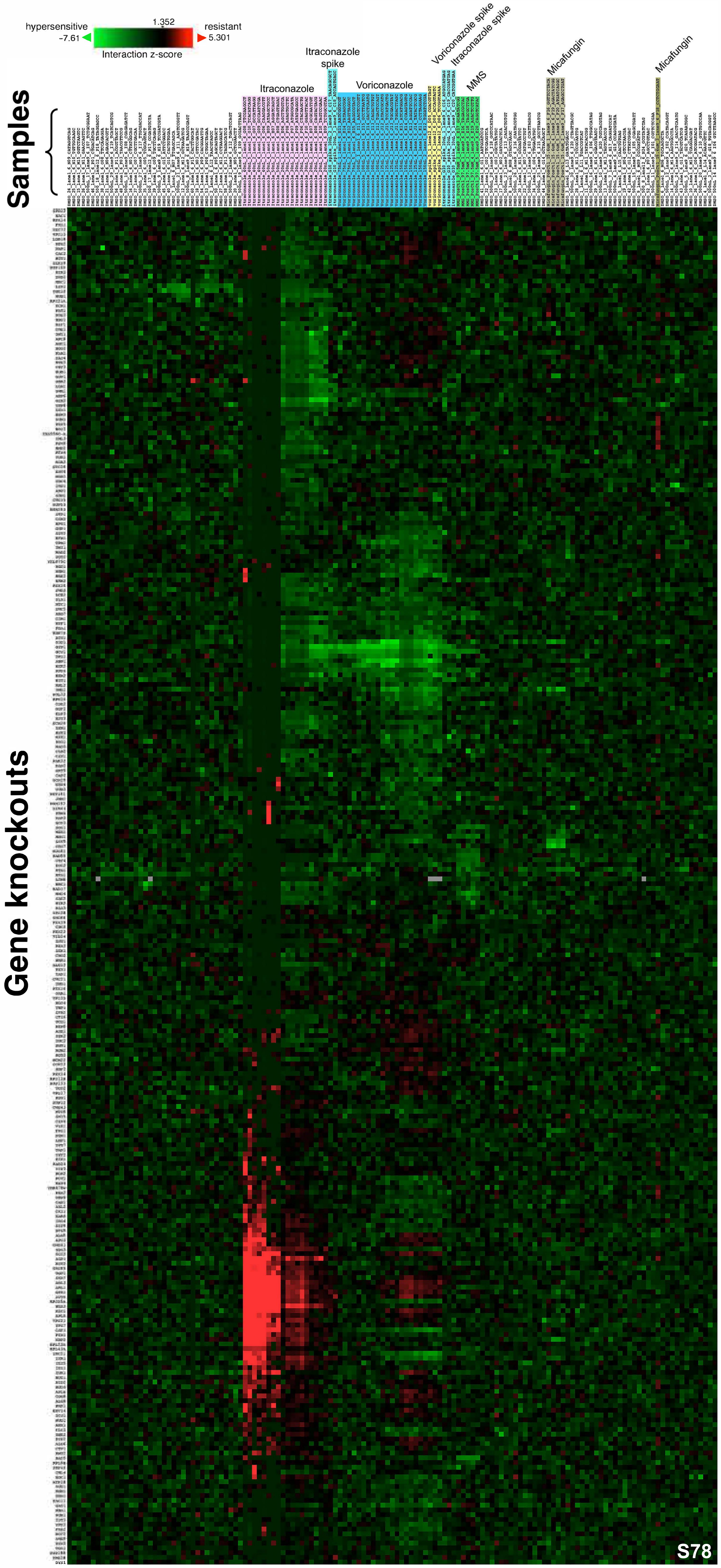

Dataset 5.2 – Comprehensive heatmap data supporting data/experiments depicted in Figure 2B. Black boxes indicate strain abundance relative to DMSO controls, green indicates reduced abundance (hypersensitivity), red indicates increased abundance (resistance) and gray = absence or below "cutoff" for detection.

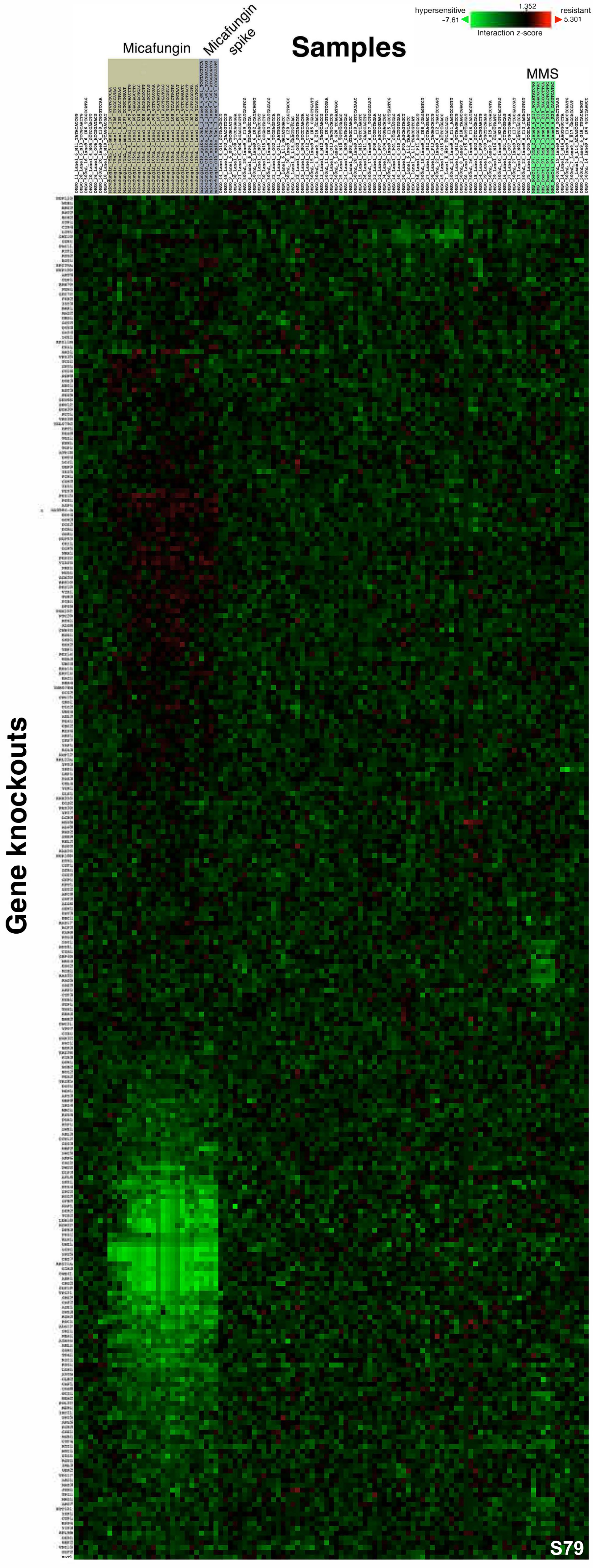

Dataset 5.3 – Comprehensive heatmap data supporting data/experiments detailed in Figure 4 and especially relating to polyene characteristics. Black boxes indicate strain abundance relative to DMSO controls, green indicates reduced abundance (hypersensitivity), red indicates increased abundance (resistance) and gray = absence or below "cutoff" for detection.

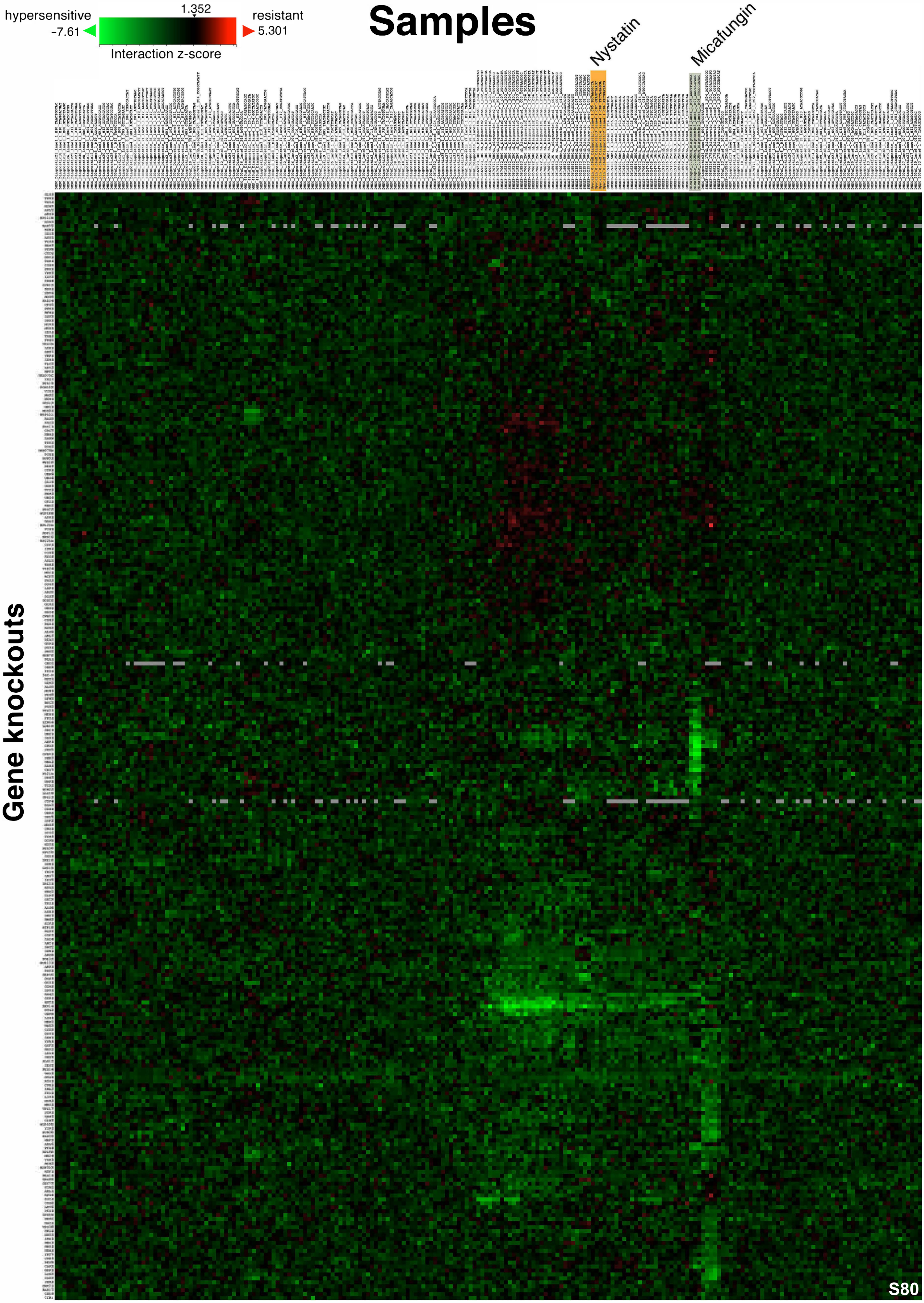

Supplement: Supplementary file 1 [file molecules-30-00077-s001.zip › molecules-3344164-supplementary.pdf]
